# Stereoselective Biocatalyzed Reductions of Ginger Active Components Recovered from Industrial Wastes

**DOI:** 10.1002/cbic.202200105

**Published:** 2022-03-03

**Authors:** Rita Nasti, Ivan Bassanini, Erica Elisa Ferrandi, Federica Linguardo, Susanna Bertuletti, Marta Vanoni, Sergio Riva, Luisella Verotta, Daniela Monti

**Affiliations:** ^1^ Department of Environmental Science and Policy Università degli Studi di Milano Via Celoria 2 Milano 20133 Italy; ^2^ Istituto di Scienze e Tecnologie Chimiche “Giulio Natta” Consiglio Nazionale delle Ricerche Via Mario Bianco 9 Milano 20131 Italy

**Keywords:** biocatalysis, challenging ketones, ginger active components, stereoselectivity, waste valorization

## Abstract

Ginger is among the most widespread and widely consumed traditional medicinal plants around the world. Its beneficial effects, which comprise e. g. anticancer and anti‐inflammatory activities as well as gastrointestinal regulatory effects, are generally attributed to a family of non‐volatile compounds characterized by an arylalkyl long‐chained alcohol, diol, or ketone moiety. In this work, ginger active components have been successfully recovered from industrial waste biomass of fermented ginger. Moreover, their recovery has been combined with the first systematic study of the stereoselective reduction of gingerol‐like compounds by isolated alcohol dehydrogenases (ADHs), obtaining the enantioenriched *sec*‐alcohol derivatives *via* a sustainable biocatalytic path in up to >99 % conversions and >99 % enantiomeric/diastereomeric excesses.

## Graphical abstract

**Ginger active** constituents have been successfully recovered from industrial waste biomasses of fermented ginger and converted into the corresponding diols and alcohols by stereoselective biocatalyzed reductions.

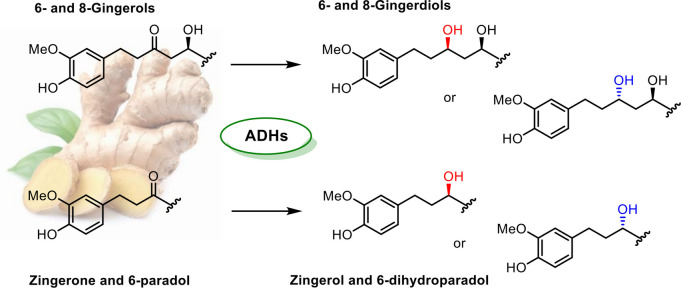

## Introduction

**Figure 1:**
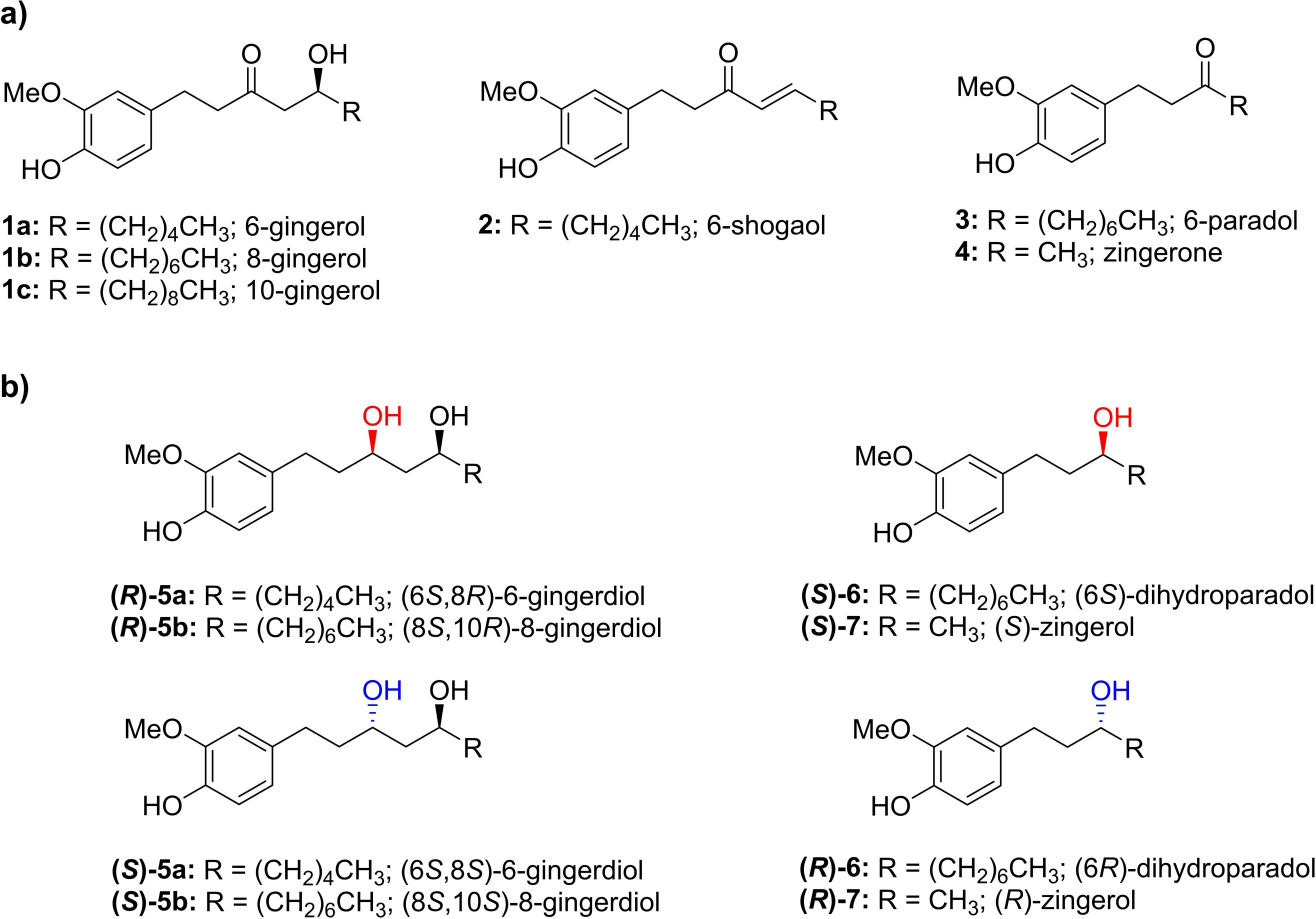
Structures of main ginger active components (a) and optically active diols and alcohol derivatives obtained in this study (b).

Ginger root (*Zingiber officinale*, Roscoe, Zingiberaceae) is one of the main plants used in the food, nutraceutical, and pharmaceutical field.^[1]^ It is a rhizome widely consumed all around the world and, since ancient times, was used in many cultures not only as a food but even as traditional medicine, especially in Asia and Africa. The possible beneficial properties of ginger, including anticancer and anti‐inflammatory activities, have been widely studied[^1^, ^2^, ^3^, ^4^, ^5^, ^6^, ^7^, ^8^] and generally addressed to non‐volatile constituents named gingerols and shogaols (Figure 1a). Recently, other minor compounds, like 6‐paradol[^9^, ^10^] and zingerone[^11^, ^12^] have shown interesting bioactivity profiles as well. Moreover, ginger is included in the list of 51 medicinal plants identified by The Committee on Herbal Medicinal Products (HMPC) for gastrointestinal disorder treatment (https://www.ema.europa.eu/en/medicines/download‐medicine‐data). Recent *in vivo* studies have demonstrated the beneficial involvement of short‐term intake of ginger on the composition and function of gut microbiota in healthy people^[13]^ and on obese mice.^[14]^ Additionally, semisynthetic derivatives of gingerols and related compounds show interesting potential in drug development studies.^[15]^

For all of these reasons, the ginger rhizome is a substrate of several food processes such as i) dehydration to obtain dried ginger powder form (for food supplements, condiments, sausages, etc.), ii) extraction of oleoresin with food compatible solvents (for personal care, flavoring of beverages, and perfumes) and iii) fermentation to obtain aromatic beverages. The last two processes generate heaps of waste that are conventionally incinerated or employed for agriculture scope (fertilizer, animal feed). Alternative utilization of this biomass has recently emerged in the fields of paper‐making, biorefinery, and waste‐water treatments,[^16^, ^17^] following the same fate already depicted for other biomasses.

Few studies have been aimed at valorizing the bioactive molecules in agri‐food waste that may still have pharmacological properties. Accordingly, to the best of our knowledge, limited work has been performed concerning the recovery or chemical manipulation of gingerol‐like compounds from ginger residue.

As an example, among the possible derivatives that can be prepared from waste ginger biomass,^[15]^ 6‐gingerdiols, the diols formally obtained by the stereoselective reduction of 6‐gingerol (Figure 1b), have been recently reported as the major (and active) metabolites produced from the administration of 6‐gingerol to different human cancer cell lines.^[18]^ The reported antiproliferative activity of 6‐gingerdiols have thus raised interest toward the development of simple and selective synthetic strategies for their preparation as enantiomerically enriched species to be applied in metabolite studies, or as novel, antiproliferative lead compounds of natural origin.^[19]^ Moreover, given the plethora of bioactivities which have been connected to ginger extracts, a biocatalytic entry to gingerol‐like compounds, like 6‐shogaol and its reduced derivatives paradol and dihydroparadol, could help disclose their profiles as bioactive compounds and assess their, potential, cytotoxicity.[^2^, ^20^]

In general, a key to the success of enantioselective reduction of prochiral ketones is the presence of sterically and/or electronically different Cα‐ and Cα’‐substituents. However, this structural feature is absent in this class of polyphenols, thus they may be classified as “*challenging ketones*”. Accordingly, a literature search did not provide reports about the enantioselective reduction of gingerol‐like compounds through asymmetric synthesis. On the contrary, the total synthesis is currently the favored strategy to achieve the enantioenriched *sec*‐alcohol derivatives.[^21^, ^22^] It is worth mentioning that the preparation of selected gingerdiols has been recently achieved by Markad *et al. via* an elegant total synthesis starting from *n*‐heptanal instead of seeking for a semi‐synthesis using natural 6‐gingerol (**1 a**, Figure 1a) as starting material. Specifically, an iterative proline‐catalyzed α‐aminoxylation, followed by Horner‐Wadsworth‐Emmons or Wittig olefination reactions, represented the key steps of the proposed synthesis which afforded the two diastereomeric gingerdiols with excellent diastereoselectivity by using D‐ or L‐proline. However, the synthesis included 12 linear steps and the overall yields were around 18 %.^[23]^

As far as enzyme‐catalyzed stereoselective reduction of gingerol‐like compounds concerns, there are only a few reports so far. For example, the reduction of zingerone (**4**) by different fungal strains has been recently investigated by Svetaz *et al*.^[24]^ The biotransformation of **1 a** and 6‐shogaol (**2**) by *Aspergillus niger* strains to reduced derivatives has been reported as well.[^25^, ^26^]

However, these studies were mainly focused on the definition of metabolite profiles and in monitoring their changes in the culture media according to incubation times.

Herein, the recovery of ginger active constituents from industrial wastes of fermented ginger has been combined with the first systematic study of the stereoselective reduction of gingerol‐like compounds by isolated alcohol dehydrogenases (ADHs), thus obtaining the enantioenriched *sec*‐alcohol derivatives *via* a sustainable path.

## Results and Discussion

### Extraction and characterization of fresh and fermented ginger

Ginger lees from an industrial alcoholic fermentation process were provided by a local farm (Az. Agricola Prela Alba, Morbegno (SO), Italy). To evaluate the possible effect of the fermentation process on the ginger rhizome, the analyses were performed in parallel with fresh ginger.

Both dried ginger lees and fresh ginger were thus extracted with the same procedure to compare the chemical profile and gingerol‐like compounds content. Despite the different origin of the biomasses, the gingerol content in not treated ginger rhizome is never less than 5–6 % as evidenced by the literature.^[27]^ Moreover, the chemical profile of nonvolatile oil seems be congruent in rhizome with different origins, except for minor compounds. The intent of this comparison was to highlight differences in the chemical composition as direct consequence of the fermentation process. It is worth noting that processes such as storage, steaming, and cooking can affect the gingerol content and the extraction yield.[^28^, ^29^]

The two vegetal matrixes were extracted by dichloromethane (DCM) to obtain a residue rich in all gingerol‐like compounds without polar interfering agents (sugars, amino acids, etc.). The extraction yield of fresh ginger was 6.0 % w/w, similar to that obtained by Kikuzaki *et al*.^[30]^ As expected, the oleoresin extracted from ginger lees was much lower (2.3 % w/w). Most likely, part of oleoresin was extracted by the alcoholic fraction generated by fermentation and passed in the beverage.

**Figure 2:**
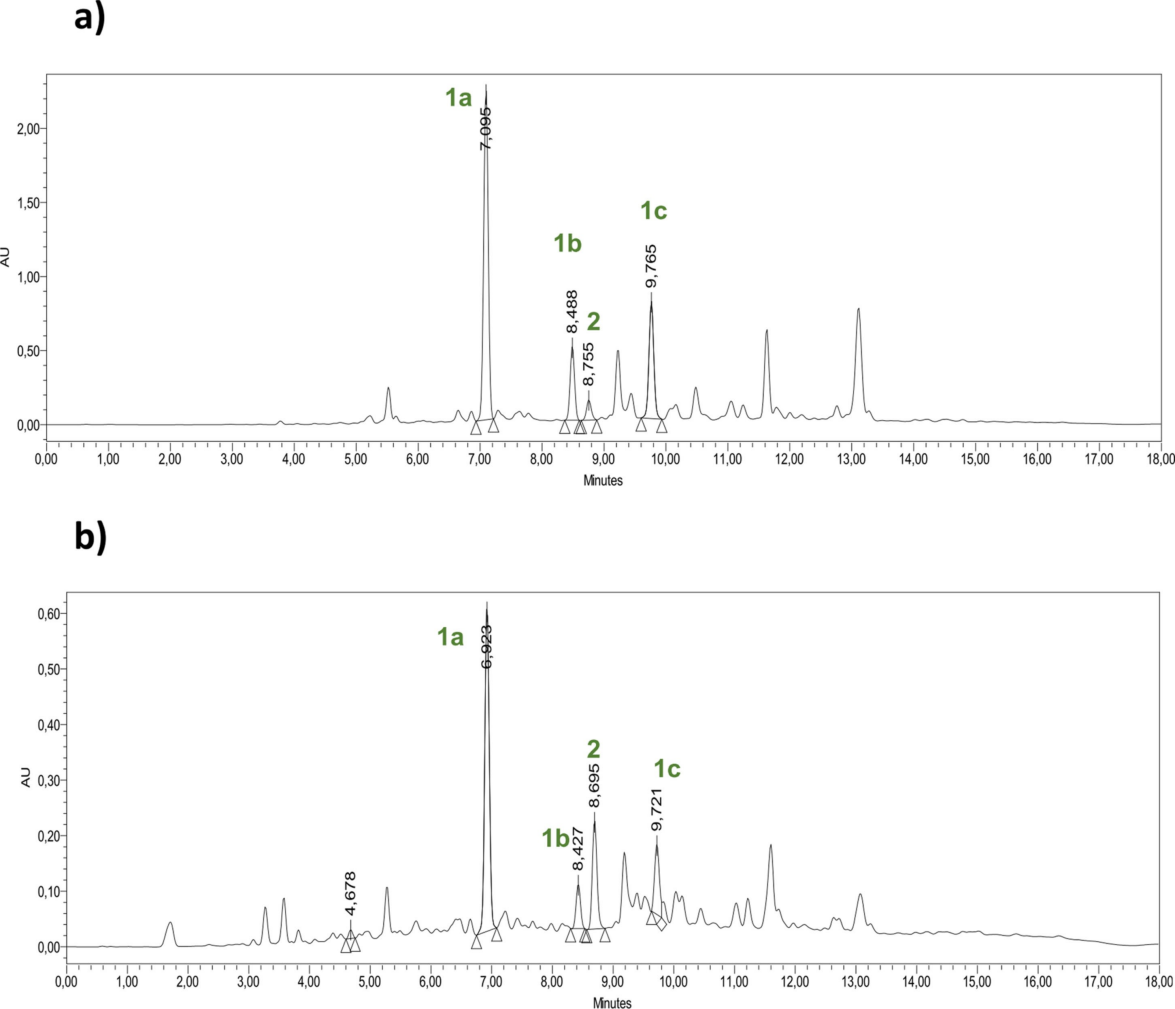
Extraction of gingerol‐like compounds from fresh ginger (a) and ginger fermentation residues (b).

Concerning ginger active components, both extracts show similar chemical profiles, where the main species detected are 6‐gingerol (**1 a**), 8‐gingerol (**1 b**), 10‐gingerol (**1 c**), and 6‐shogaol (**2**) (Figure 2). Their identities were confirmed by comparing their mass spectra, molecular formulae, and related accurate molecular mass data with literature data.^[31]^

Interestingly, the result of ginger content by quantitative UPLC analysis shows that fermented ginger has a lower amount of gingerols than fresh ginger. In particular, **1 a** was the main compound in both extracts, but resulted to be almost half in fermented ginger (5.4 %) than in fresh ginger (11.4 %) (Table 1).

**Table 1:** Characterization and yields of gingerol‐like compounds from fresh ginger and ginger lees.

Compound	Retention time [min]	(−)‐ESI‐MS [m/z]	Fresh ginger [% w/w]	Ginger lees [% w/w]
**1 a**	7.15	293.61	11.4	5.43
**1 b**	8.49	321.47	2.69	0.94
**2**	8.75	275.39	2.39	1.31
**1 c**	9.76	349.47	4.50	1.66

### Preparation of racemic standards

Diols and alcohols from gingerol‐like compounds (Figure 1b) are not commercially available and synthesizing the corresponding racemic standards was mandatory for our following work.

For this purpose, compounds **1 a** and **1 b** were isolated from the ginger extract in a suitable yield for the preparation of corresponding alcohols (see Experimental Section). Compound **3** was obtained from **2** by the biocatalyzed reduction of the carbon‐carbon double bond with a selected ene‐reductase (for biotransformation details, see paragraph “Performances of selected ADHs in the stereoselective reduction of **1 b** and **3**”). Contrarily, zingerone (**4**) is commercially available and was used as reference compound for all screenings.

The racemic reference compounds were easily obtained by reduction of **1 a**–**b**, **3**, and **4** in the presence of sodium borohydride (NaBH_4_) in less than 30 min with a good yield (91–96 %).

The spectral analysis (^1^H‐NMR and ^13^C‐NMR, see Supporting Information) confirmed the presence of the reduced derivatives 6‐dihydroparadol (**7**) and zingerol (**8**) (Figure 1b). The racemic compounds were analyzed by chiral phase HPLC equipped with Chiralcel® OD‐H column to assign the absolute configuration related to elution order according to Svetaz *et al*.^[24]^

In the case of 6‐gingerdiols, the *RS*‐diastereomer was prevalent in the mixture (*de*: 70 : 30, *RS*:*SS*). For the characterization of diastereoisomeric mixture of compound **(*R*)‐5 a** and **(*S*)‐5 a**, ^1^H‐NMR and ^13^C‐NMR spectra were compared with data reported from Sabitha *et al*.^[32]^ In particular, ^13^C‐NMR (see Supporting Information) spectrum was fundamental to determine unequivocally the different peaks relatives to the carbons of the four stereocenters. According to the literature, the stereogenic carbons were found at δ 73.28 (C6) and δ 72.36 (C8) ppm and δ 69.54 (C6) and δ 68.91 (C8) ppm, for **(*R*)‐5 a** and **(*S*)‐5 a**, respectively.

### Screening of alcohol dehydrogenases (ADHs) in the stereoselective reduction of 1 a and 4

The first part of the screening of the biocatalyzed reduction of **1 a** and **4** was carried out by testing a library of 23 different alcohol dehydrogenases (ADHs)^[33]^ including commercially available ADHs, i. e., ADH recombinant from *E. coli* (Ec‐ADH), *Thermoanaerobium brockii* ADH (Tb‐ADH), *Candida boidinii* ADH (Cb‐ADH), *C. parapsilosis* ADH (Cp‐ADH), and horse liver ADH (HL‐ADH), as well as a set of enzymes from our in‐house collection (18 different biocatalysts, Table S1, Supporting Information).

Specifically, the biocatalysts library included ADHs which were previously studied in the stereoselective reduction of prochiral β‐diketones, for example the ADH from *Rhodococcus ruber* (Rr‐ADH),^[34]^ *Lactobacillus kefir* (Lk‐ADH)^[35]^ and *L. brevis* (Lb‐ADH),^[36]^ or in the reduction of keto derivatives of fatty acids, such as the ADH from *Micrococcus luteus* (Ml‐ADH),^[37]^ as well as Is2‐SDR, a wide‐substrate scope ADH recently discovered by us in an Icelandic hot spring metagenome,[^38^, ^39^] and a collection of ADHs active on steroidal substrates, namely hydroxysteroid dehydrogenases (HSDHs),^[40]^ recently investigated by us and showing interesting substrate promiscuity in the reduction of a panel of structurally different ketones.[^38^, ^39^, ^41^]

**Scheme 1:**
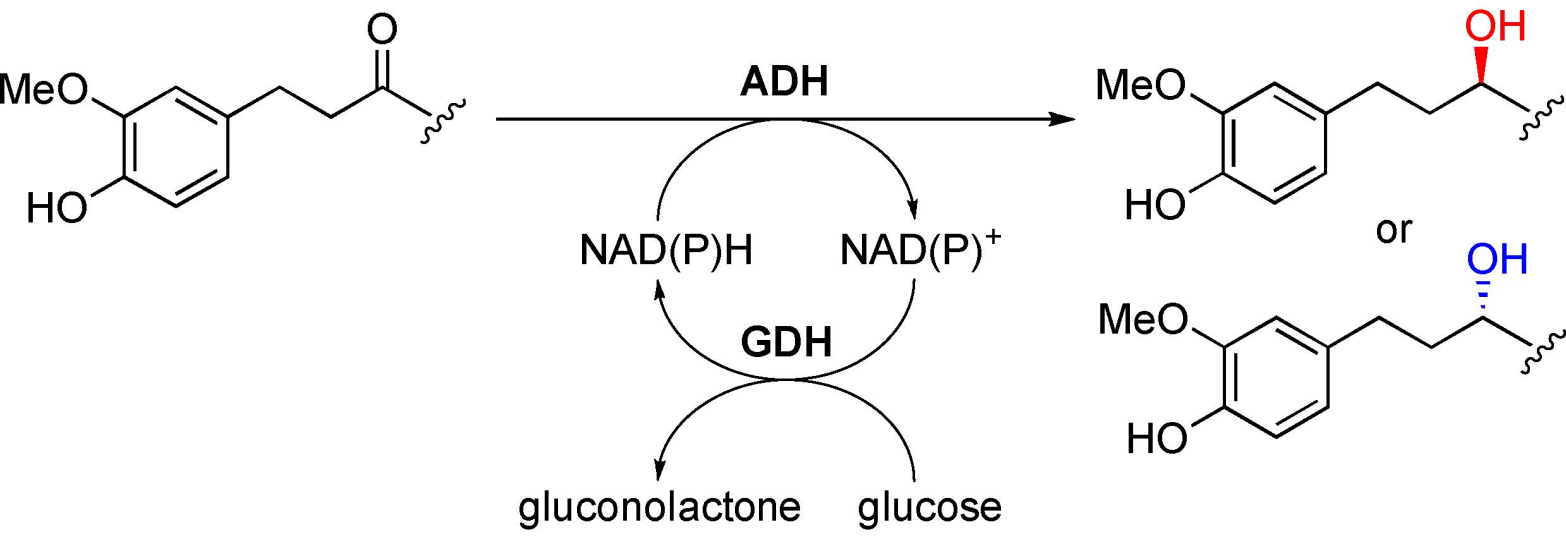
General scheme of the biocatalyzed reduction of ginger active compounds (**1 a**–**b**, **3** and **4**). The glucose/GDH system is used for cofactor regeneration.

The reduction reactions (1 mL final volume) were set up in the presence of a glucose/glucose dehydrogenase (GDH) system for the *in situ* regeneration of the NAD(P)H cofactor (Scheme 1).

Table 2 (entries 1–9) shows only the biotransformations leading to the detectable formation of the desired reduced product(s) from **1 a** and/or **4**. As evident, with our disappointment, several ADHs, and in particular, most of the tested HSDHs, were not active in the reduction of either the two substrates. Among all the different enzymes tested, an interesting result was obtained only with Is2‐SDR (entry 9), which could reduce both substrates, although with relatively modest conversions and not very high enantiomeric/diastereomeric excess (ee/de) values.

**Table 2:** Screening of alcohol dehydrogenases in the stereoselective reduction of **1 a** and **4**.

Entry	Enzyme^[a]^	Substrate			
		**4**		**1 a**	
		*c* [%]^[b]^	*ee* [%]^[b]^	*c* [%]^[b]^	*de* [%]^[b]^
1	Ec‐ADH	>99	99 (*S*)	–	–
2	Tb‐ADH	19	61 (*R*)	–	–
3	HL‐ADH	8	15 (*R*)	–	–
4	Ml‐ADH^[c]^	–	–	96	97 (*R*)
5	Lk‐ADH^[d]^	68	98 (*R*)	–	–
6	Rr‐ADH	>95	>99 (*S*)	–	–
7	Lb‐ADH^[c]^	>95	>99 (*R*)	–	–
8	Cp‐ADH	18	52 (*R*)	–	–
9	Is2‐SDR^[c]^	24	83 (*S*)	50	>95 (*R*)
10	evo‐1.1.010^[e]^	54	87 (*S*)	>99	91 (*R*)
11	evo‐1.1.020^[e]^	96	97 (*S*)	>99	94 (*R*)
12	evo‐1.1.030^[e]^	>99	>99 (*S*)	12	>99 (*R*)
13	evo‐1.1.040^[e]^	54	34 (*S*)	–	–
14	evo‐1.1.130^[e]^	26	67 (*S*)	13	>99 (*R*)
15	evo‐1.1.140^[e]^	<10	–^[f]^	–	–
16	evo‐1.1.190^[e]^	18	48 (*S*)	–	–
17	evo‐1.1.200^[e]^	>99	>99 (*R*)	88	6 (*S*)
18	evo‐1.1.210^[e]^	>99	51 (*S*)	<10	–^[f]^
19	evo‐1.1.250^[e]^	52	<5 (*R*)	90	>99 (*S*)
20	evo‐1.1.260^[e]^	85	88 (S)	18	>99 (*R*)
21	evo‐1.1.270^[e]^	>99	>99 (*R*)	50	>99 (*R*)
22	evo‐1.1.380^[e]^	36	28 (*S*)	32	>99 (*S*)
23	evo‐1.1.420^[e]^	>99	>99 (*R*)	<5	–^[f]^
24	evo‐1.1.430^[e]^	56	60 (*S*)	<5	–^[f]^
25	evo‐1.1.440^[e]^	>99	>99 (*S*)	>99	74 (*R*)
26	evo‐1.1.441^[e]^	>99	61 (*R*)	46	66 (*S*)
27	evo‐1.1.442^[e]^	>99	98 (*S*)	<10	–^[f]^

[a] For details about enzymes source and production see Supporting Information. [b] Conversions and enantiomeric/diastereomeric excesses (ee values/de values) determined by chiral phase HPLC analysis (Method C, see Experimental Section for details) after 72 h at 30 °C if not stated otherwise. [c] Reactions performed at 25 °C. [d] Reactions performed at 20 °C in the presence of 5 mg mL^−1^ purified Lk‐ADH. [e] Reactions performed according to the manufacturer (evoxx technologies GmbH, see Experimental Section for details). [f] Not determined, below detection limit.

More interesting results were instead obtained in the reduction of **4** with different ADHs. In particular, excellent conversions and ee values were achieved with the commercially available biocatalyst Ec‐ADH (Table 2, entry 1), as well as with the recombinant enzymes Rr‐ADH and Lb‐ADH (entries 6 and 7, respectively). The latter biocatalysts showed also exquisite and opposite stereoselectivity, thus providing **(*S*)‐7** (Rr‐ADH) and **(*R*)‐7** (Lb‐ADH), in both cases with >99 % ee. Good results, but to a lower extent, were observed also with Lk‐ADH (entry 5, conv. 68 %, ee 98 %). Remarkably, these outcomes resemble, in terms of stereoselectivity, those obtained by Baer *et al*.^[42]^ with Rr‐ADH and Lk‐ADH in the biocatalytic reduction of (*S*)‐ and (*R*)‐4‐(4‐chlorophenyl)‐4‐hydroxybutan‐2‐one, compounds having a structural similarity with gingerol‐like compounds.

Coming to the screening of **1 a** reduction, besides the previously mentioned result obtained with Is2‐SDR (entry 9), quite surprisingly none of the ADHs showing high activity and selectivity toward **4** was similarly capable to accept this much bulkier substrate. Instead, an excellent result was achieved with Ml‐ADH (entry 4), which afforded the (6*S*,8*R*) diastereoisomer **(*R*)** *‐* **5 a** with 96 % conv. and 97 % diastereoisomeric excess. As previously mentioned, in contrast to most of the ADHs used in this work, Ml‐ADH was reported as a secondary alcohol dehydrogenase capable to successfully reduce the ketone derivatives of hydroxy fatty acids^[37]^ and other long‐chain aliphatic substrates.^[43]^ However, to the best of our knowledge, the activity and selectivity of this enzyme toward arylalkyl long‐chained ketone derivatives, such as **1 a**, has not been reported before.

In the second part of the screening, the study was extended to a commercially available ADH screening kit (evoxx technologies GmbH), to further investigate both reduction reactions of **1 a** and **4**, and, possibly, find a biocatalyst capable to reduce **1 a** to the other possible diastereoisomer, i. e., **(*S*)** *‐* **5 a** (Figure 1b). As shown in Table 2 (entries 10–27), most of the tested biocatalysts showed activity toward at least one of the two substrates, thus demonstrating the broad substrate scope of this enzyme collection.

As far as the reduction of **4** concerns, excellent results were achieved with evo‐1.1.030, evo‐1.1.200, evo‐1.1.270, evo‐1.1.420, and evo‐1.1.440 (entries 12, 17, 21, 23, and 25, respectively), leading to the quantitative formation of either the (*S*)‐ or (*R*)‐alcohol in enantiopure form (see Supporting Information for details).

The performances of the evoxx ADHs in the reduction of **1 a** were in general less satisfactory, high conversions and des being observed only with evo‐1.1.010, evo‐1.1.020, evo‐1.1.250, and evo‐1.1.440 (entries 10, 11, 19, and 25, respectively). However, the outcome of evo‐1.1.250‐catalyzed reaction was remarkable, since the desired product **(*S*)** *‐* **5 a**, i. e., the (6*S*,8*S*) diastereoisomer (Figure 1b) was obtained with high conversions and excellent optical purity (entry 19).

### Performances of selected ADHs in the stereoselective reduction of 1 b and 3

Further investigations were carried out by using as substrates the compounds **1 b** and **3** (Figure 1a), both representing analogs of **1 a** and **4** with a longer aliphatic chain.

Interestingly, the secondary alcohol dehydrogenase Ml‐ADH resulted active in the reduction of the bulkier 8‐gingerol (**1 b**), leading to the formation of the **(*R*)** *‐* **5 b** diol, thus keeping the same stereoselectivity observed in the reduction of **1 a**. The (8*S*,10*R*) diastereoisomer was in fact obtained with excellent de (>99 %), although with lower conversions (53 %) when compared with **1 a** reduction (96 %, Table 2, entry 4).

Due to the very low availability of **3** from fermented ginger extracts, we decided to biosynthesize it from extracted **2** using an enzymatic approach to run a preparative biotransformation. Regarding the biocatalyzed synthesis of **3**, it should be pointed out that enones and enals are generally regarded as substrates not suitable for ADHs‐mediated reduction since they possess an α,β‐unsaturated carbonyl moiety. However, this functional group, characterized by an activated carbon‐carbon double bond, is of particular interest in biocatalysis especially for processes catalyzed by the enzymes ene reductases (ERs).^[44]^

ERs are, in fact, flavin‐dependent enzymes from the Old Yellow Enzyme (OYE) family that catalyze the asymmetric reduction of carbon‐carbon double bonds bearing electron withdrawing groups, converting *e. g*. enones into the corresponding saturated ketones.^[45]^

**Scheme 2:**
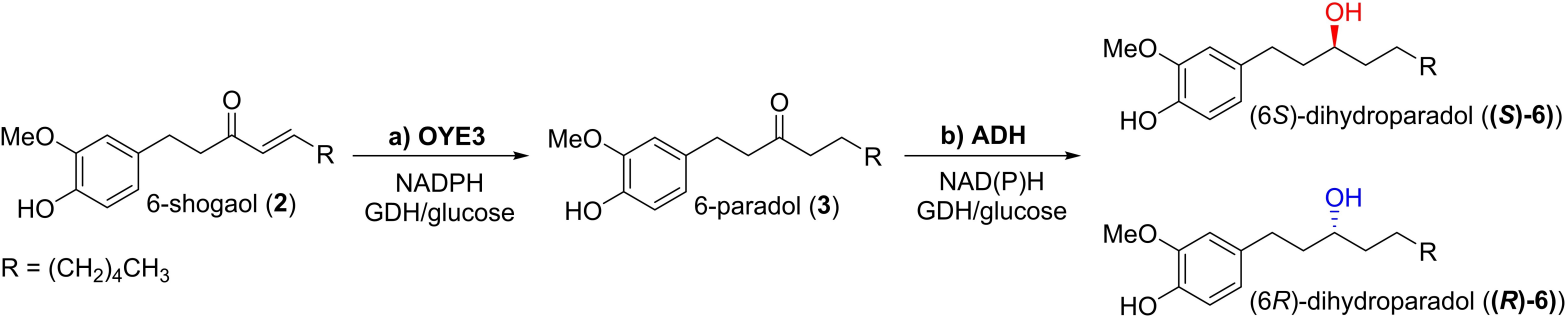
Cascade synthesis of **(*S*)‐6**/**(*R*)‐6** from **2** by coupling of the ene reductase OYE3 with selected (*R*)‐ or (*S*)‐selective ADHs.

In the light of this consideration, 6‐shogaol (**2**) was selected to build a full‐enzymatic, cascade synthesis for the preparation of 6‐dihydroparadol exploiting the activity of ERs in combination with a proper ADH aiming at obtaining both the enantiomers of the target alcohol (**(*S*)‐6** and **(*R*)‐6**, Scheme 2).

At first, the conversion of **2** to **3** was investigated on an analytical scale using different ERs from our in‐house collection.^[46]^ The screening (data not shown) identified the ene reductase OYE3 from *Saccharomyces cerevisiae* (recombinantly expressed in *E. coli*), as the best performing biocatalyst and a semi‐preparative reaction was run to isolate **3** (see Experimental Section for details).

As far as the stereoselective reduction of **3** concerns, Ml‐ADH showed a good potential, given the high conversion and de promoted on **1 a** and for its peculiar substrate scope.[^37^, ^43^]

A small‐scale reduction of the long‐chain ketone **3** (10 mg) was thus conducted in the presence of Ml‐ADH (125 μg mL^−1^, 0.5 U mL^−1^) resulting in the formation of (6*S*)‐dihydroparadol (**(*S*)‐6**) with about 83 % conversion and a modest enantiomeric excess of 78 %.

According to our previous studies on the coupling of ERs and ADHs in multienzymatic reaction systems,^[47]^ the cascade quantitative conversion of **2** to **(*S*)‐6** (10 mM) was easily achieved working at pH 7.0 and 25 °C in the presence of OYE3 (200 μg mL^−1^) and Ml‐ADH (125 μg mL^−1^, 0.5 U mL^−1^). Both needed NAD(P)H cofactor were regenerated *in situ* by the same glucose/GDH coupled system (see Supporting Information for details).

Since the exploitation of Ml‐ADH allowed the preparation of the (*S*)‐enantiomer of **6**, as well as of the (*S*,*R*)‐diastereomer of 8‐gingerdiol (**(*R*)‐5 b**), a selected panel of evoxx ADHs (Table 3), which showed good activity and selectivity on **1 a** and **4**, were tested in the stereoselective reduction of **1 b** and **3**, seeking for both the opposite absolute configuration of the synthetized stereocenters and both higher ee/de values and conversions.

**Table 3:** Screening of selected commercially available ADHs in the stereoselective reduction of **1 b** and **3**.

Entry	Enzyme^[a]^	Substrate			
		**1 b**		**3**	
		*c* [%]^[b]^	*de* [%]^[b]^	*c* [%]^[b]^	*ee* [%]^[b]^
1	evo‐1.1.010	98	>99 (*R*)	>99	96 (*S*)
2	evo‐1.1.020	93	>99 (*R*)	>99	50 (*S*)
3	evo‐1.1.250	–	–	>99	76 (*R*)
4	evo‐1.1.440	46	7 (*R*)	>99	26 (*S*)

[a] Commercially available from evoxx technologies GmbH (Monheim am Rhein, Germany). [b] Conversions and enantiomeric/diastereomeric excess values determined by chiral phase HPLC analysis after 72 h at 30 °C (Method B, see Experimental Section for details).

The obtained results are reported in Table 3. In particular, regarding the reduction of **1 b**, excellent results were obtained with both evo‐1.1.010 and evo‐1.1.020 (entries 1 and 2, respectively), with almost quantitative conversions of **1 b** into **(*R*)** *‐* **5 b** (>99 % de). Interestingly, evo‐1.1.250 (entry 3), which catalyzed the reduction of **1 a** into **(*S*)** *‐* **5 a** before, was instead not able to accept **1 b** as substrate, while it showed a very good activity toward **3** and kept the same selectivity observed with **1 a**, by forming preferentially (6*R*)‐dihydroparadol (**(*R*)‐6**) (Figure 1b). All other evoxx ADHs converted **3** quantitatively as well, but forming the **(*S*)‐6** product at different ee values, the best performing enzyme being evo‐1.1.010 (entry 1).

### Scaling up to multi mg‐scale of the biocatalyzed production of (*S*)‐7 and (*R*)‐7

**Scheme 3:**
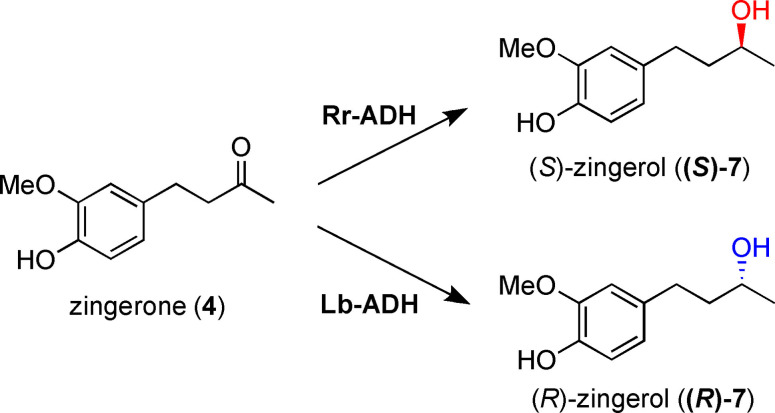
Stereoselective synthesis of **(*S*)‐7**/**(*R*)‐7** from **4** on a semi‐preparative scale (100 mg of **4**) by using Rr‐ADH or Lb‐ADH, respectively.

Concerning the semipreparative scale bioreductions, zingerone (**4**) was selected as a model substrate to run a multi mg scale production of alcohols **(*S*)‐7** and **(*R*)‐7**. The conversion of **4** into the corresponding *S* and *R* enantiomers of **7** could be in fact easily scaled‐up by exploiting two enzymes available as recombinant proteins: Rr‐ADH and Lb‐ADH, respectively (Table 2). According to the same experimental procedure described for the analytical screening, 100 mg of **4** were successfully converted into **(*S*)‐7** and **(*R*)‐7** by using these biocatalysts in combination with the *in situ* regeneration of the NAD(P)H cofactor with the glucose/GDH system (Scheme 3). After 48 h, >99 % conversions and ee values were obtained and the products could be recovered by a simple extraction with ethyl acetate, thus resulting in a quantitative isolated yield.

To build a comparison, the asymmetric reduction of the structurally simplest “challenging ketone” **4** was also carried out in the presence of Corey‐Bakshi‐Shibata (CBS)‐oxazaborolidine catalyst following literature procedures. Specifically, the experimental protocol was like that adopted by Khatua *et al*.^[48]^ employing (*R*)‐(+)‐2‐methyl‐CBS‐oxazaborolidine and borane‐dimethyl sulfide (BH_3_⋅SMe_2_) as active reductant working in methanol at −78 °C. Probably due to stereoelectronic issues which prevented the coordination complex between the boron catalyst and **4** to assume the proper transition state conformation (alkyl chain conformational freedom and lack of coordinating/directing electronegative substituents at the Cα),^[49]^ no stereoselectivity was obtained as the recovered alcohol (**8**) was a racemate (see Supporting Information for details).

This interesting example showed again the potency and convenience of the application of enzymes in the (selective) manipulation of natural products whose molecular skeletons are quite often characterized by the presence of different functional groups or challenging moieties.

## Conclusion

Exhausted vegetal residues from agri‐food processes can be a gold mine to recover bioactive molecules. In this study, an appreciable amount of oleoresin rich in gingerol‐like compounds was collected from ginger lees through traditional extraction. Based on the analytical data, the biologically active components were mostly unchanged after the fermentation process. This opens the route for new valorization opportunities of the residue.

The gingerol‐like compounds present in their structure an aliphatic ketone difficult to reduce into the corresponding chiral alcohols and diols, therefore they can be classified as challenging ketones. For the first time, we describe herein a strategy to overcome this issue using alcohol dehydrogenases which can be addressed as a convenient alternative to classical asymmetric reduction or multistep total synthesis to prepare ginger alcohol and diol derivatives as enantiomerically enriched species. The simple biocatalytic preparation of these compounds may find application in different sectors (flavor, perfume and nutraceutical), as well as permit the full disclosure of their biological activities and properties.

## Experimental Section

**Materials and chemicals**: Fermented ginger biomass was kindly provided by Prela Alba farm (Morbegno, Italy). The lees were washed with deionized water to remove the fermentation process residues, then dried in oven at 40 °C until full moisture removal. The biomass was stored in a dark flask at 20 °C until use.

The fresh ginger rhyzome was purchased by local markets (NaturaSì and Esselunga, Milan) with a declared origin from Peru or Brazil. It was dried and stored as previously described for the fermented ginger biomass.

The following commercially available alcohol dehydrogenases (ADHs) were purchased from Merck (Darmstadt, Germany): Ec‐ADH, ADH recombinant from *E. coli*, cat. n° 49854; Tb‐ADH, *Thermoanaerobium brockii* ADH, cat. n° A6184; Cb‐ADH, *Candida boidinii* ADH, cat. n° 91031; Cp‐ADH, *C. parapsilosis* ADH, cat. n° 81083; HL‐ADH, horse liver ADH, recombinant in *E. coli*, cat. n° 55689. The Alcohol Dehydrogenases Screening Kit (cat. n° evo‐1.1.100) was purchased from evoxx technologies GmbH (Monheim am Rhein, Germany).

Other reagents, HPLC‐grade and analytical grade organic solvents were purchased by Sigma‐Aldrich Chemicals (Italy) and Carlo Erba reagents s.r.l. (Italy). HPLC‐grade water was obtained from a purification system (Millipore, Billerica, MA, USA).

**General methods**: Analytical thin‐layer‐chromatography (TLC) was carried out on silica gel pre‐coated glass‐backed plates (Fluka Kieselgel 60 F254, Merck) and visualized by ultra‐violet (UV, λ=254 nm) radiation, by immersion in an ethanolic solution of sulfuric acid (10 %), then heated at 150 °C for 3 minutes. Column chromatography (CC) was performed with Silica Gel 60 (particle size 230–400 mesh, purchased from Sigma Aldrich).

Proton nuclear magnetic resonance (^1^H NMR) spectra were recorded on a Bruker Avance 400 spectrometer operating at 400.13 MHz. Proton chemical shifts (δ) are reported in ppm with the solvent reference relative to tetramethylsilane (TMS) employed as the internal standard (CDCl_3_, δ=7.26 ppm). The following abbreviations are used to describe spin multiplicity: s=singlet, d=doublet, t=triplet, q=quartet, m=multiplet, br=broad signal, dd=doublet‐doublet, td=triplet‐doublet. The coupling constant values are reported in Hz. ^13^C NMR spectra were recorded on a Bruker Avance 400 MHz spectrometer operating at 100.56 MHz, with complete proton decoupling. Carbon chemical shifts (δ) are reported in ppm relative to TMS with the respective solvent resonance as the internal standard (CDCl_3_, δ=77.23 ppm).

Optical rotation values were measured on a Jasco photoelectric polarimeter DIP 1030 using a 1 dm cell and a sodium lamp (λ=589 nm); sample concentration values (c) are given in 10^−2^ g mL^−1^.

### Analytical methods

**UPLC‐TUV analysis (Method A)**: The chromatographic analyses were performed on Waters ACQUITY UPLC system (Waters corp., MA, United States) equipped with a quaternary pump, autosampler, thermostated column compartment and a dual‐wavelength UV/Visible (UV/Vis) detector (TUV). The data were processed with Empower 3 workstations. The employed column was BEH C18 (2.1 mm×50 mm), maintained at 30 °C, and mobile phase was composed of water containing 0.1 % of formic acid (v : v) (A) and acetonitrile containing 0.1 % of formic acid (v : v) (B). The linear gradient elution used was in according to Li *et al*. with suitable modifications.^[31]^ In order to evaluate the amount of gingerols in both waste and fresh ginger, zingerone was used as reference compound to build a calibration curve (see Supporting Information). The same method was used to access the conversions and ee of the bio‐reduction of **1 b**.

**UPLC‐MS analysis**: Mass spectrometry was performed by using Thermo Scientific DIONEX ULTIMATE 3000 system (Thermo Fisher Scientific, MA, United States) equipped with a quaternary pump, autosampler, thermostated column compartment and electrospray ionization (ESI) interface source. The employed column was BEH C18 (2.1 mm×50 mm) and mobile phase was composed by water containing 0.1 % of formic acid (v : v) (A) and acetonitrile containing 0.1 % of formic acid (v : v) (B). The flow rate was set up at 0.25 mL min^−1^ and the linear gradient elution is the same of UPLC‐TUV analysis. The spectra were acquired in negative mode. The ESI source conditions were as follows: capillary voltage −6.00 V, capillary temperature 275 °C, tube lens −96 V, spray voltage 4 kV, sheath gas flow rate 40 L/h, auxiliary gas flow rate, 10 L/h. All data were acquired and processed by Xcalibur^TM^ software (Thermo Scientific, MA, United States).

**Chiral phase HPLC analysis (Method B)**: Chromatographic resolution of enantiomerically‐enriched compounds was carried out at room temperature on HPLC‐UV‐PDA Waters (Waters 2996 Photodiode Array Detector, Waters 515 HPLC pump) equipped with CHIRALCEL® OD‐H [250 mm×4.6 mm, 5 μm, produced by Daicel Industries Ltd. (Tokyo, Japan)]. The mobile phase was composed by 90 % *n*‐hexane (*n*‐Hex) and 10 % 2‐propanol (*i*‐PrOH). The flow rate was set‐up at 1 mL min^−1^ and the wavelength set at 280 nm. The data were processed with Empower 3 workstations. This method was used in combination with the zingerone calibration curve (see Supporting Information) as external standard to quantify conversion and ee of **1 b** and **2**.

Alternatively (**Method C**), chiral phase HPLC analyses were carried out on a Shimadzu LC‐20AD high performance liquid chromatography system equipped with a Shimadzu SPD‐20 A UV detector and a Phenomenex Lux 3u Cellulose‐2 chiral column (250 mm×4.6 mm). HPLC conditions: injection volume 10 μL; mobile phase: 70 % of petroleum ether and 30 % of *i*‐PrOH; flow rate: 1 mL min^−1^; detection λ: 280 nm; temperature: 30 °C. This method was used to quantify conversion and ee of **1 a** and **4** starting from the different molar absorption coefficient (ϵ) of products and starting materials at 280 nm. ϵ_**1a**_=2530 M^−1^ cm^−1^; ϵ_**5a**_=2150 M^−1^ cm^−1^; ϵ_**4**_=1815 M^−1^ cm^−1^; ϵ_**7**_=1361 M^−1^ cm^−1^.

**ESI‐HR‐MS analyses**: Mass spectrometry analyses were performed at the Mass Spectrometry facility of the Unitech COSPECT at the University of Milan (Italy) on a Q‐ToF Synapt G2‐S*i* (Waters, Milford, MA, USA). The analyses were acquired in negative mode. The ESI source conditions are the follows: capillary voltage 1.0–1.5 kV, sampling cone 30, source heater temperature 120 °C, desolvation temperature 150 °C, desolvation gas flow rate 600 L h^−1^, acquisition range 50–1200 m/z. Leucine enkephalin (Waters) was used as a lock‐mass compound. The data were processed with a MassLynx^TM^ V4.2 software (Waters).

The samples were solubilized in MS‐grade methanol (Carlo Erba Reagents, Cornaredo, Italy) and subjected for the HR‐MS analysis by direct infusion.

### General extraction procedures

**Extraction of fermented ginger biomass**: Dried fermented ginger biomass (50 g) was micronized by using a domestic coffee blender and extracted with 500 mL of dichloromethane (DCM) at room temperature (r.t.) for 6 h, under magnetic stirring. The suspension was filtered on Büchner funnel, and the organic solvent was evaporated *in vacuo* affording an amber and flavored oil (1.15 g, 2.29 %). The oil was characterized by GC‐MS, UPLC‐TUV, and UPLC‐MS techniques.

**Extraction of fresh ginger rhizome**: The procedure described before was applied on 50 g of fresh ginger rhizome affording an amber and flavored oil (3.01 g, 6.02 %). Similarly, the oil was characterized by UPLC‐TUV techniques.

**General procedure for standards preparation**: In order to isolate the natural gingerols, the oil was fractionated by column chromatography packed with silica gel and eluting firstly with *n*‐hexane‐ethyl acetate (*n*‐Hex‐EtOAc) (8 : 2, v : v) until the head fractions were recovered, then passing to *n*‐Hex‐EtOAc (7 : 3, v : v) to collect in this order: 8‐gingerol (**1 b**, 3.9 %), mixed fraction (8‐6‐gingerols, 14.3 %) and 6‐gingerol (**1 a**, 7.1 %). The organic solvent was evaporated *in vacuo* and the recovered compounds were stored at −20 °C. The identifications were in according to a literature reference.^[32]^

**General procedure for the preparation of racemic and diastereomeric standards (5 a, 5 b, 6, 7)**: The appropriate ketone (**1 a**, **1 b**, **3**, **4**) (30 mg, 1 eq) was dissolved in MeOH (3 mL). Then, sodium borohydride (NaBH_4_) (1.6 eq for compounds **1 a**,**1 b**, **3**, and 3.44 eq for **4**) was added gradually, under stirring for 30 min in nitrogen atmosphere (N_2_). The reaction was monitored by TLC (*n‐*Hex‐EtOAc, 6 : 4, v : v) until the complete conversion of the starting material into the corresponding reduced product. Then, the reaction was quenched with a saturated solution of NH_4_Cl (2.5 mL) and extracted with DCM (5×2.5 mL). The organic layers were collected, dried over Na_2_SO_4_ and the organic solvent evaporated under reduced pressure to afford the alcohol (**5 a**, **5 b**, **6**, **7**) as an oil. The final racemic/diastereomeric compound was stored at −20 °C without further purification. The identifications complied to literature reference.^[32]^

**Preparation of compound 2**: 18.5 % of aqueous HCl (1.5 mL) was added dropwise to **1 a** (100 mg) under magnetic stirring. The mixture was gently warmed (65–70 °C) for 3 h, then it was neutralized with a saturated solution of ammonium chloride (NH_4_Cl) and extracted with DCM (5×10 mL). The organic layers were combined, dried over anhydrous sodium sulfate (Na_2_SO_4_) and concentrated *in vacuo*. The pure product was isolated after purification on silica gel column chromatography (*n*‐Hex‐EtOAc, 8 : 2, v : v) as amber oil (55 % yield). The characterization of the obtained compound was in according to literature references.[^22^, ^50^]

**Biocatalysts preparation**: Expression and purification of HSDHs/SDRs (see Table S1 in Supporting Information) and *Bacilllus megaterium* glucose dehydrogenase (GDH) were carried out as previously described.^[38]^ The ene reductase OYE3 from *Saccharomyces cerevisiae* was recombinantly expressed in *E. coli* as previously reported.^[47]^

Genes coding for ADH from *Lactobacillus kefir* (Lk‐ADH),^[35]^ ADH from *Lactobacillus brevis* (Lb‐ADH),^[36]^ ADH−A from *Rhodococcus ruber* DSM 44541 (Rr‐ADH),^[34]^ and ADH from *Micrococcus luteus* (Ml‐ADH)^[37]^ were codon optimized for expression in *E. coli*, synthetized and cloned in the pET28a vector (Lk‐ADH, Lb‐ADH, Rr‐ADH) or pET24a (Ml‐ADH) by Twist bioscience (San Francisco, CA, USA). Recombinant genes expression was carried out in *E. coli* BL21(DE3) cells with slight modification of literature methods (see Supporting Information for details).

### Biotransformations

**Screening of ADHs, HSDHs and SDRs in the stereoselective reduction of 1 a–b and 4**: If not stated otherwise, enzyme screening was performed on analytical scale (2–5 mg of substrate, final volume 1 mL) under the following experimental conditions. Substrates (10 mM) were dissolved in a 5 % v : v solution of DMSO in 50 mM phosphate buffer (PB), pH 7.0. The tested ADH, HSDH or SDR (1 mg mL^−1^, if not stated otherwise), glucose (40 mM), NAD(P)^+^ cofactor (0.2 mM), and GDH (0.5 U mL^−1^) were added to the obtained solution. Reactions were then incubated at 30 °C for 72 h, extracted with 0.5 mL of EtOAc, dried over Na_2_SO_4_, and concentrated *in vacuo*. For the specific details for each biocatalyst used, see Table S1 of Supporting information.

Screening reactions carried out with the Alcohol Dehydrogenases Screening Kit from evoxx technologies GmbH were conducted following the protocol given by the manufacturer.

**Semi‐preparative, stereoselective reduction of 4**: A zingerone (**4**) solution (100 mg, 10 mM) was prepared in 5 % v : v mixture of DMSO in 50 mM PB, pH 7.0 (volume=50 mL). Glucose (40 mM), NAD(P)^+^ cofactor (0.2 mM) and GDH (0.5 U mL^−1^) and the proper ADH (Rr‐ADH or Lb‐ADH to produce **(*S*)‐7** or **(*R*)‐7**, respectively) were added and the reaction was incubated in a thermoshaker at 100 rpm and 30 °C in the case of Rr‐ADH or 25 °C for Lb‐ADH. The reactions were monitored by TLC (*n*‐Hex‐EtOAc, 6 : 4, v : v, UV+Komarovsky reagent^[51]^) attesting, after 24 h of incubation, the complete conversion of the starting ketone. Target alcohols **(*S*)‐7** and **(*R*)‐7** were isolated as transparent oils (quantitative yield) after extraction with EtOAc, anhydrification over Na_2_SO_4_ and *in vacuo* concentration.

**Semi‐preparative, OYE3‐mediated reduction of 2 to 3**: **2** (24 mg, 10 mM in final volume 8.6 mL) was suspended in a 20 % v : v solution of DMSO in 50 mM PB, pH 7.0, to which OYE3 (200 μg mL^−1^), GDH (0.5 U mL^−1^), NADP^+^ cofactor (0.1 mM), and glucose (40 mM) were subsequently added. The obtained mixture was incubated in a thermoshaker at 25 °C and 180 rpm and followed by TLC analysis (*n‐*Hex‐EtOAc, 8 : 2, v : v, UV+Komarovsky reagent). After 72 h the reaction, uncompleted, was extracted with EtOAc, dried over Na_2_SO_4_, and concentrated *in vacuo* recovering unreacted **2** and the product **3** which was purified through flash column chromatography on silica gel (*n‐*Hex‐EtOAc, 8 : 2, v : v; 18 % yield).

**Enzymatic synthesis of (*S*)‐6 from 3**: **3** (9.3 mg, 0.10 mmol) was dissolved (25 mM) in 5 % v : v solution of DMSO in 2.3 mL of 50 mM PB pH 7.0. To this solution, glucose (40 mM), NAD^+^ cofactor (0.2 mM), GDH (0.5 U mL^−1^) and Ml‐ADH (125 μg mL^−1^, 0.5 U mL^−1^) were added. The obtained mixture was incubated at 25 °C and monitored by TLC analysis (*n*‐Hex/EtOAc, 8 : 2, v : v). After 72 h, the reaction was extracted three times with DCM and the combined organic layers were washed twice with brine, dried over Na_2_SO_4_ and concentrated *in vacuo*. The crude product was purified by chromatography gravity column (*n‐*Hex‐EtOAc, 6 : 4, v : v), to afford **(*S*)‐6** as a pale‐yellow oil (28 %).

**OYE3‐Ml‐ADH cascade for the enzymatic synthesis of (*S*)‐6**: **2** (10 mM) was suspended in a 20 % v : v solution of DMSO in 50 mM PB, pH 7.0, to which OYE3 (200 μg mL^−1^), Ml‐ADH (125 μg mL^−1^, 0.5 U mL^−1^), GDH (0.5 U mL^−1^), NAD^+^ (0.2 mM), NADP^+^ (0.2 mM) and glucose (40 mM) were subsequently added. The obtained mixture was incubated in a thermomixer at 25 °C and 500 rpm. After 24 h, the complete conversion of **2** into **(*S*)‐6** was attested by TLC (*n‐*Hex‐EtOAc, 6 : 4, v : v, UV+Komarovsky reagent). The reaction was then extracted with EtOAc, dried over Na_2_SO_4_, and concentrated *in vacuo* affording the target alcohol.

### Characterization of isolated and synthesized compounds

(*S*)*‐5‐Hydroxy‐1‐*(*4‐hydroxy‐3‐methoxyphenyl*)*decan‐3‐one* (**1 a**): light yellow oil (7.17 %). R_f_: 0.46 (*n*‐Hex‐EtOAc, 6 : 4, v : v). ${\left[\alpha \right]}_{D}^{25}=$ +20.1 (c 1, CHCl_3_). ^1^H NMR (400 MHz, CDCl_3_) δ 6.81 (d, *J*=8.0 Hz, 1H), 6.70–6.62 (m, 2H), 5.73 (s, 1H), 4.03 (d, *J*=4.1 Hz, 1H), 3.86 (s, 3H), 3.08 (s, 1H), 2.83 (t, *J*=7.0 Hz, 2H), 2.73 (t, *J*=7.0 Hz, 2H), 2.53 (qd, *J*=17.3, 5.7 Hz, 2H), 1.27 (s, 8H), 0.88 (t, *J*=6.4 Hz, 3H). ^13^C NMR (101 MHz, CDCl_3_) δ 211.5, 146.6, 144.0, 132.6, 120.7, 114.4, 111.4, 67.7, 55.7, 49.3, 45.36, 36.5, 31.7, 29.2, 25.1, 22.5, 13.8.^[32]^ R_t_: 7.33 min (Method A). [M−H]^+^ (C_17_ H_25_ O_4_): Calculated: m/z=293.1753, Found: m/z=293.1751.

(*S*)*‐5‐Hydroxy‐1‐*(*4‐hydroxy‐3‐methoxyphenyl*)*tetradecan‐3‐one* (**1 b**): light yellow oil (3.93 %), R_f_: 0.50 (*n*‐Hex‐EtOAc, 6 : 4, v : v), ${\left[\alpha \right]}_{D}^{25}=$ +22.6 (c 0.52, CHCl_3_).^1^H NMR (400 MHz, CDCl_3_) δ 6.82 (t, *J*=7.1 Hz, 1H), 6.76–6.58 (m, 2H), 5.60 (s, 1H), 4.08–3.97 (m, 1H), 3.87 (s, 3H), 2.90–2.80 (m, 2H), 2.78–2.67 (m, 2H), 2.60–2.43 (m, 2H), 2.05 (s, 1H), 1.27 (s, 10H), 0.89 (t, *J*=6.4 Hz, 3H). ^13^C NMR (101 MHz, CDCl_3_) δ 211.5, 146.5, 144.0, 132.6, 120.7, 114.4, 111.0, 67.7, 60.4, 55.9, 49.3, 45.4, 36.5, 31.9, 29.5, 29.3, 25.4, 22.7, 14.1.^[52]^ R_t_: 9.92 min (Method A). [M−H]^+^ (C_19_ H_29_ O_4_): Calculated: m/z=321.2066, Found: m/z=321.2065.

(*E*)*‐1‐*(*4‐Hydroxy‐3‐methoxyphenyl*)*dec‐4‐en‐3‐one* (**2**): light yellow oil, (65 %), R_f_: 0.63 (*n*‐Hex‐EtOAc, 7 : 3, v : v), ^1^H NMR (400 MHz, CDCl_3_) δ 6.88–6.77 (m, 2H), 6.69 (dd, *J*=14.9, 8.3 Hz, 2H), 6.10 (d, *J*=15.9 Hz, 1H), 5.58 (s, 1H), 3.87 (s, 3H), 2.90–2.71 (m, 4H), 2.20 (q, *J*=6.8 Hz, 2H), 1.27 (t, *J*=12.1 Hz, 7H), 0.89 (d, *J*=6.8 Hz, 3H). ^13^C NMR (101 MHz, CDCl_3_) δ 199.9, 147.9, 146.4, 143.9, 133.2, 130.3, 120.8, 114.3, 111.1, 55.9, 42.0, 32.4, 31.3, 29.9, 27.7, 22.4, 13.9.^[32]^ R_t_: 9.22 min (Method A). [M−H]^+^ (C_17_ H_23_ O_3_): Calculated: m/z=275.1647, Found: m/z=275.1644.

*1‐*(*4‐Hydroxy‐3‐methoxyphenyl*)*decan‐3‐one* (**3**): yellow oil (96.1 %) R_f_: 0.35 (*n*‐Hex‐EtOAc, 8 : 2, v : v). ^1^H NMR (400 MHz, CDCl_3_) δ 6.88–6.79 (m, 1H), 6.69 (d, *J*=7.4 Hz, 2H), 5.49 (s, 1H), 3.89 (s, 3H), 2.84 (d, *J*=6.7 Hz, 2H), 2.70 (d, *J*=6.0 Hz, 2H), 2.39 (s, 2H), 1.57 (d, *J*=12.4 Hz, 2H), 1.27 (s, 8H), 0.89 (d, *J*=5.8 Hz, 3H).^[22] 13^C NMR (101 MHz, CDCl_3_) δ 210.6, 146.2, 143.8, 133.1, 120.7, 114.3, 111.0, 55.8, 44.6, 43.1, 31.6, 29.5, 29.1, 29.0, 23.8, 22.6, 14.0. R_t_: 9.54 min (Method A). [M−H]^+^ (C_17_ H_25_ O_3_): Calculated: m/z =277.1804, Found: m/z=277.1808.

(*3S,5S*)*‐1‐*(*4‐Hydroxy‐3‐methoxyphenyl*)*decane‐3,5‐diol* (**(*S*)‐5 a**) *and* (*3R,5S*)*‐1‐*(*4‐hydroxy‐3‐methoxyphenyl*)*decane‐3,5‐diol* (**(*R*)‐5 a**) *mixture*: pale yellow oil (96.3 %). R_f_: 0.17, 0.10 (*n*‐Hex‐EtOAc, 6 : 4, v : v). ^1^H NMR (400 MHz, CDCl_3_) δ 6.85 (d, *J*=7.9 Hz, 1H), 6.75–6.67 (m, 2H), 3.99 (m, 1H), 3.89 (s, 3H), 3.76 (m, 1H), 2.81–2.54 (m, 2H), 1.96–1.69 (m, 2H), 1.56 (d, *J*=9.9 Hz, 1H), 1.46 (m, 2H), 1.29 (d, *J*=9.1 Hz, 8H), 0.91 (s, 3H). ^13^C NMR (101 MHz, CDCl_3_) δ 146.4, 143.8, 133.9, 120.9, 114.3, 111.0, 73.3, 72.3 (**(*S*)‐5 a**), 69.5, 68.9 (**(*R*)‐5 a**), 60.4, 55.8, 42.9, 42.4, 40.0, 39.4, 38.3, 37.5, 31.9, 31.8, 31.4, 25.4, 25.0, 22.6, 14.0. R_t_ **(*R*)‐5 a**): 7.05 min R_t_ **(*S*)‐5 a**: 6.85 min, (Method A). R_t_ **(*R*)‐5 a**: 5.72 min; R_t_ **(*S*)‐5 a**: 5.10 min (Method C). [M−H]^+^ (C_17_ H_27_ O_4_): Calculated: m/z=295.1909, Found: m/z=295.1910.

(*3R,5S*)*‐1‐*(*4‐Hydroxy‐3‐methoxyphenyl*)*decane‐3,5‐diol* (**(*R*)‐5 a**): light yellow oil (12.9 %), R_f_: 0.17 (*n*‐Hex‐EtOAc, 6 : 4, v : v); *de* >99 %, ${\left[\alpha \right]}_{D}^{20}$_=_ (+) 10.8 (c 0.187, CHCl_3_). ^1^H NMR (400 MHz, CDCl_3_) δ 6.76 (d, *J*=7.9 Hz, 1H), 6.67–6.59 (m, 2H), 3.90–3.83 (m, 1H), 3.81 (s, 3H), 3.79–3.74 (m, 1H), 3.68 (s, 2H), 2.75–2.48 (m, 2H), 1.77–1.63 (m, 2H), 1.58–1.33 (m, 4H), 1.23–1.15 (m, 6H), 0.82 (t, *J*=6.7 Hz, 3H). ^13^C NMR (101 MHz, CDCl_3_) δ 146.4, 143.7, 133.7, 132.0, 120.9, 114.3, 111.0, 73.7 (*R,S*), 72.7 (*R,S*), 55.8, 42.4, 39.8, 38.1, 31.7, 31.3, 24.9, 22.6, 14.0.^[18]^ R_t_: 7.05 min (Method A). [M−H]^+^ (C_17_ H_27_ O_4_): Calculated: m/z=295.1909, Found: m/z=295.1909.

(*3S, 5S*)*‐1‐*(*4‐Hydroxy‐3‐methoxyphenyl*)*decane‐3,5‐diol* (**(*S*)‐5 a**): light yellow oil (46.7 %), R_f_: 0.10 (*n*‐Hex‐EtOAc, 6 : 4, v : v); *de* >99 %, ${\left[\alpha \right]}_{D}^{20}$ =(−) 9.79 (c 0.22, CHCl_3_). ^1^H NMR (400 MHz, CDCl_3_) δ 6.85 (d, *J*=7.9 Hz, 1H), 6.76–6.70 (dd, *J*=13.8, 8.3 Hz, 2H), 3.94 (s, 1H), 3.90 (s, 3H), 3.78 (s, 2H), 2.81–2.57 (m, 2H), 1.87–1.71 (m, 2H), 1.63 (dd, *J*=23, 12 Hz, 1H), 1.54–1.46 (m, 1H), 1.38–1.23 (m, 5H), 0.91 (t, *J*=6.7 Hz, 8H). ^13^C NMR (101 MHz, CDCl_3_) δ 146.4, 143.7, 133.9, 120.8, 114.3, 111.0, 69.5, 68.9, 55.9, 42.4, 39.4, 37.5, 32.0, 31.8, 25.4, 22.6, 14.0.^[18]^ R_t_: 6.85 min (Method A). [M−H]^+^ (C_17_ H_27_ O_4_): Calculated: m/z=295.1909, Found: m/z=295.1908.

(*3S,5S*)*‐1‐*(*4‐Hydroxy‐3‐methoxyphenyl*)*dodecane‐3,5‐diol* (**(*S*)‐5 b**) *and* (*3R,5S*)*‐1‐*(*4‐hydroxy‐3‐methoxyphenyl*)*dodecane‐3,5‐diol* (**(*R*)‐5 b**): pale yellow oil (95 %). R_f_ **(*R*)‐5 b**: 0.23 0; R_f_ **(*S*)‐5 b**: 0.48 (*n*‐Hex‐EtOAc, 6 : 4, v : v) ^1^H NMR (400 MHz, CDCl_3_) δ 7.08 (s, 1H), 6.85 (d, *J*=7.9 Hz, 1H), 6.71 (d, *J*=11.3 Hz, 2H), 5.48 (s, 1H), 4.05–3.94 (m, 1H), 3.89 (s, 3H), 3.88 (s, 1H), 2.82–2.58 (m, 2H), 1.78 (t, *J*=20.6 Hz, 2H), 1.60 (dd, *J*=29.9, 12.2 Hz, 4H), 1.28 (s, 12H), 0.89 (t, *J*=6.4 Hz, 3H). ). ^13^C NMR (101 MHz, CDCl_3_) δ 146.3, 143.7, 133.7, 121.0, 114.2, 111.1, 72.7, 71. 69.8, 68.9, 55.9, 39.1, 38.5, 37.2, 36.5, 35.9, 31.9, 30.9, 29.5, 29.3, 25.4, 24.97, 22.7, 14.1. R_t_ **(*R*)‐5 b**: 9.51 min. R_t_ **(*S*)‐5 b**: 9.77 min (Method A). [M−H]^+^(C_19_ H_31_ O_4_): Calculated: m/z=323.2222, Found: m/z=323.2219.

*4‐*(*3‐Hydroxydecyl*)*‐2‐methoxyphenol* (**6**): light yellow oil (96 %). R_f_: 0.41 (*n*‐Hex‐EtOAc, 6 : 4, v : v). ^1^H NMR (400 MHz, CDCl_3_) δ 6.77 (d, *J*=7.6 Hz, 1H), 6.64 (s, 2H), 5.39 (d, *J*=2.5 Hz, 1H), 3.90 (s, 1H), 3.81 (s, 3H), 2.70–2.61 (m, 1H), 2.54 (td, *J*=9.9, 4.9 Hz, 1H), 1.80–1.55 (m, 4H), 1.41–1.34 (m, 1H), 1.26–1.22 (m, 3H), 1.18 (s, 6H), 0.82 (d, *J*=5.0 Hz, 4H). . ^13^C NMR (101 MHz, CDCl_3_) δ 146.4, 143.7, 134.12, 120.9, 114.2, 111.0, 71.5, 55.9, 39.4, 37.6, 31.8, 29.7, 29.6, 29.2, 25.6, 22.6, 14.1.^[53]^ R_t_: 9.13 min (Method A); *R_t1_*: 13.08 min_,_ *R_t2_*: 17.82 min (Method B). [M−H]^+^ (C_17_ H_27_ O_3_): Calculated: m/z=279.1960, Found: m/z=279.1959.

(*S*)*‐4‐*(*3‐Hydroxydecyl*)*‐2‐methoxyphenol* (**(*S*)‐6**): pale‐yellow oil (27.7 %). R_f_: 0.41 (*n*‐Hex‐EtOAc, 6 : 4, v : v), ${\left[\alpha \right]}_{D}^{20}$_=_ +7.3 (c 0.13, CHCl_3_). ^1^H NMR (400 MHz, CDCl_3_) δ 6.85 (d, *J*=7.7 Hz, 1H), 6.72 (d, *J*=8.1 Hz, 2H), 3.90 (s, 3H), 3.65 (dt, *J*=10.7, 5.2 Hz, 1H), 2.80–2.70 (m, 1H), 2.68–2.58 (m, 1H), 1.74 (dd, *J*=15.2, 7.9 Hz, 2H), 1.54–1.43 (m, 4H), 1.28 (s, 8H), 0.90 (d, *J*=6.9 Hz, 3H). ^13^C NMR (101 MHz, CDCl_3_) δ 146.4, 143.7, 134.12, 120.9, 114.2, 111.0, 71.5, 55.9, 39.4, 37.6, 31.8, 29.7, 29.6, 29.2, 25.6, 22.6, 14.1.^[53]^ R_t_: 17.22 min (Method B). [M−H]^+^ (C_17_ H_27_ O_3_): Calculated: m/z=279.1960, Found: m/z=279.1959.

*4‐*(*3‐Hydroxybutyl*)*‐2‐methoxyphenol* (**7**): Colorless oil (91.1 %). R_f_: 0.27 (*n*‐Hex‐EtOAc, 6 : 4, v : v). ^1^H NMR (400 MHz, CDCl_3_)=δ 6.83 (d, *J*=8.0 Hz, 1H), 6.73–6.56 (m, 2H), 5.50 (s, 1H), 3.87 (s, 3H), 2.90–2.78 (m, 2H), 2.78–2.61 (m, 2H), 2.14 (s, 3H). ^13^C NMR (101 MHz, CDCl_3_)=δ 146.6, 143.7, 134.0, 120.9, 114.5, 111.3, 67.5, 55.9, 41.1, 31.8, 23.5. R_t_: 4.567 min (Method A); *R_t1_*: 17.44 min_,_ *R_t2_*: 20.94 min (Method B). R_t_: **(*S*)‐7**): 5.59 min; R_t_ (**(*R*)‐7**): 6.29 min (Method C).^[24]^ [M−H]^+^ (C_11_ H_15_ O_3_): Calculated: m/z=195.1021, Found: m/z=195.1020.

(*S*)*‐4‐*(*3‐Hydroxybutyl*)*‐2‐methoxyphenol* (**(*S*)‐7**): Colorless oil (90.3 %). R_f_:0.27 (*n*‐Hex‐EtOAc, 6 : 4, v : v), *ee*=99.9 %, ${\left[\alpha \right]}_{D}^{20}$_=_ +11.25 (c 0.37, CHCl_3_). ^1^H NMR (400 MHz, CDCl_3_) δ 6.84 (d, *J*=7.8 Hz, 1H), 6.71 (d, *J*=8.8 Hz, 2H), 5.57 (s, 1H), 3.89 (s, 3H), 3.84 (dt, *J*=12.3, 6.3 Hz, 1H), 2.75–2.64 (m, 1H), 2.63–2.57 (m, 1H), 1.80–1.70 (m, 2H), 1.24 (d, *J*=6.2 Hz, 3H). ^13^C NMR (101 MHz, CDCl_3_) δ 146.4, 143.7, 133.9, 120.92, 114.2, 110.9, 67.6, 55.9, 41.1, 31.8, 23.7.^[24]^ R_t_: 4.49 min (Method A), R_t_: 20.945 min (Method B), R_t_: 5.59 min (Method C). [M−H]^+^ (C_11_ H_15_ O_3_): Calculated: m/z=195.1021, Found: m/z=195.1019.

(*R*)*‐*(−)*‐4‐*(*3‐Hydroxybutyl*)*‐2‐methoxyphenol* (**(*R*)‐7**): Colorless oil (98.5 %). R_f_: 0.27 (*n*‐Hex‐EtOAc, 6 : 4, v : v), *ee*=98,5 %, ${\left[\alpha \right]}_{D}^{20}$ =−14.06 (c 0.398, CHCl_3_). ^1^H NMR (400 MHz, CDCl_3_) δ 6.84 (d, *J*=7.8 Hz, 1H), 6.71 (d, *J*=9.4 Hz, 2H), 5.61 (s, 1H), 3.88 (s, 3H), 3.83 (dd, *J*=12.1, 6.1 Hz, 1H), 2.74–2.57 (m, 2H), 1.80–1.72 (m, 2H), 1.24 (d, *J*=6.1 Hz, 3H). ^13^C NMR (101 MHz, CDCl_3_) δ 146.4, 143.6, 134.0, 120.9, 114.3, 110.94, 67.6, 55.9, 41.1, 31.8, 23.7.^[24]^ R_t_: 4.492 min (Method A), R_t_: 17.44 min (Method B), R_t_: 5.59 min (Method C). [M−H]^+^ (C_11_ H_15_ O_3_): Calculated: m/z=195.1021, Found: m/z=195.1020.

## Conflict of interest

The authors declare no conflict of interest.

1

## Supporting information

As a service to our authors and readers, this journal provides supporting information supplied by the authors. Such materials are peer reviewed and may be re‐organized for online delivery, but are not copy‐edited or typeset. Technical support issues arising from supporting information (other than missing files) should be addressed to the authors.

Supporting Information

## Data Availability

The data that support the findings of this study are available in the supplementary material of this article.
