# Supplementary material for: Stereoselective Biocatalyzed Reductions of Ginger Active Components Recovered from Industrial Wastes
Source: Chembiochem. 2022 Mar 3;23(8):e202200105. doi: 10.1002/cbic.202200105 (PMC9314113; doi:10.1002/cbic.202200105)
Supplement: Supplementary file 1 — Supporting Information [file CBIC-23-0-s001.pdf]

ChemBioChem

Supporting Information

Stereoselective Biocatalyzed Reductions of Ginger Active Components Recovered from Industrial Wastes

Rita Nasti,* Ivan Bassanini,* Erica Elisa Ferrandi, Federica Linguardo, Susanna Bertuletti, Marta Vanoni, Sergio Riva, Luisella Verotta,* and Daniela Monti*

Table of contents

1. Table S1. In-house collection of ADH/HSDH/SDR enzymes used in this work.
2. Recombinant production of Lk-ADH, Lb-ADH, Ml-ADH and Rr-ADH
3. Zingerone calibration curve
4. Chiral phase HPLC chromatograms of ADH-catalyzed reduction of **4** to **(*S*)-7** and **(*R*)-7**
5. Chiral phase HPLC chromatograms of ADH-catalyzed reduction of **1a** to **(*R*)-5a** and **(*S*)-5a**
6. UPLC-UV chromatograms of ADH-catalyzed reduction of **1b** to **(*R*)-5b** and **(*S*)-5b**
7. UPLC-UV (Method A) and chiral phase HPLC chromatograms (Method B) of enzymatic reduction of compound **3** to **(*S*)-6** and **(*R*)-6**
8. Characterization of compounds **1a**, **1b**, **2, 3**, **4**, **(*R*)-5a**, **(*S*)-5a**, **(*R*)-5b**, **(*S*)-5b**, **6**, **(*S*)-6**, **7**, **(*S*)-7, (*R*)-7** (^{1}H-NMR, ^{13}C-NMR, UPLC profiles)
9. Asymmetric reduction of **4** *via* (*R*)-(+)-2-methyl-CBS-oxazaborolidine catalyst

1) Table S1. In-house collection of ADH/HSDH/SDR enzymes used in this work.

Enzyme	Source	Cofactor	Accession n° UniProtKB/Swiss-Prot or GenBank	Reference
Ml-ADH	*Micrococcus luteus*	NAD^+	ADD83022.1	[1]
Lk-ADH	*Lactobacillus kefir*	$NADP^+$	AAP94029.1	[2]
Rr-ADH	*Rhodococcus ruber*	NAD^+	Q8KLT9	[3]
Lb-ADH	*Lactobacillus brevis*	$NADP^+$	Q84EX5	[4]
Is2-SDR	Metagenome sample	$NADP^+$	MN733985	[5]
Ca7α-HSDH	*Clostridium absonum*	$NADP^+$	AET80685.1	[6]
Dm7α-HSDH	*Deinococcus marmoris*	NAD^+	WP_075832535.1	[5]
Ec7α-HSDH	*Escherichia coli*	NAD^+	KXH01569.1	[7]
Hh7α-HSDH	*Halomonas halodenitrificans*	NAD^+	WP_027961750.1	[5]
Ngi1_7α-HSDH	Metagenome sample	NAD^+	WP_011911126.1	[5]
Bsp7ß-HSDH	*Brucella* sp.	NAD^+	WP_004684107.1	[5]
Ca7ß-HSDH	*Clostridium absonum*	$NADP^+$	AET80684.1	[6]
Cae7ß-HSDH	Collinsella aerofaciens	$NADP^+$	WP_006236005.1	[8]
Hh7ß-HSDH	*Halomonas halodenitrificans*	NAD^+	WP_027961749.1	[5]
Rs7ß-HSDH	*Rhodobacter sphaeroides*	NAD^+	WP_015212061.1	[5]
Sc7ß-HSDH	*Stanieria cyanosphaera*	NAD^+	WP_006236005.1	[5]
Csp12α-HSDH	*Clostridium* sp.	$NADP^+$	AOB10242.1	[9]
Ls12α-HSDH	*Lysinibacillus sphaericus*	NAD^+	WP_036222017.1	[5]

2) Recombinant production of Lk-ADH, Lb-ADH, Ml-ADH and Rr-ADH

Production of Lk-ADH and Lb-ADH. *E. coli* BL21(DE3) carrying pET28_Lk-ADH or pET28_Lb-ADH from glycerol stocks were plated on LB-Agar plates containing 30 µg mL^{-1} kanamycin (LB_{kan30}) and incubated overnight at 37°C. The following day some colonies were removed from the plate by using an inoculating loop and inoculated in 100 mL of LB_{kan30}. The cell culture was grown overnight at 37°C and 220 rpm. 50 mL of these pre-cultures were used for the inoculation of 1 L of LB_{kan30}, and cells were incubated at 37°C and 220 rpm till OD_{600} reached a value between 0.5 – 0.8. Protein expression was then induced using 1 mM IPTG final concentration and after induction cell incubation temperature was shifted to 30°C. After 24 h, cells were harvested by centrifugation and cell pellet was resuspended in 20 mL wash buffer (500 mM NaCl, 20 mM imidazole, 20 mM potassium phosphate (PB) buffer, pH 7.0). Cells were lysed by sonication at 0 °C (30 sec-on/15 sec-off x 6) and cell lysate was centrifuged at 10000 rpm and 4 °C for 30 min. Cell extract was incubated for 2 h with Ni-NTA resin to purify the target proteins. The suspension was finally loaded into a glass column, washed with 10 mL of the washing buffer and target protein elution was performed by applying buffers containing increasing concentrations of imidazole (100 – 300 mM)
The protein concentration of the eluates was determined by Bradford assay. The eluates containing the target protein were collected, transferred into dialysis tubes and dialyzed against 20 mM PB, pH 7.0. Protein purity was checked by SDS-PAGE.

Production of Ml-ADH
Ml-ADH was first produced as described for Lk-ADH and Lb-ADH, with the only difference being cell incubation at 30°C for 5 h after induction. Due to the very low protein yield obtained with this protocol, Ml-ADH production was optimized using the autoinduction medium ZYM-5052[10] as culture medium instead of LB broth.
Colonies of *E. coli* BL21(DE3) carrying pET24_Ml-ADH were inoculated in 100 mL of LB_{kan30} and grown overnight at 37°C and 220 rpm. The following day a suitable amount of overnight culture was added to 500 mL of ZYM-5052_{kan30} to obtain a final OD_{600} of 0.2. The cell culture was then incubated at 20°C for 48 h. Cell lysis and protein purification was carried out as described before. The purified protein was dialyzed against 20 mM PB, pH 8.0. 12 mg of pure Ml-ADH were obtained from 500 mL culture. Ml-ADH activity was spectrophotometrically measured following reduction of $NADP^+$ at 340 nm (ε: 6.22 mM^{-1} cm^{-1}) using 4-decanol as substrate. Specifically, in a polyethylene cuvette at room temperature 10-50 µl of Ml-ADH were added to an assay solution containing 2.5 mM 4-decanol and 0.2 mM $NADP^+$ in 50 mM PB, pH 8.0. One unit of activity is defined as the enzyme amount that reduces 1 µmol of $NADP^+$ per min under the assay conditions described above. 47 U of Ml-ADH were obtained from 500 ml culture.

Expression of Rr-ADH (ADH-A)
Colonies of *E. coli* BL21(DE3) carrying pET28_Rr-ADH, grown overnight on LB_{kan30} agar plate, were inoculated in 500 mL of LB_{kan30} medium containing 100 mg L^{-1} $ZnCl_2$ and grown at 30°C overnight. The following day, OD_{600} was monitored till it reached 3.5. Protein expression was then induced with 2 mM IPTG (final concentration) and cells were further incubated at 20°C for 24 h. Cell culture was centrifuged at 8000 rpm for 20 min at 4°C, the supernatant was removed and cell pellet was resuspended in water and lyophilized.

3) Zingerone calibration curve

To evaluate the amount of gingerols in both waste and fresh ginger, zingerone was used as reference compound to build a calibration curve.

Commercially available standard of zingerone (Sigma Aldrich, Milan, Italy) was dissolved in acetonitrile. A calibration curve was built using the method of external standard by means of a seven - points calibration curve with three replicate measurements for each calibration point and the data were processed through Microsoft Excel software. The curve was linear in a range of 530-0.106 μg mL^{-1} with a calculated correlation coefficient calculated coefficient value (R^2) of 0.9998 (Figure S1). Limit of detection (LOD) and limit of quantification (LOQ) were calculated as 3- and 10-fold the signal to noise ratio with a value of 8.68 ng mL^{-1} and 28.95 ng mL^{-1} respectively.

The chromatographic analyses were performed on Waters ACQUITY UPLC system (Waters corp., MA, United States) equipped with a quaternary pump, autosampler, thermostated column compartment and a dual-wavelength UV/Visible (UV/Vis) detector (TUV). The data were processed with Empower 3 workstations. The employed column was BEH C18 (2.1 mm x 50 mm), maintained at 30°C, and mobile phase was composed of water containing 0.1% of formic acid (v/v) (A) and acetonitrile containing 0.1% of formic acid (v/v) (B). The linear gradient elution used was in according to Li Y. et al.[11] with suitable modifications.

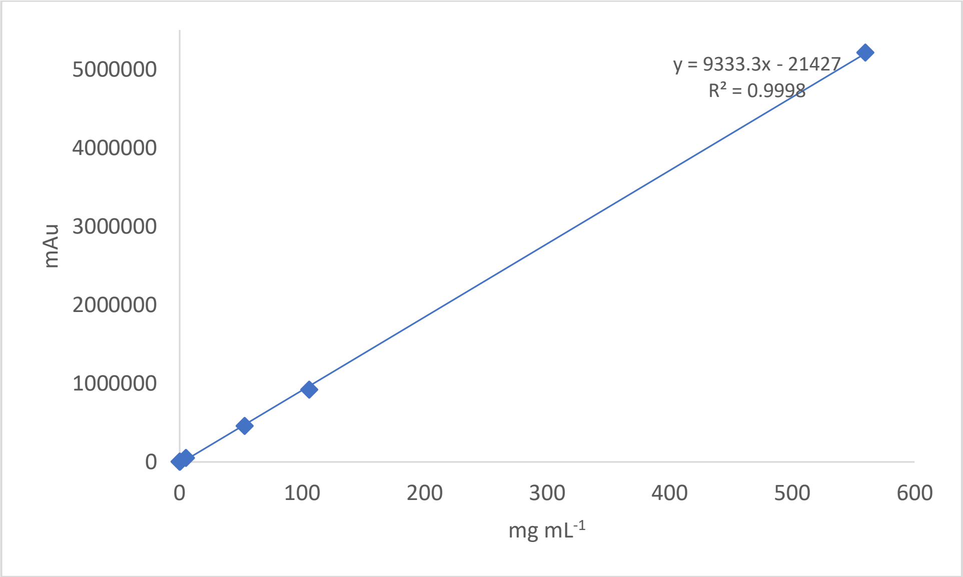

Figure S1. Zingerone (**1**) calibration curve

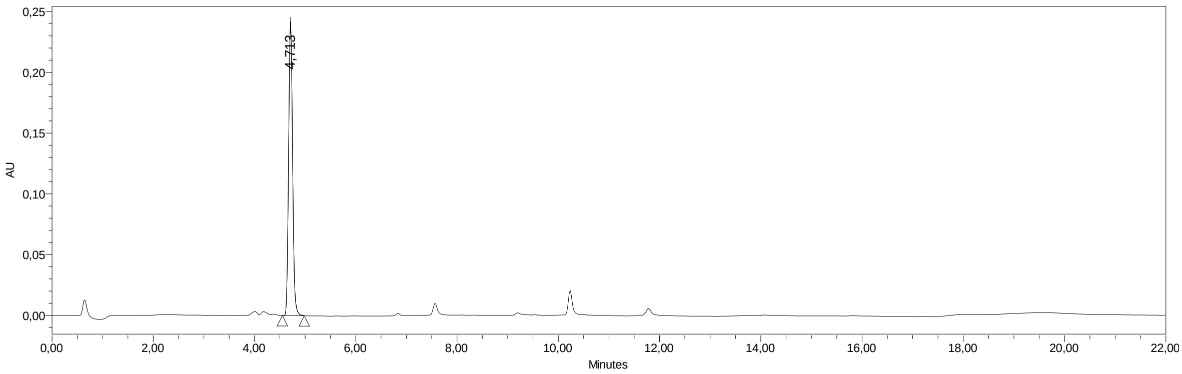

Figure S2. Chromatographic profile of zingerone (**1**) by UPLC-TUV method; λ: 280 nm, 0.25 mL min^{-1}.

4) Chiral phase HPLC chromatograms of ADH-catalysed reduction of 4 to (*S*)-7 and (*R*)-7

Analysis Report

Chiral phase HPLC analyses were carried out on a Shimadzu LC-20AD high performance liquid chromatography system equipped with a Shimadzu SPD-20A UV detector and a Phenomenex Lux 3u Cellulose-2 chiral column (250 mm x 4.6 mm). HPLC conditions: injection volume 10 µL; mobile phase: 70% of petroleum ether and 30% of *i*-PrOH; flow rate: 1 mL min^{-1}; detection λ: 280 nm; temperature: 30°C.

<Sample Information>

Sample Name	: ZOH + ZONE		
Sample ID	: ZOH + ZONE		
Data Filename	: ZOH + ZONE.lcd		
Method Filename	: Gradiente flusso Zingerone.lcm		
Batch Filename	:		
Vial #	: 1-1	Sample Type	: Unknown
Injection Volume	: 10 uL		
Date Acquired	: 09/03/2021 11:41:47	Acquired by	: COMPUTER
Date Processed	: 09/03/2021 12:11:48	Processed by	: COMPUTER

<u>**Standards**</u>**, Rt:** (*R*)-Zingerol (**(*R*)-7**) 5.5 min; (*S*)-Zingerol (**(*S*)-7**) 6.2 min; Zingerone (**4**) 14.7 min

<Chromatogram>

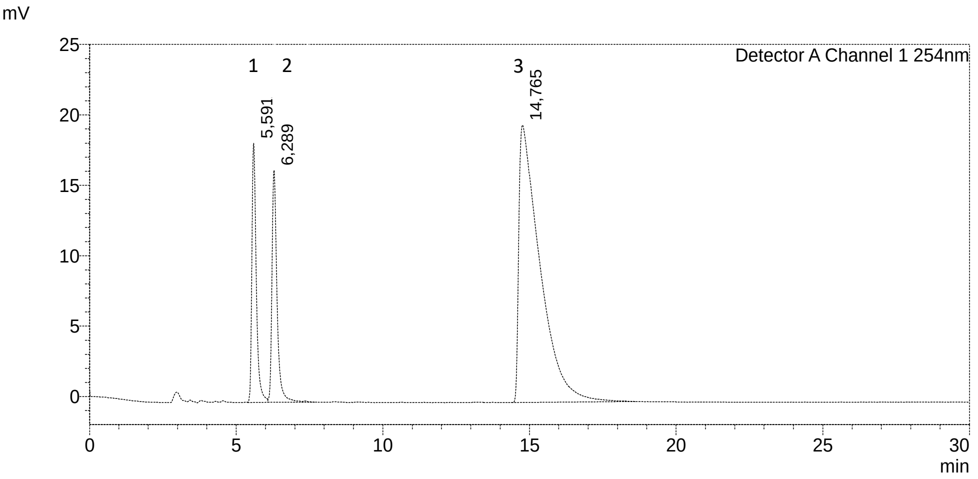

<Peak Table>

Detector A Channel 1 254nm

Peak#	Ret. Time	Area
1	5,591	193320
2	6,289	199102
3	14,765	985001
Total		1377423

Reported chromatograms are named after the ADH employed as biocatalyst and selected on the basis of detectable conversions and ees.

Ec-ADH

<Chromatogram>

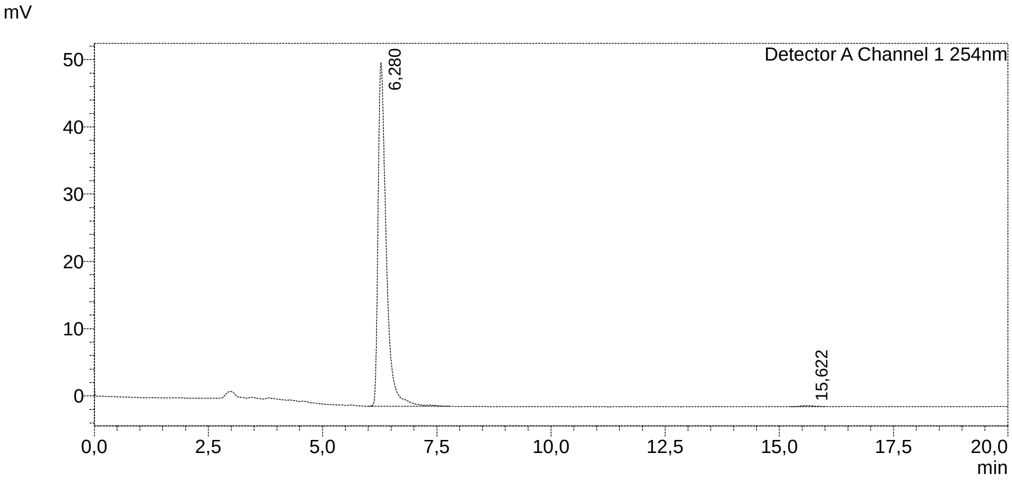

<Peak Table>

Detector A Channel 1 254nm

Peak#	Ret. Time	Area
1	6,280	633713
2	15,622	3747
Total		637460

Tb-ADH

<Chromatogram>

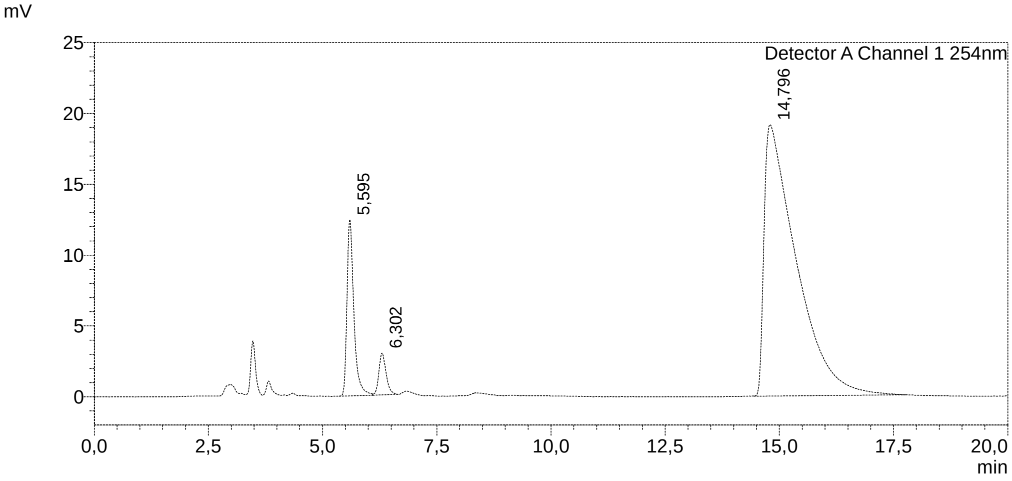

<Peak Table>

Detector A Channel 1 254nm

Peak#	Ret. Time	Area
1	5,595	130973
2	6,302	32325
3	14,796	940440
Total		1103737

Hl-ADH

<Sample Information>

Sample Name	: FL45 end		
Sample ID	: FL45 end		
Data Filename	: FL45 end.lcd		
Method Filename	: Protocollo reazione Zingerone.lcm		
Batch Filename	:		
Vial #	: 1-1	Sample Type	: Unknown
Injection Volume	: 10 uL		
Date Acquired	: 07/04/2021 17:25:34	Acquired by	: COMPUTER
Date Processed	: 07/04/2021 17:45:35	Processed by	: COMPUTER

<Chromatogram>

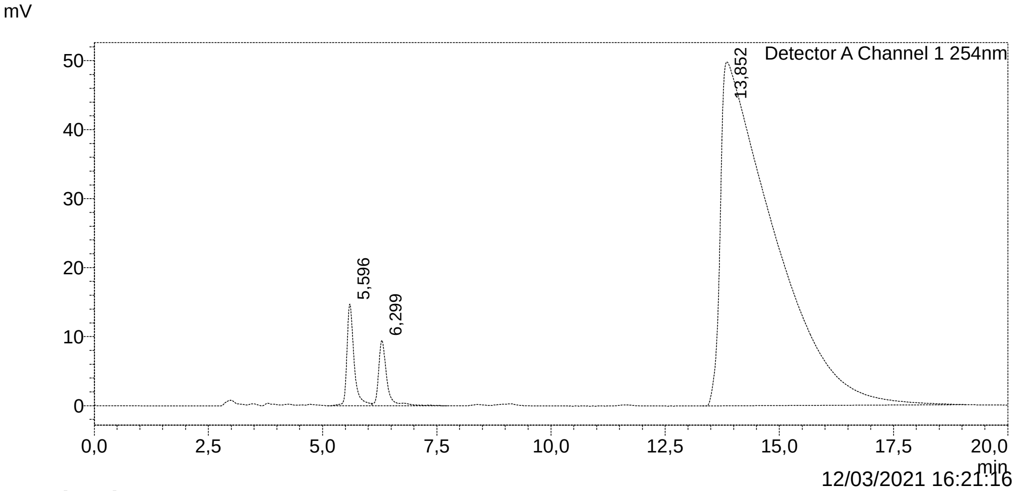

12/03/2021 16:21:16 Page 1 / 1

<Peak Table>

Detector A Channel 1 254nm

Peak#	Ret. Time	Area
1	5,596	160680
2	6,299	117204
3	13,852	4059466
Total		4337349

Analysis Report

Ml-ADH

<Sample Information>

Sample Name	: FL6 4d 25°C		
Sample ID	: FL6 4d 25°C		
Data Filename	: FL6 4d 25°C.lcd		
Method Filename	: Gradiente flusso Zingerone.lcm		
Batch Filename	:		
Vial #	: 1-1	Sample Type	: Unknown
Injection Volume	: 10 uL		
Date Acquired	: 10/03/2021 13:51:02	Acquired by	: COMPUTER
Date Processed	: 10/03/2021 14:11:05	Processed by	: COMPUTER

<Chromatogram>

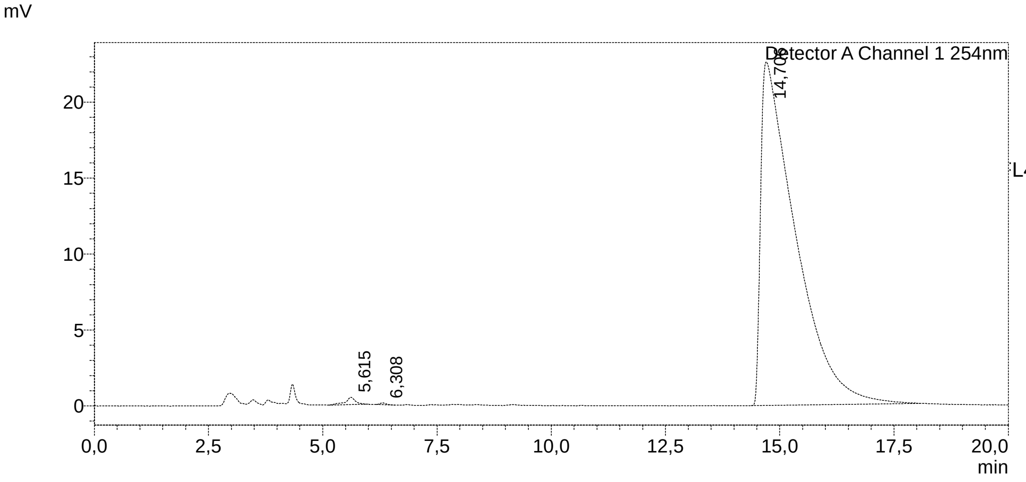

L45 end.lcd

<Peak Table>

Detector A Channel 1 254nm

Peak#	Ret. Time	Area
1	5,615	7499
2	6,308	1249
3	14,706	1200810
Total		1209559

Lk-ADH

<Chromatogram>

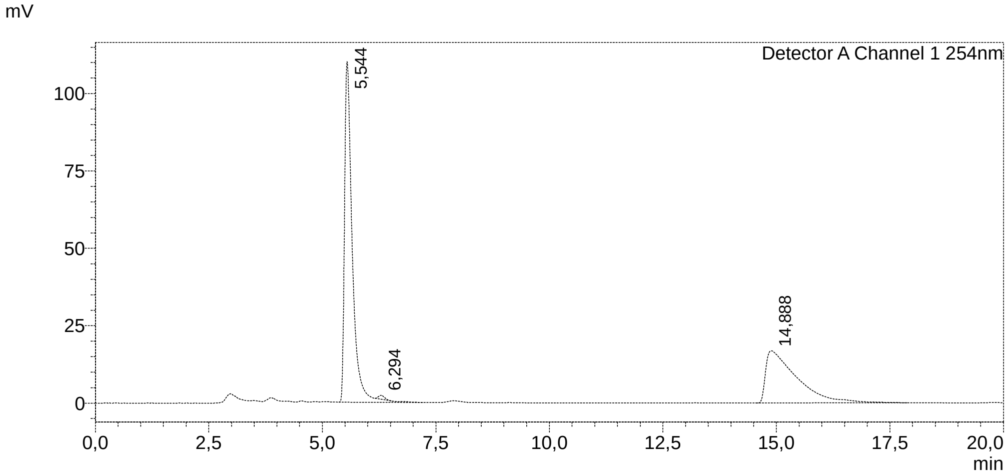

<Peak Table>

Detector A Channel 1 254nm

Peak#	Ret. Time	Area
1	5,544	1309349
2	6,294	12070
3	14,888	803688
Total		2125107

Rr-ADH

<Chromatogram>

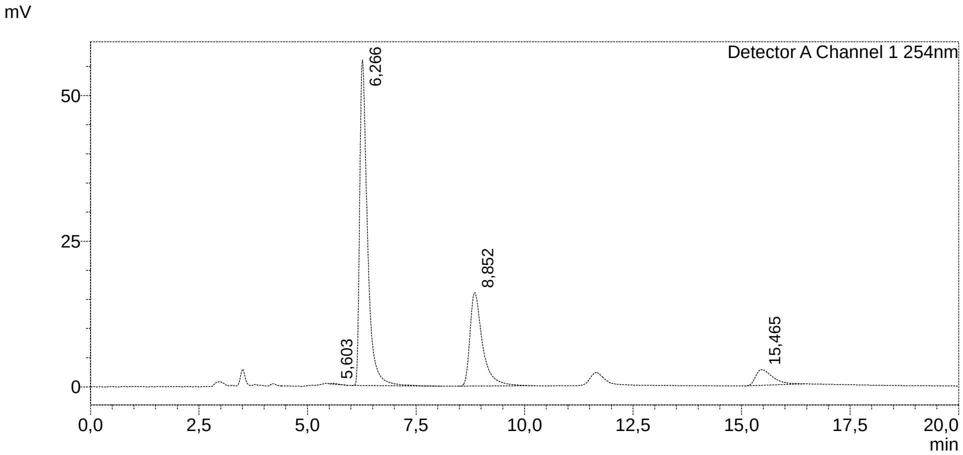

<Peak Table>

Detector A Channel 1 254nm

Peak#	Ret. Time	Area
1	5,603	1151
2	6,266	699522
3	8,852	325429
4	15,465	75054
Total		1101156

Lb-ADH

<Chromatogram>

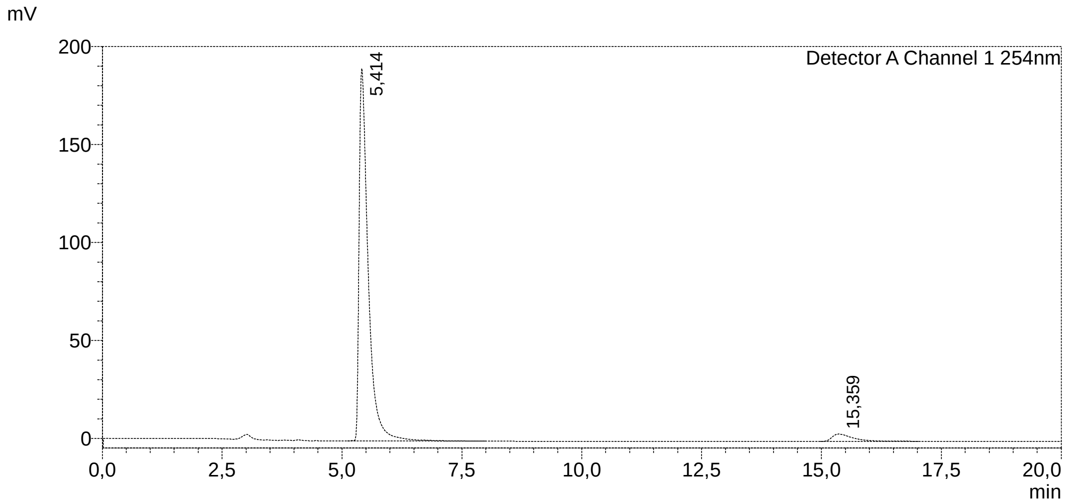

<Peak Table>

Detector A Channel 1 254nm

Peak#	Ret. Time	Area
1	5,414	2499749
2	15,359	113793
Total		2613543

Cp-ADH

<Chromatogram>

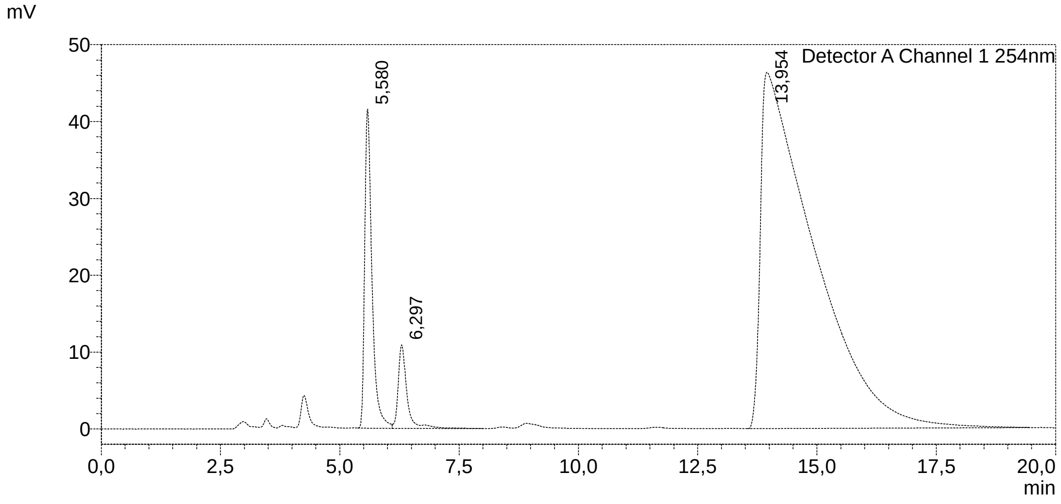

<Peak Table>

Detector A Channel 1 254nm

Peak#	Ret. Time	Area
1	5,580	444646
2	6,297	138561
3	13,954	3634365
Total		4217572

IS2-SDR

<Chromatogram>

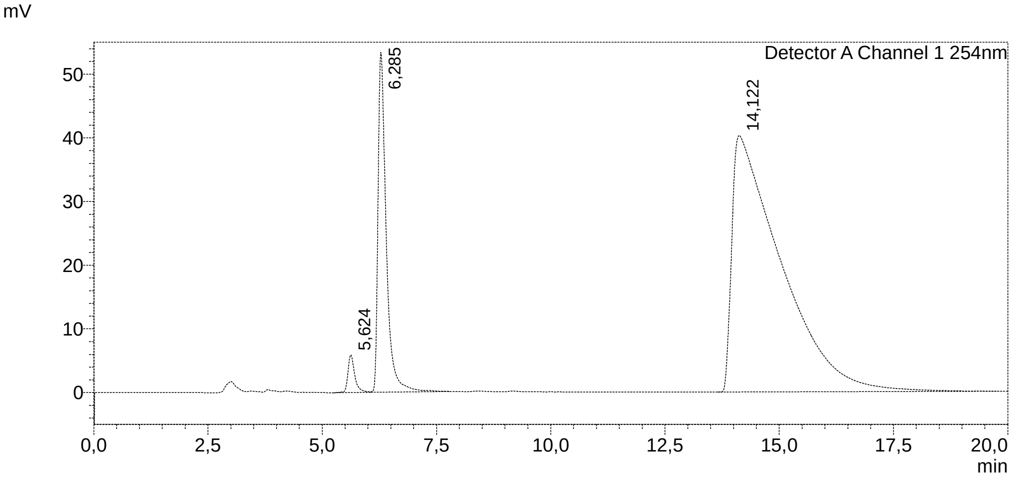

<Peak Table>

Detector A Channel 1 254nm

Peak#	Ret. Time	Area	Name
1	5,624	61782	
2	6,285	664580	
3	14,122	3016118	
Total		3742480	

evo-1.1.010

<Chromatogram>

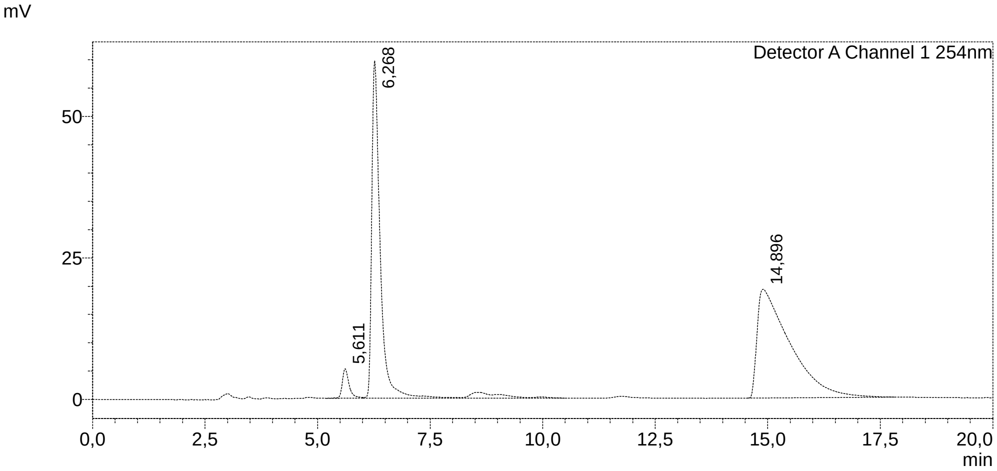

<Peak Table>

Detector A Channel 1 254nm

Peak#	Ret. Time	Area
1	5,611	55118
2	6,268	819587
3	14,896	980885
Total		1855590

evo-1.1.020

<Chromatogram>

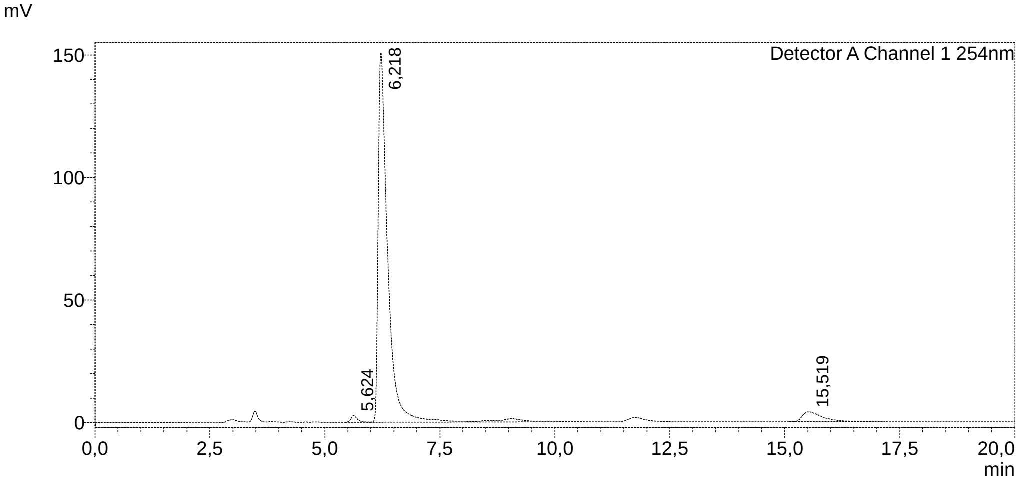

<Peak Table>

Detector A Channel 1 254nm

Peak#	Ret. Time	Area
1	5,624	28109
2	6,218	2230240
3	15,519	123961
Total		2382310

evo-1.1.030

<Chromatogram>

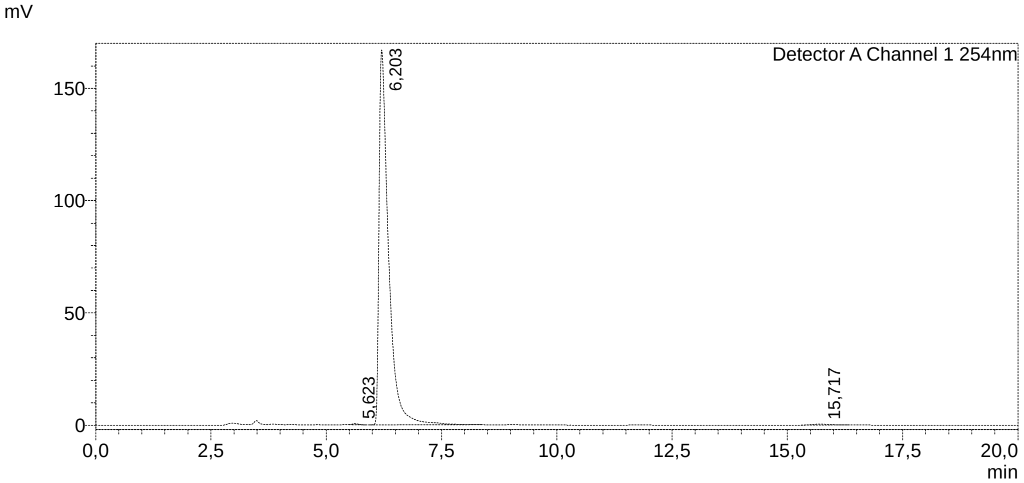

<Peak Table>

Detector A Channel 1 254nm

Peak#	Ret. Time	Area
1	5,623	4490
2	6,203	2403974
3	15,717	10199
Total		2418663

evo-1.1.040

<Chromatogram>

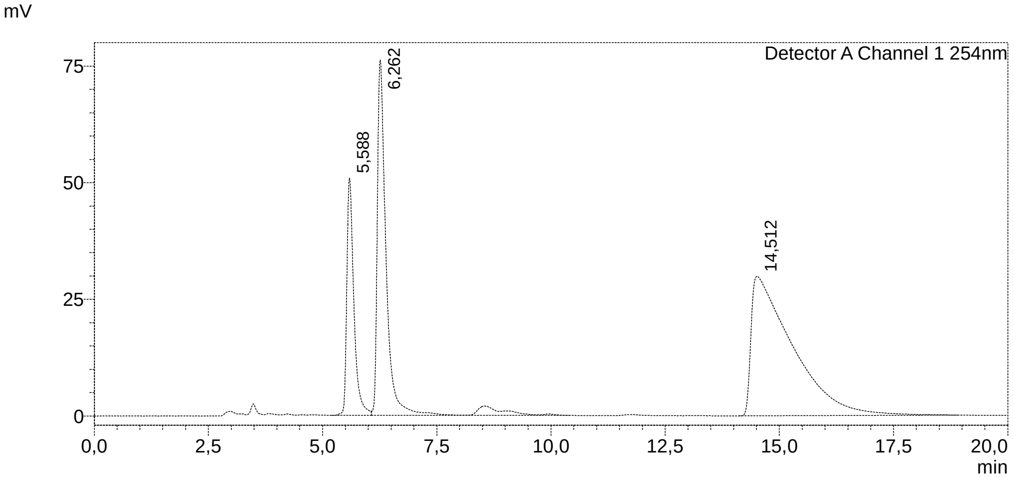

<Peak Table>

Detector A Channel 1 254nm

Peak#	Ret. Time	Area
1	5,588	551712
2	6,262	1089918
3	14,512	1888344
Total		3529973

evo-1.1.130

<Chromatogram>

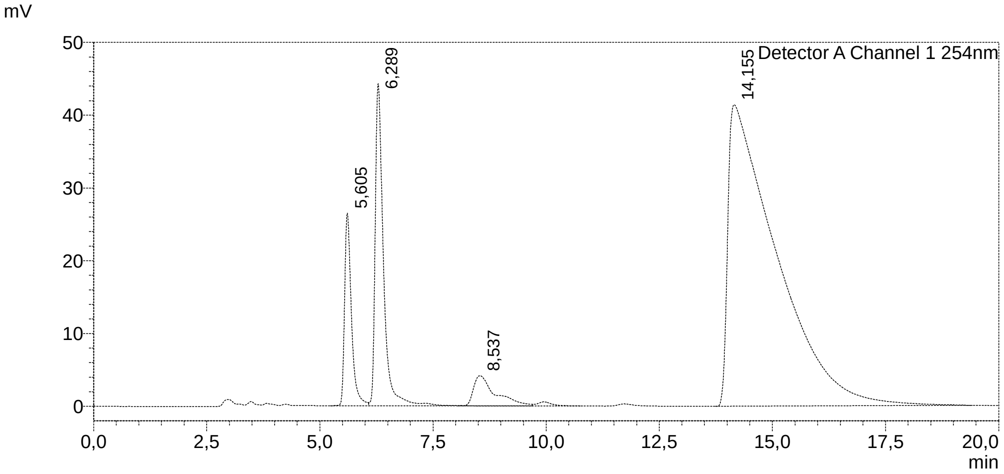

<Peak Table>

Detector A Channel 1 254nm

Peak#	Ret. Time	Area
1	5,605	280082
2	6,289	565023
3	8,537	136254
4	14,155	3108466
Total		4089825

evo-1.1.140

<Chromatogram>

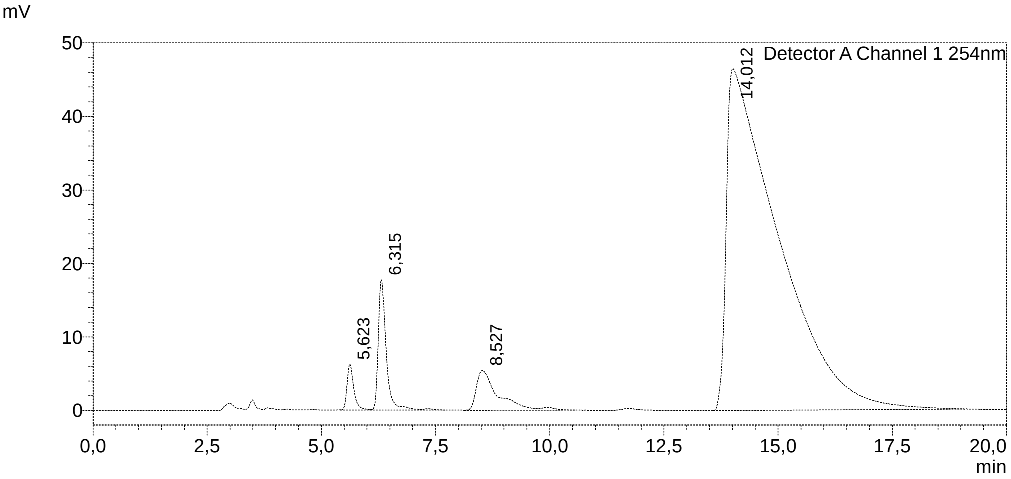

<Peak Table>

Detector A Channel 1 254nm

Peak#	Ret. Time	Area
1	5,623	65293
2	6,315	215836
3	8,527	179940
4	14,012	3699452
Total		4160521

evo-1.1.190

<Chromatogram>

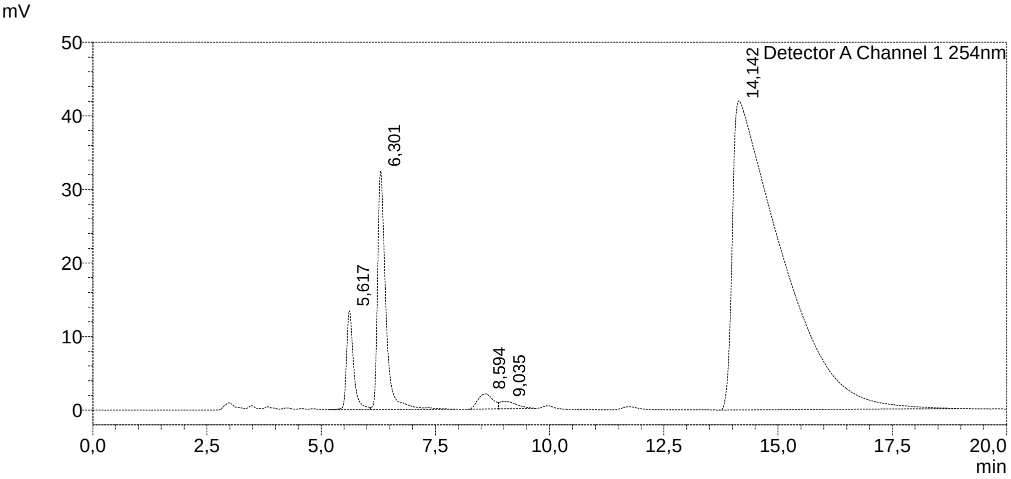

<Peak Table>

Detector A Channel 1 254nm

Peak#	Ret. Time	Area
1	5,617	143263
2	6,301	402828
3	8,594	48476
4	9,035	25638
5	14,142	3165326
Total		3785531

evo-1.1.200

<Chromatogram>

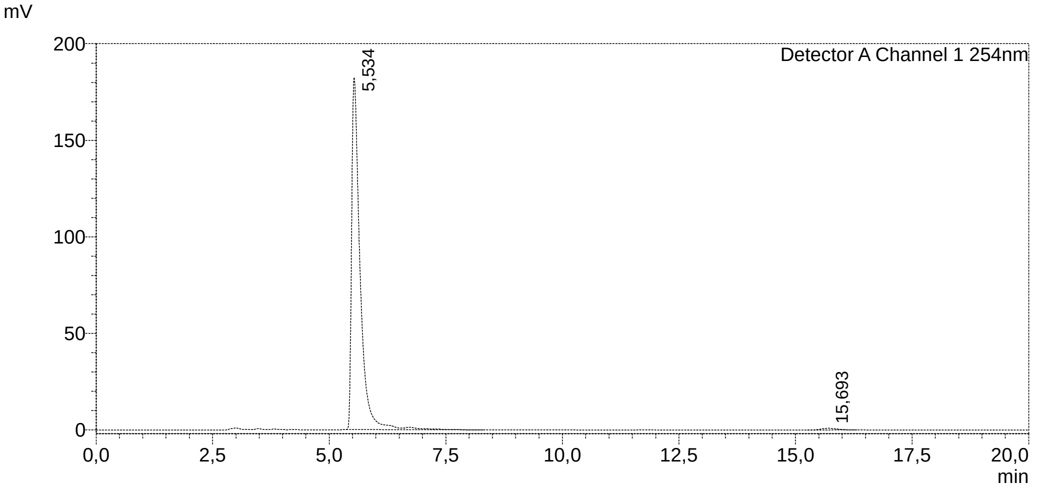

<Peak Table>

Detector A Channel 1 254nm

Peak#	Ret. Time	Area
1	5,534	2312224
2	15,693	21000
Total		2333225

evo-1.1.210

<Chromatogram>

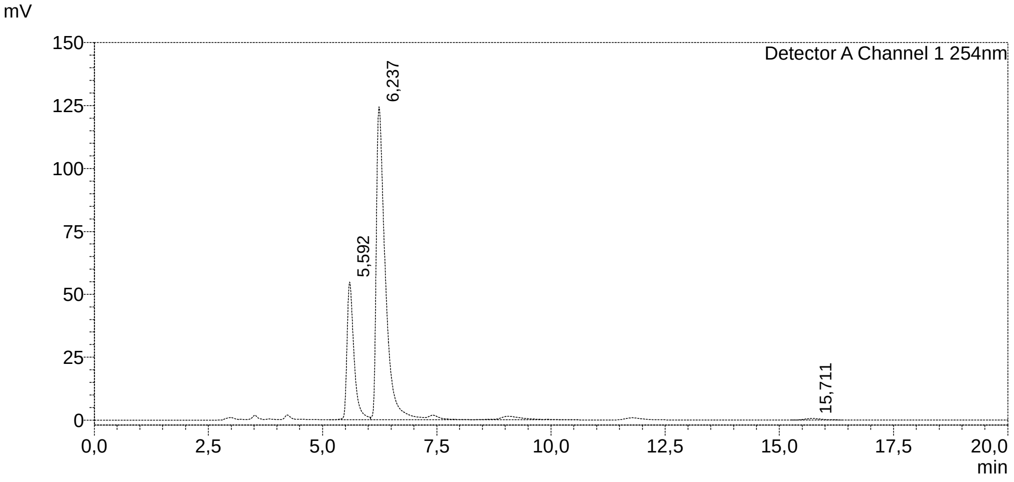

<Peak Table>

Detector A Channel 1 254nm

Peak#	Ret. Time	Area
1	5,592	586075
2	6,237	1805995
3	15,711	15339
Total		2407409

evo-1.1.250

<Chromatogram>

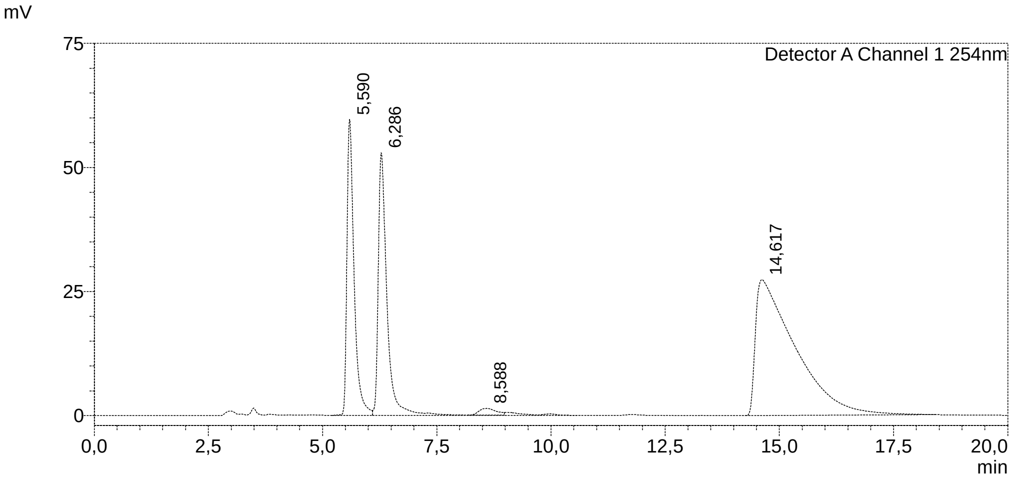

<Peak Table>

Detector A Channel 1 254nm

Peak#	Ret. Time	Area
1	5,590	652972
2	6,286	698685
3	8,588	36458
4	14,617	1660038
Total		3048153

evo-1.1.260

<Chromatogram>

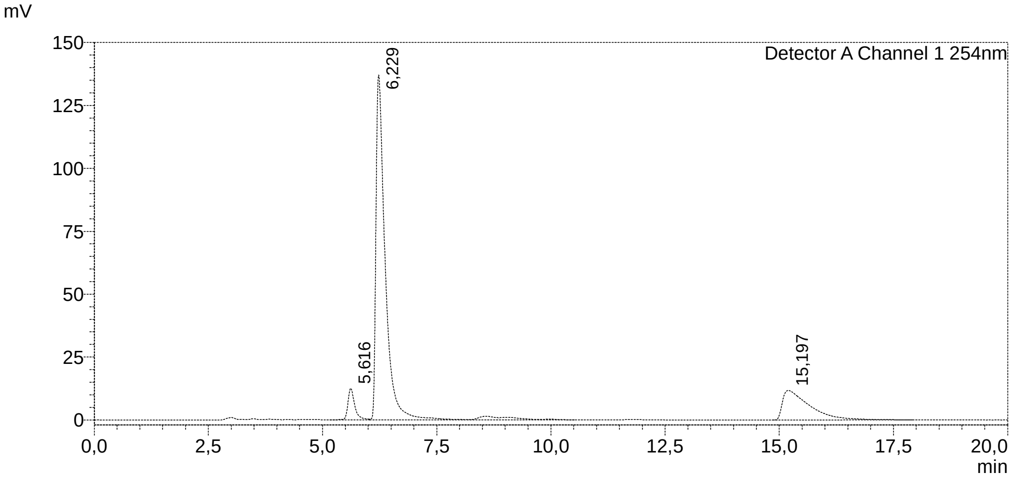

<Peak Table>

Detector A Channel 1 254nm

Peak#	Ret. Time	Area
1	5,616	130914
2	6,229	1997565
3	15,197	488367
Total		2616847

evo-1.1.270

<Chromatogram>

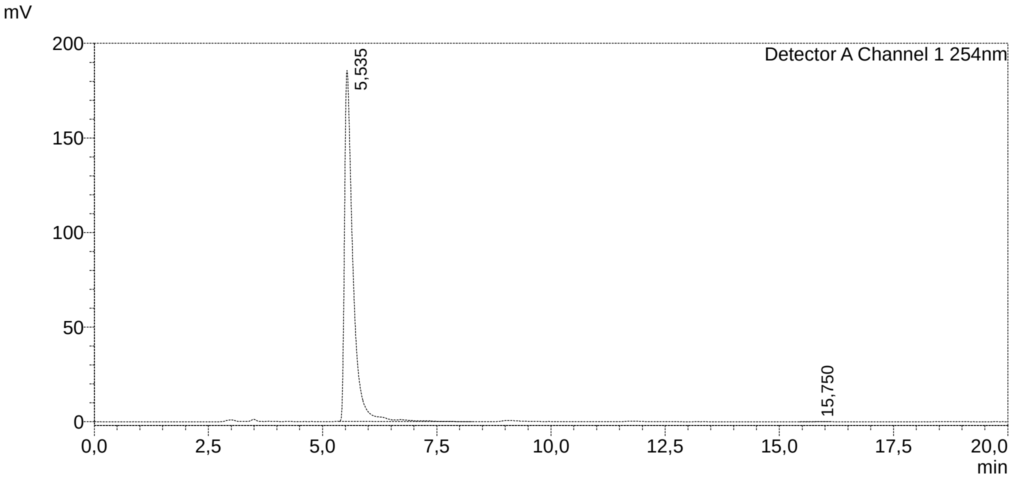

<Peak Table>

Detector A Channel 1 254nm

Peak#	Ret. Time	Area
1	5,535	2370772
2	15,750	2848
Total		2373620

evo-1.1.380

<Chromatogram>

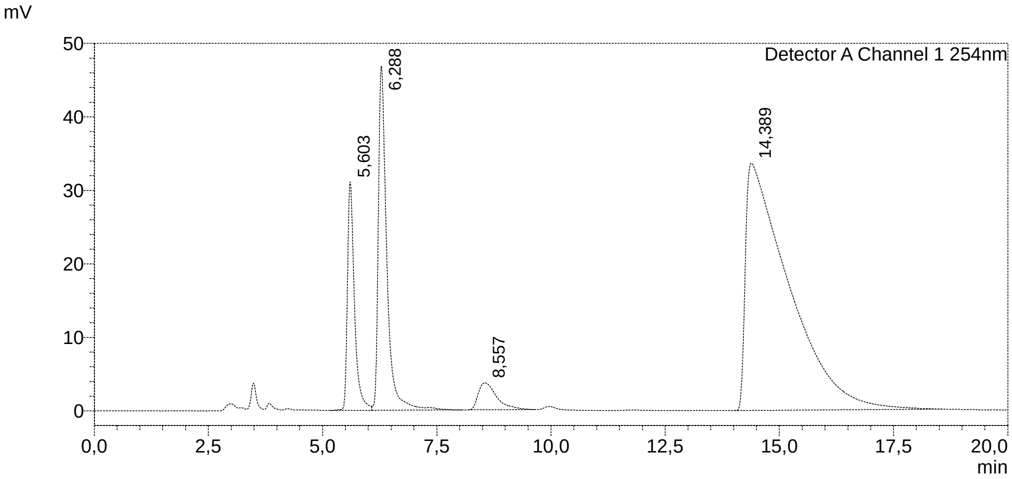

<Peak Table>

Detector A Channel 1 254nm

Peak#	Ret. Time	Area
1	5,603	334801
2	6,288	600894
3	8,557	103370
4	14,389	2241468
Total		3280533

evo-1.1.420

<Chromatogram>

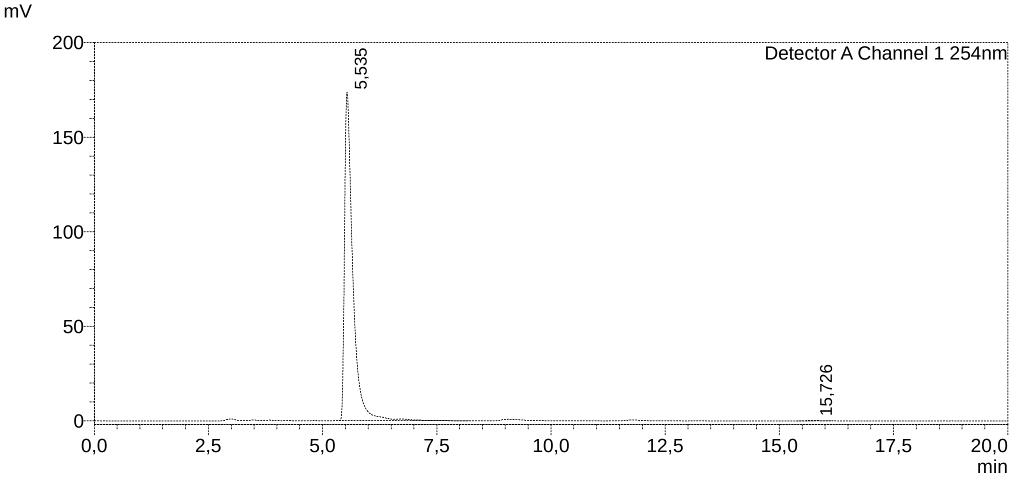

<Peak Table>

Detector A Channel 1 254nm

Peak#	Ret. Time	Area
1	5,535	2195871
2	15,726	4666
Total		2200537

evo-1.1.430

<Chromatogram>

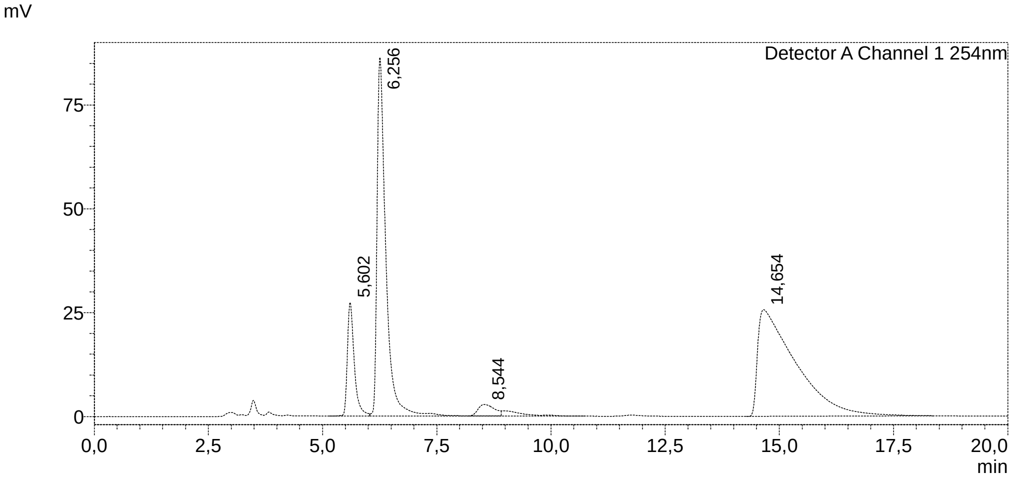

<Peak Table>

Detector A Channel 1 254nm

Peak#	Ret. Time	Area
1	5,602	290786
2	6,256	1164313
3	8,544	69114
4	14,654	1495009
Total		3019223

evo-1.1.440

<Chromatogram>

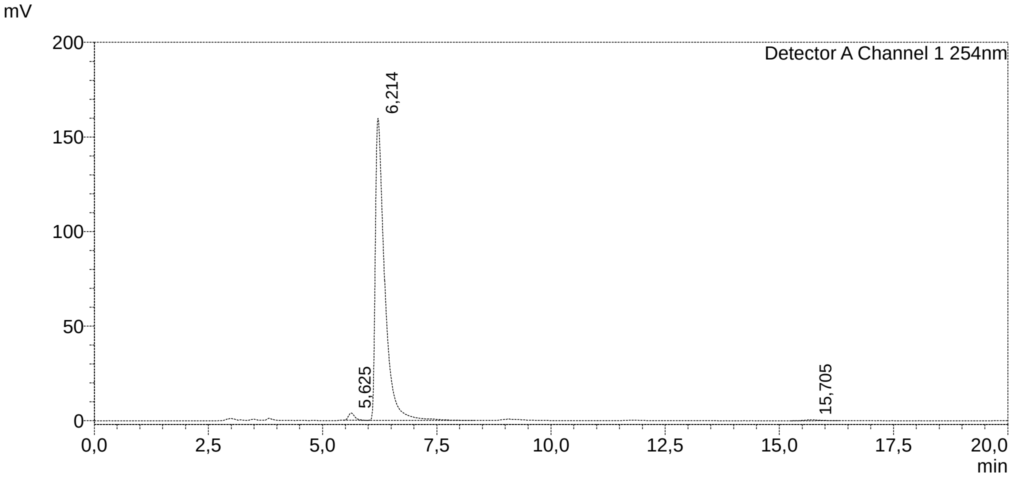

<Peak Table>

Detector A Channel 1 254nm

Peak#	Ret. Time	Area
1	5,625	36397
2	6,214	2282524
3	15,705	12122
Total		2331043

evo-1.1.441

<Chromatogram>

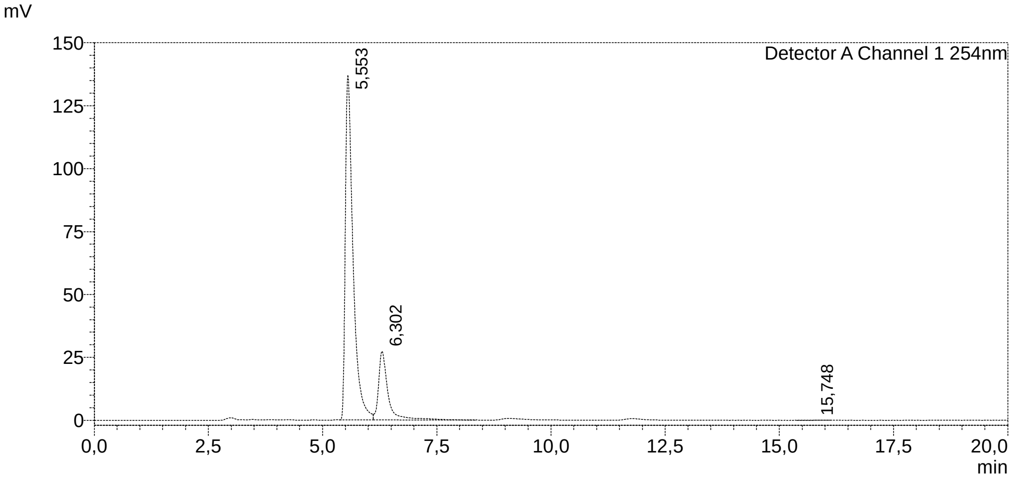

<Peak Table>

Detector A Channel 1 254nm

Peak#	Ret. Time	Area
1	5,553	1618288
2	6,302	397255
3	15,748	1875
Total		2017418

evo-1.1.442

<Chromatogram>

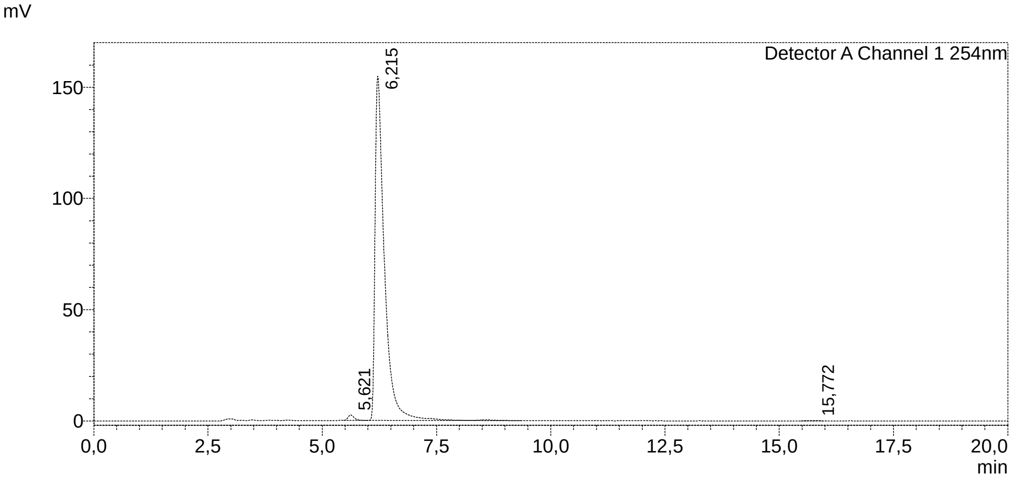

<Peak Table>

Detector A Channel 1 254nm

Peak#	Ret. Time	Area
1	5,621	23107
2	6,215	2228861
3	15,772	1012
Total		2252979

5) Chiral phase HPLC chromatograms of ADH-catalyzed reduction of 1a to (*R*)-5a and (*S*)-5a

Chiral phase HPLC analyses were carried out on a Shimadzu LC-20AD high performance liquid chromatography system equipped with a Shimadzu SPD-20A UV detector and a Phenomenex Lux 3u Cellulose-2 chiral column (250 mm x 4.6 mm). HPLC conditions: injection volume 10 µL; mobile phase: 70% of petroleum ether and 30% of *i*-PrOH; flow rate: 1 mL min^{-1}; detection λ: 280 nm; temperature: 30°C.

Standards, Rt: (6*S*,8*S*)-gingerdiol (**(*S*)-5a**) 5.1 min; (6*S*,8*R*)-gingerdiol (**(*R*)-5a**) 5.7 min; 6-gingerol (**1a**) 9.8 min

<Chromatogram>

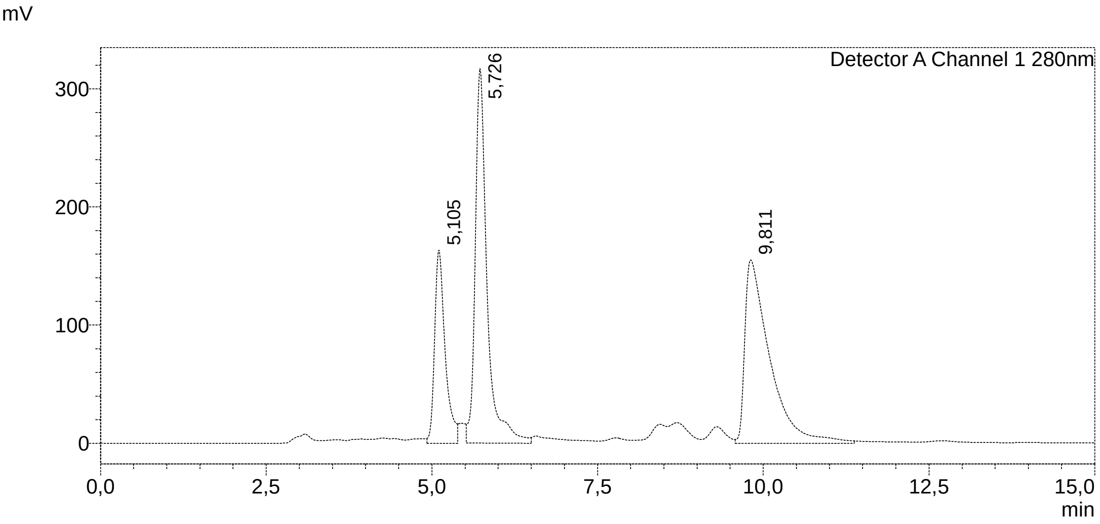

<Peak Table>

Detector A Channel 1 280nm

Peak#	Ret. Time	Area
1	5,105	1828270
2	5,726	3951684
3	9,811	4090562
Total		9870517

Reported chromatograms are named after the ADH employed as biocatalyst and selected based on detectable conversions and des.

Ml-ADH

<Chromatogram>

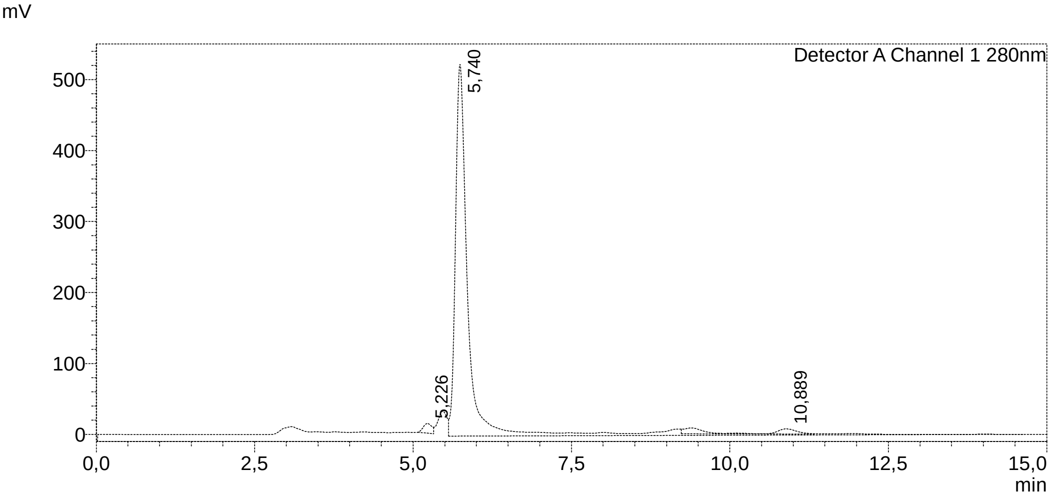

<Peak Table>

Detector A Channel 1 280nm

Peak#	Ret. Time	Area
1	5,226	130067
2	5,740	7698222
3	10,889	336806
Total		8165095

IS2-SDR

<Chromatogram>

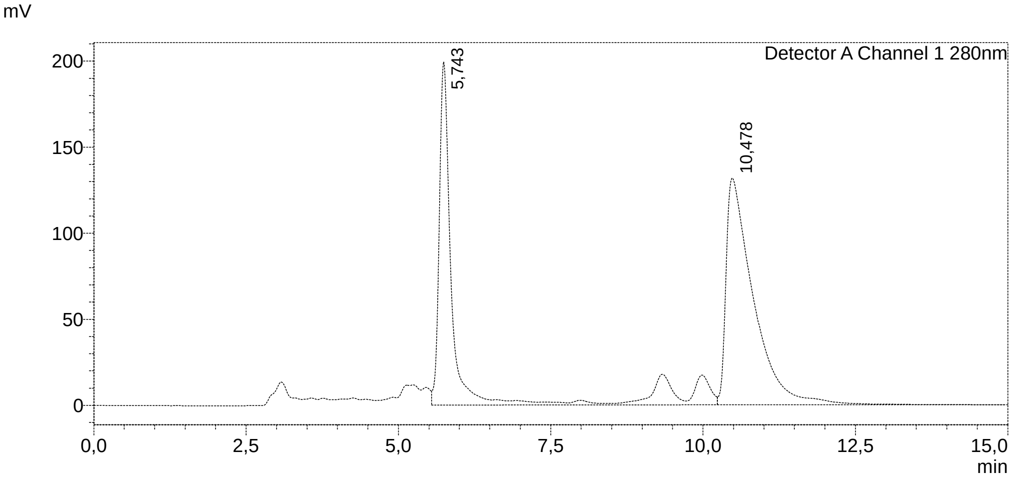

<Peak Table>

Detector A Channel 1 280nm

Peak#	Ret. Time	Area
1	5,743	3490207
2	10,478	4152035
Total		7642242

evo-1.1.010

<Chromatogram>

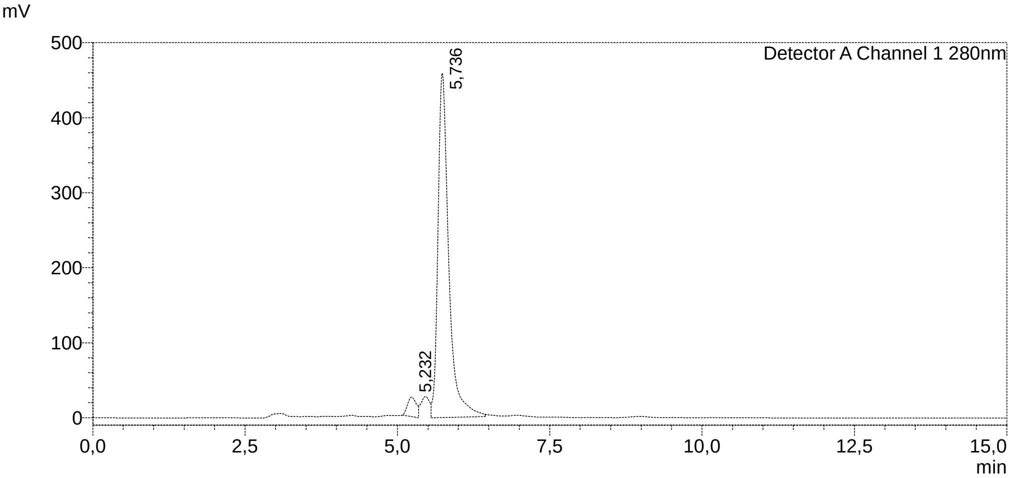

<Peak Table>

Detector A Channel 1 280nm

Peak#	Ret. Time	Area
1	5,232	266640
2	5,736	5669588
Total		5936228

evo-1.1.020

<Chromatogram>

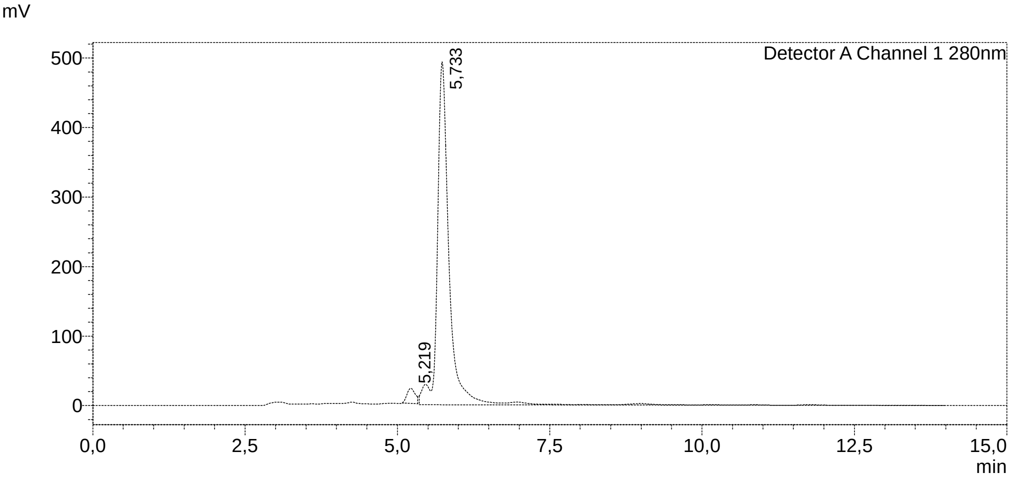

<Peak Table>

Detector A Channel 1 280nm

Peak#	Ret. Time	Area
1	5,219	206583
2	5,733	6683557
Total		6890140

evo-1.1.030

<Chromatogram>

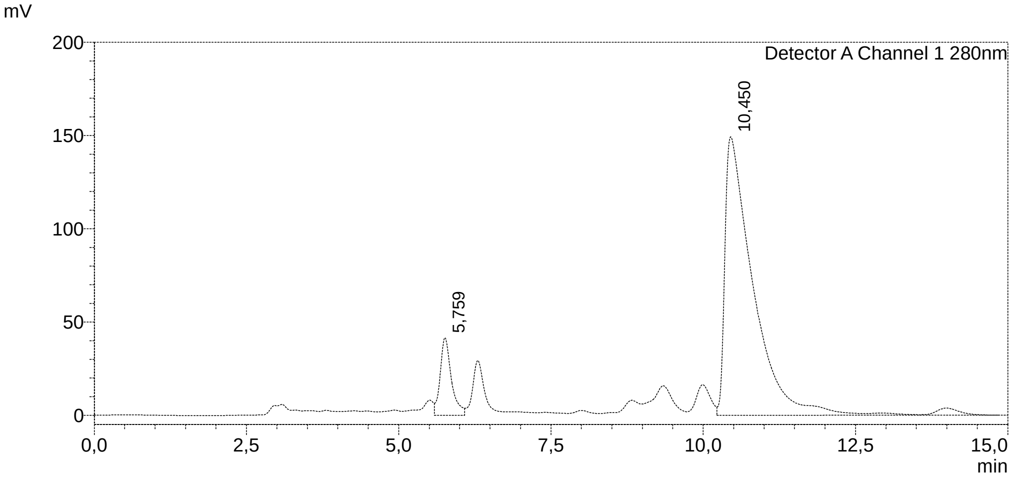

<Peak Table>

Detector A Channel 1 280nm

Peak#	Ret. Time	Area
1	5,759	537026
2	10,450	4912154
Total		5449180

evo-1.1.130

<Chromatogram>

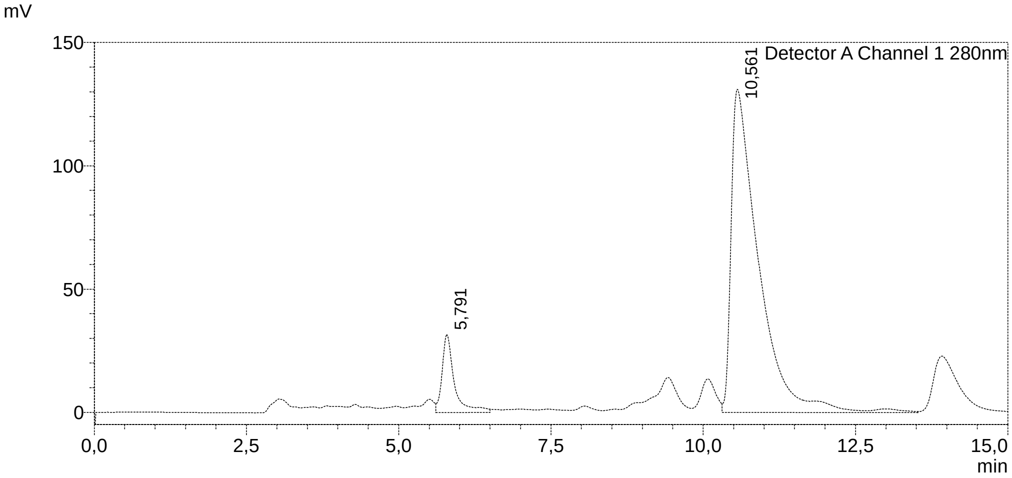

<Peak Table>

Detector A Channel 1 280nm

Peak#	Ret. Time	Area
1	5,791	448822
2	10,561	4131930
Total		4580752

evo-1.1.200

<Chromatogram>

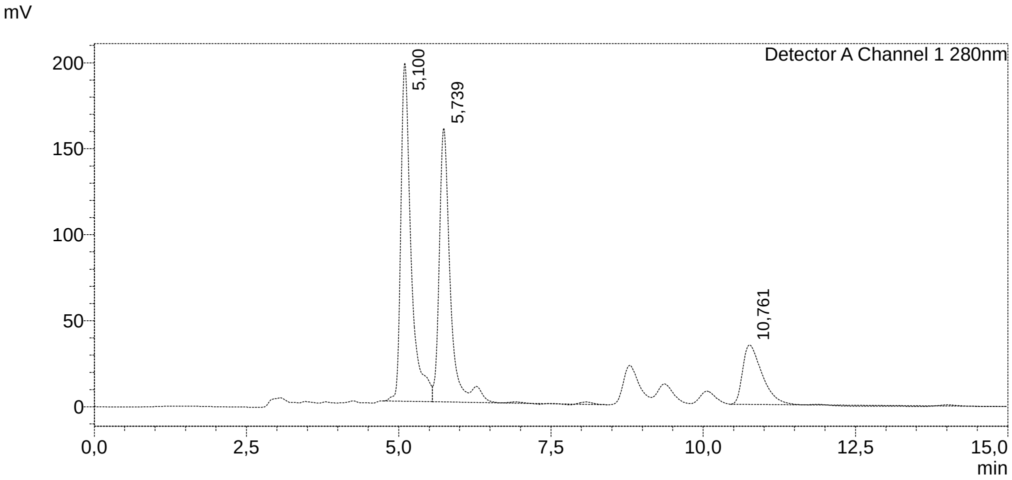

<Peak Table>

Detector A Channel 1 280nm

Peak#	Ret. Time	Area
1	5,100	2325647
2	5,739	2056838
3	10,761	737045
Total		5119530

evo-1.1.210

<Chromatogram>

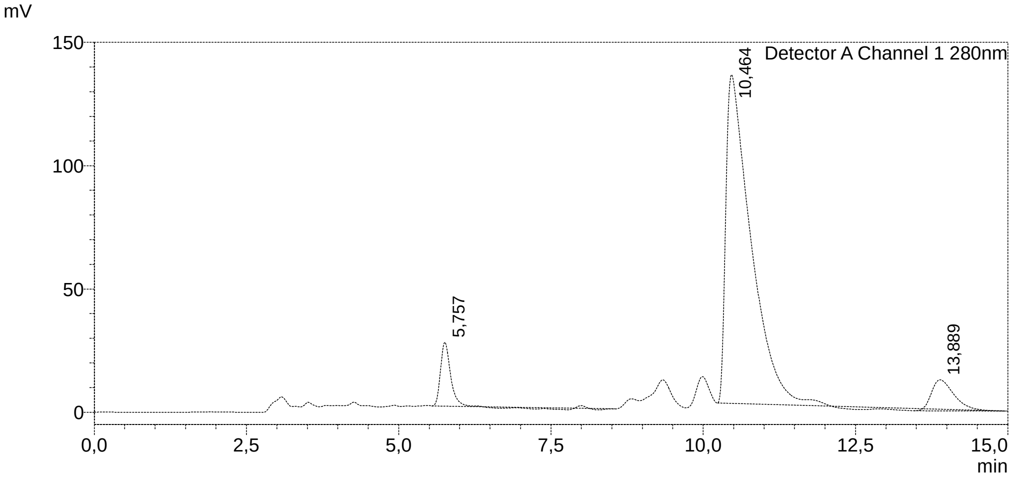

<Peak Table>

Detector A Channel 1 280nm

Peak#	Ret. Time	Area
1	5,757	266923
2	10,464	3759853
3	13,889	334974
Total		4361749

evo-1.1.250

<Chromatogram>

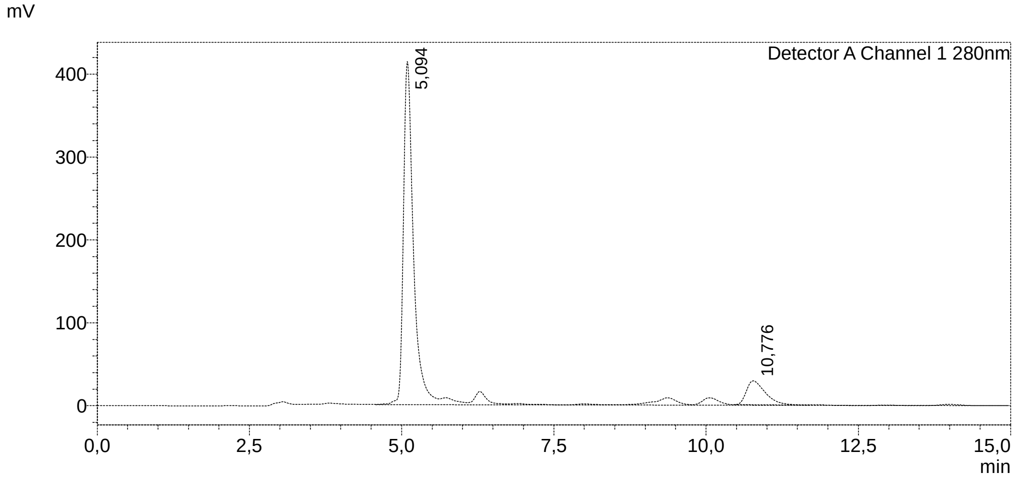

<Peak Table>

Detector A Channel 1 280nm

Peak#	Ret. Time	Area
1	5,094	5667193
2	10,776	625632
Total		6292825

evo-1.1.260

<Chromatogram>

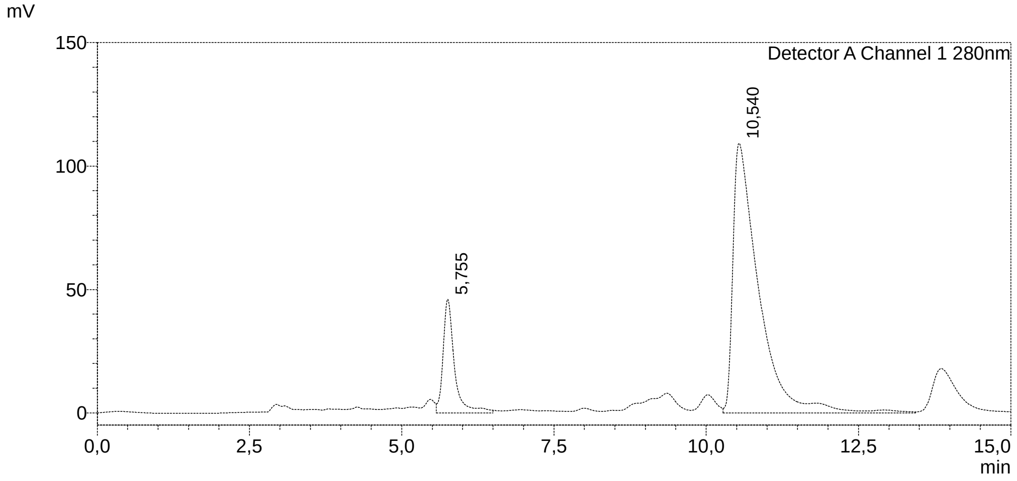

<Peak Table>

Detector A Channel 1 280nm

Peak#	Ret. Time	Area
1	5,755	604902
2	10,540	3238162
Total		3843064

evo-1.1.270

<Chromatogram>

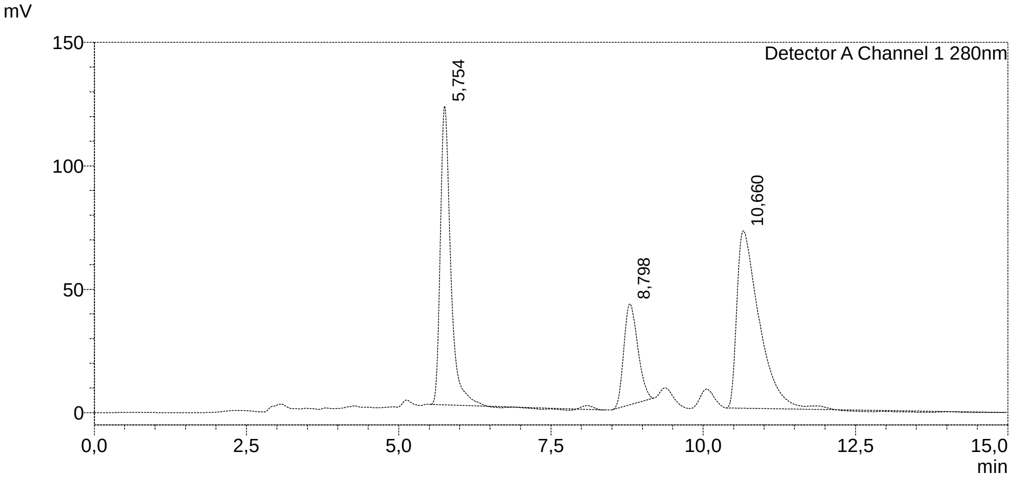

<Peak Table>

Detector A Channel 1 280nm

Peak#	Ret. Time	Area
1	5,754	1441596
2	8,798	650881
3	10,660	1812225
Total		3904703

evo-1.1.380

<Chromatogram>

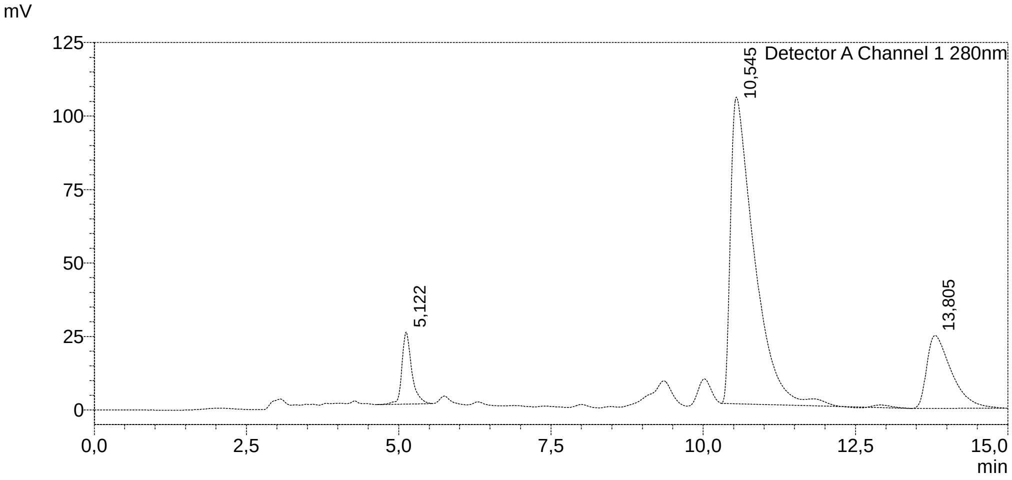

<Peak Table>

Detector A Channel 1 280nm

Peak#	Ret. Time	Area
1	5,122	275105
2	10,545	2862964
3	13,805	699919
Total		3837988

evo-1.1.420

<Chromatogram>

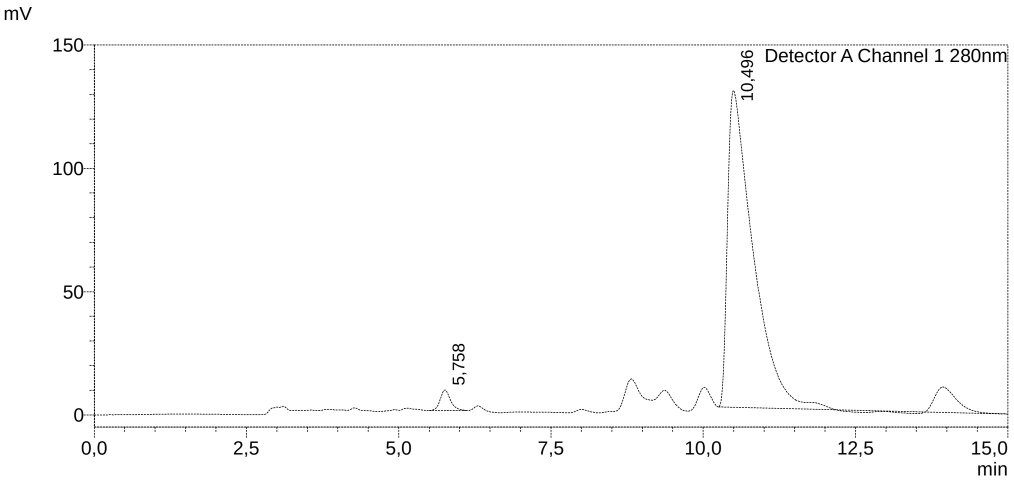

<Peak Table>

Detector A Channel 1 280nm

Peak#	Ret. Time	Area
1	5,758	97701
2	10,496	4001029
Total		4098730

evo-1.1.430

<Chromatogram>

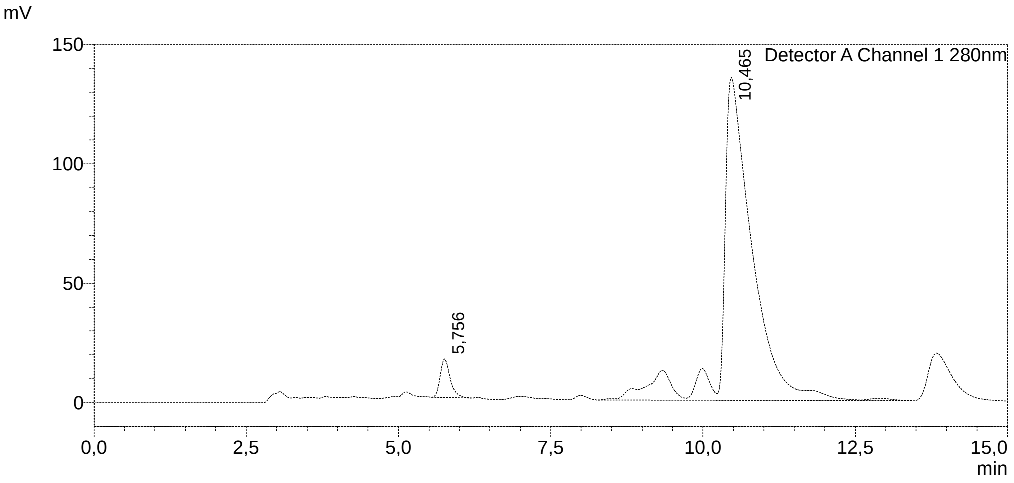

<Peak Table>

Detector A Channel 1 280nm

Peak#	Ret. Time	Area
1	5,756	185367
2	10,465	4718386
Total		4903753

evo-1.1.440

<Chromatogram>

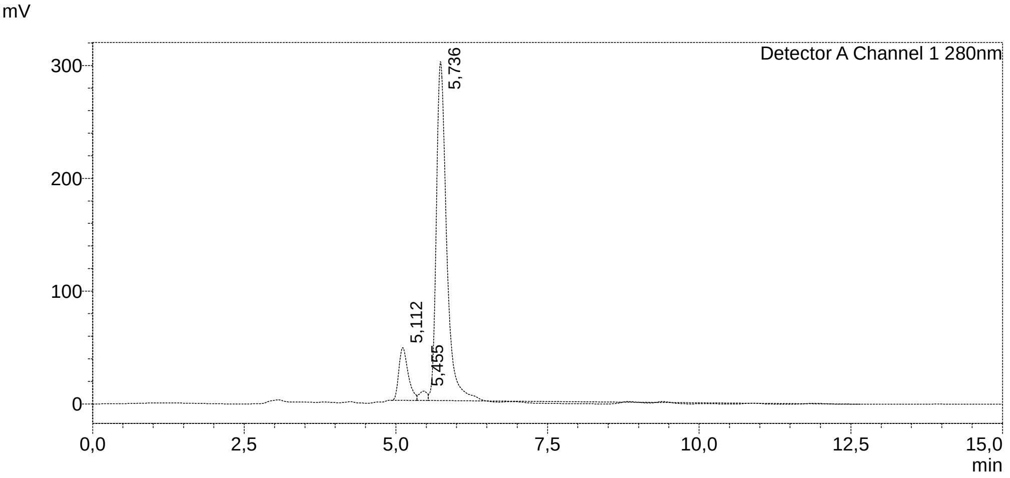

<Peak Table>

Detector A Channel 1 280nm

Peak#	Ret. Time	Area
1	5,112	500977
2	5,455	74386
3	5,736	3363655
Total		3939018

evo-1.1.441

<Chromatogram>

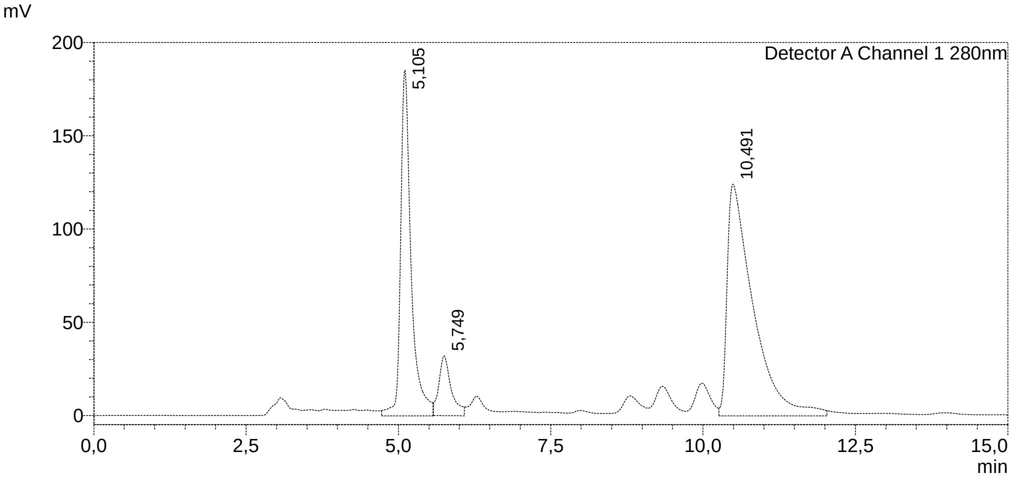

<Peak Table>

Detector A Channel 1 280nm

Peak#	Ret. Time	Area
1	5,105	2219926
2	5,749	454551
3	10,491	3714952
Total		6389430

evo-1.1.442

<Chromatogram>

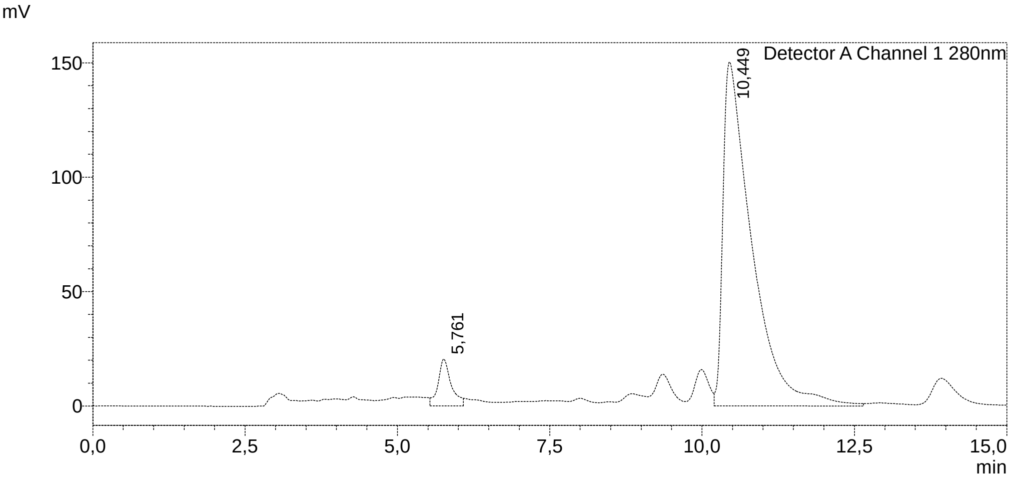

<Peak Table>

Detector A Channel 1 280nm

Peak#	Ret. Time	Area
1	5,761	308140
2	10,449	4990678
Total		5298818

6) UPLC-UV chromatograms of ADH-catalyzed reduction of 1b to (*R*)-5b and (*S*)-5b

The chromatographic analyses were performed on Waters ACQUITY UPLC system (Waters corp., MA, United States) equipped with a quaternary pump, autosampler, thermostated column compartment and a dual-wavelength UV/Visible (UV/Vis) detector (TUV). The data were processed with Empower 3 workstations. The employed column was BEH C18 (2.1mm x 50 mm), maintained at 30°C, and mobile phase was composed of water containing 0.1% of formic acid (v/v) (A) and acetonitrile containing 0.1% of formic acid (v/v) (B). The linear gradient elution used was in according to Li Y. et al. with suitable modifications.[12]
Retention times: 8-gingerol (**1b**) 10.7 min; **(*R*)-5b** 10.5 min; **(*S*)-5b** 10.2 min.

Reported chromatograms are named after the ADH employed as biocatalyst and selected based on detectable conversions and des.

<u>Ml-ADH</u>

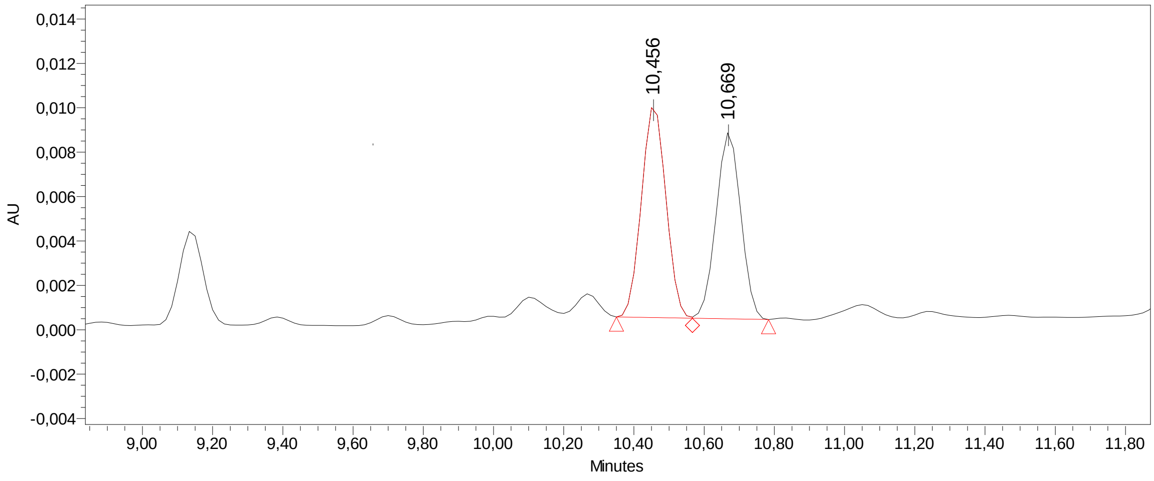

(*R*)-5b = 53,05%
1b = 46,95%

<u>evo-1.1.010</u>

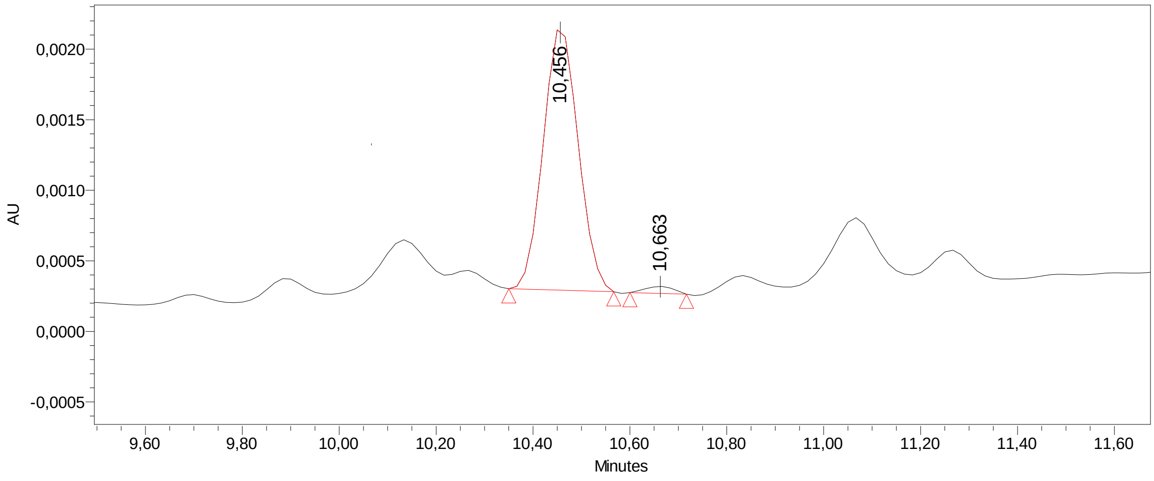

(*R*)-5b = 97,9%
1b = 2,1%

evo-1.1.020

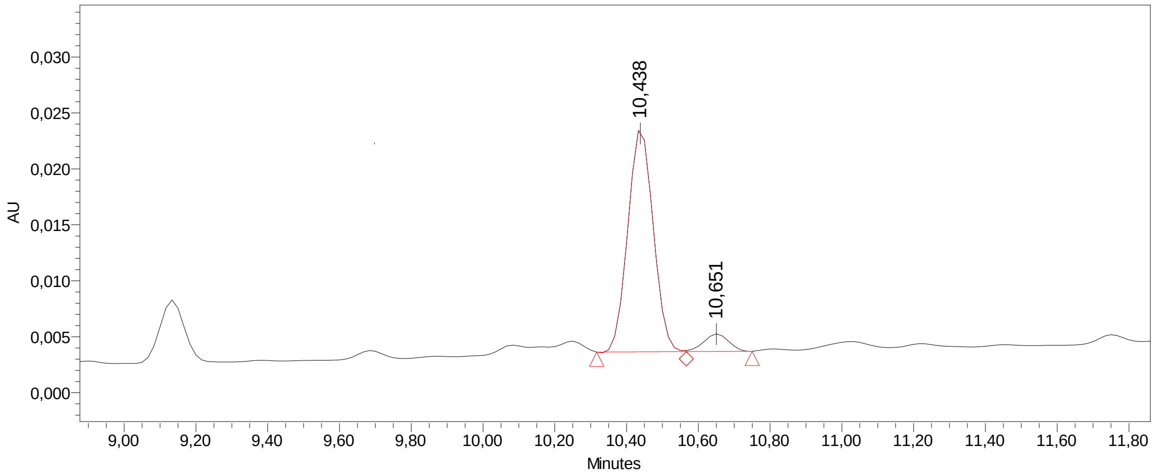

(*R*)-5b = 92,89%
1b = 7,11%

evo-1.1.440

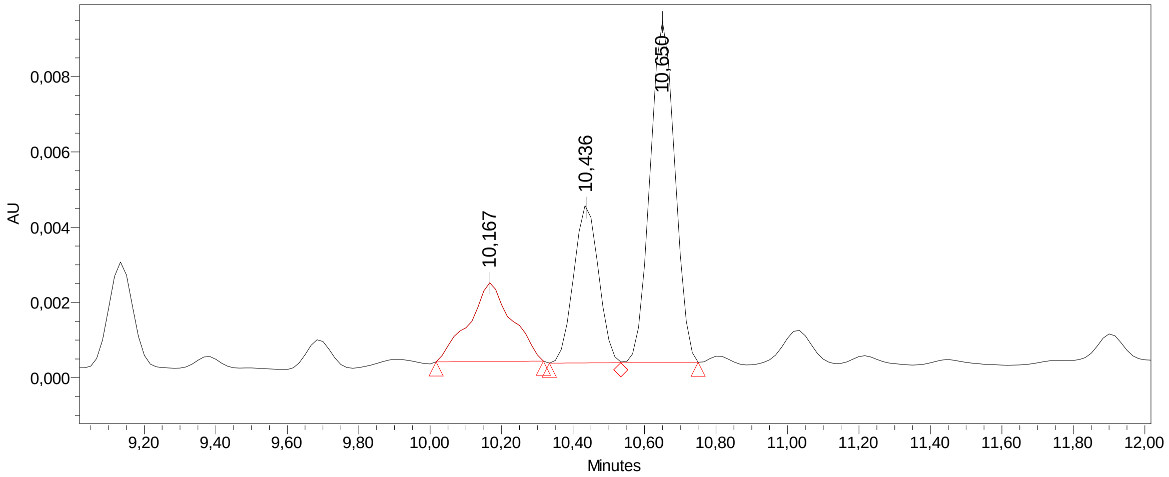

(*R*)-5b = 24,9%
(*S*)-5b = 21,42 %
1b = 53,7%

7) UPLC-UV (Method A) and chiral phase HPLC chromatograms (Method B) of the enzymatic reduction of compound 3 to (*S*)-6 and (*R*)-6

(Method A). The chromatographic analyses were performed on Waters ACQUITY UPLC system (Waters corp., MA, United States) equipped with a quaternary pump, autosampler, thermostated column compartment and a dual-wavelength UV/Visible (UV/Vis) detector (TUV). The data were processed with Empower 3 workstations. The employed column was BEH C18 (2.1mm x 50 mm), maintained at 30°C, and mobile phase was composed of water containing 0.1% of formic acid (v/v) (A) and acetonitrile containing 0.1% of formic acid (v/v) (B). The linear gradient elution used was in according to Li Y. et al.[12] with suitable modifications.
(Method B). Chromatographic resolution of enantiomerically-enriched compounds was carried out at room temperature on HPLC-UV-PDA Waters (Waters 2996 Photodiode Array Detector, Waters 515 HPLC pump) equipped with CHIRALCEL® OD-H [250 mm x 4,6 mm, 5 µm, produced by Daicel Industries Ltd. (Tokyo, Japan)]. The mobile phase was composed by 90% *n*-hexane (*n*-Hex) and 10% 2-propanol (*i*-PrOH). The flow rate was set up at 1 mL min^{-1} and the wavelength set at 280 nm. The data were processed with Empower 3 workstations.
Retention times: **3** 11.7 min; **(*R*)-6** 13.0 min; **(*S*)-6** 16.7 min.

Reported chromatograms are named after the ADH employed as biocatalyst and selected based on detectable conversions and des.

<u>evo-1.1.440</u>

UPLC-UV

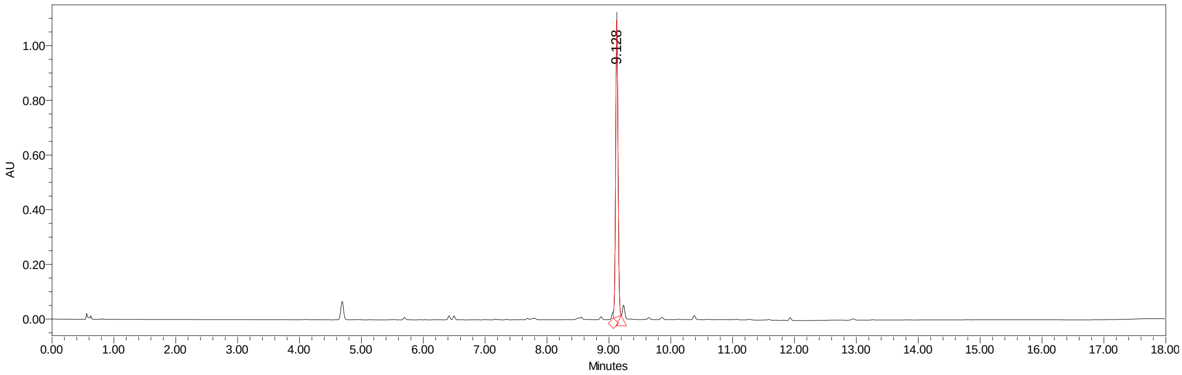

Chiral phase HPLC

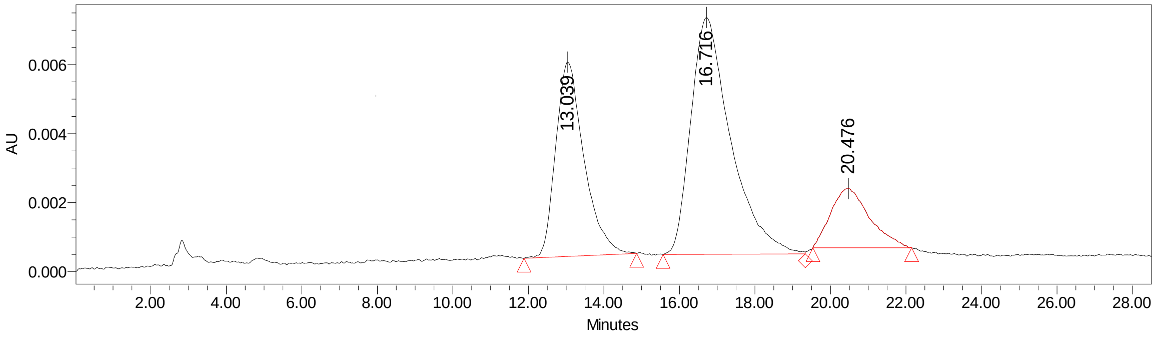

(*R*)-6 = 36.86 %
(*S*)-6 = 63.14 %
ee = 26 %

evo-1.1.250

UPLC-UV

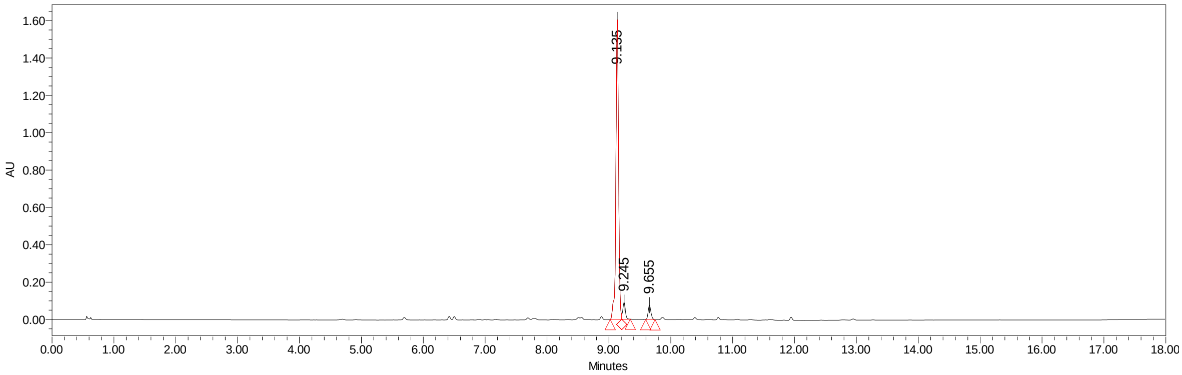

Chiral phase HPLC

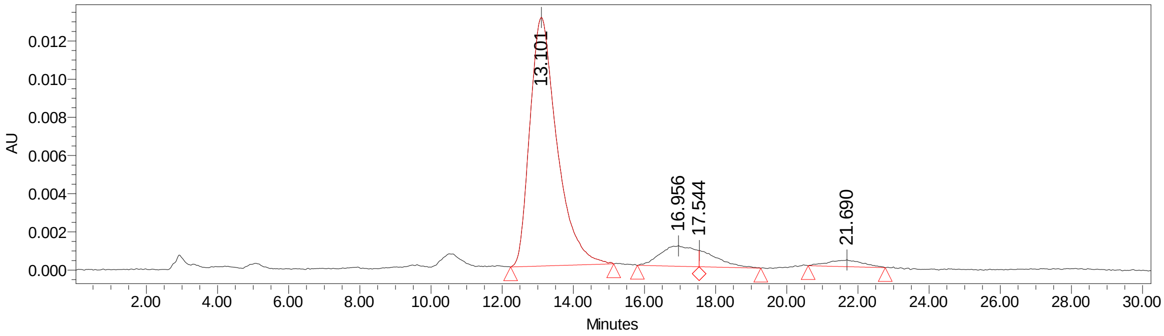

(*R*)-6 = 88.22 %
(*S*)-6 = 11.78 %
ee = 76 %

evo-1.1.020

UPLC-UV

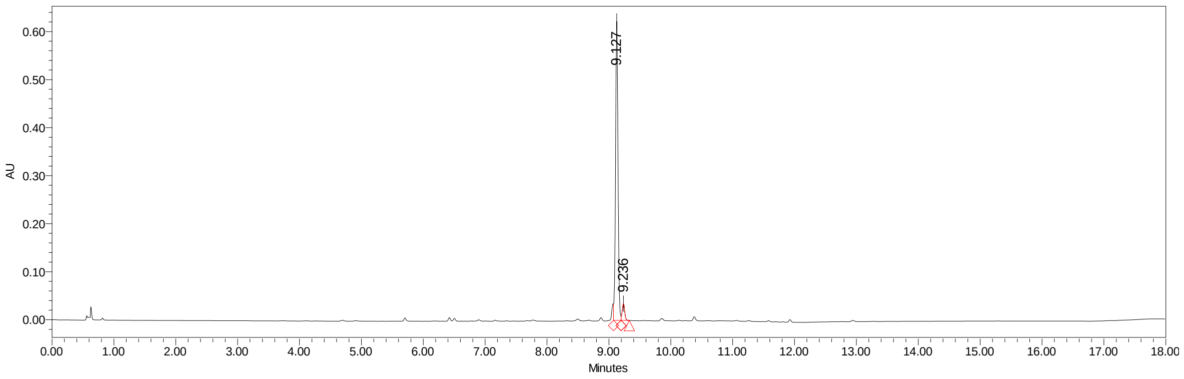

Chiral phase HPLC

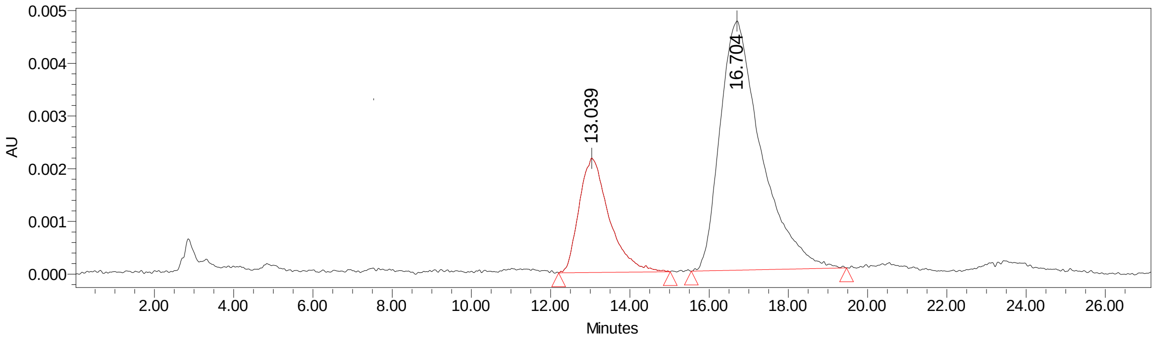

(*R*)-6 = 25.14%
(*S*)-6 = 74.86 %
ee = 50 %

evo-1.1.010

UPLC-UV

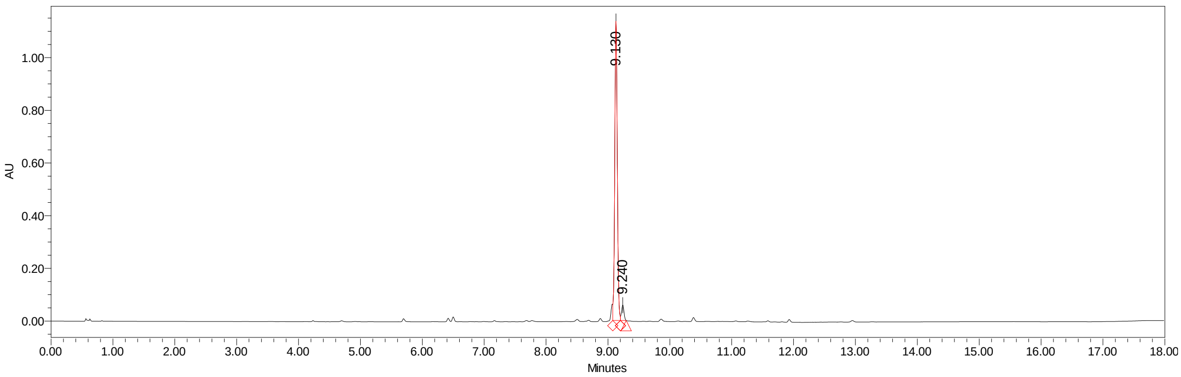

Chiral phase HPLC

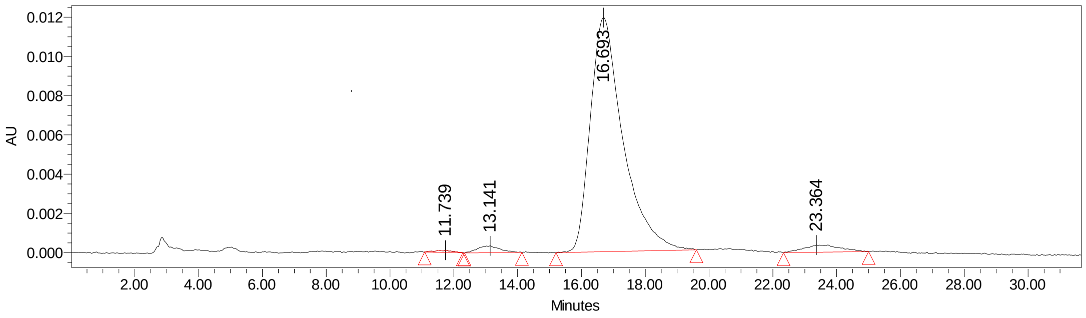

***(R)*-6** = 1.95%
***(S)*-6** = 98.05 %
ee = 96 %

Ml-ADH

UPLC-UV

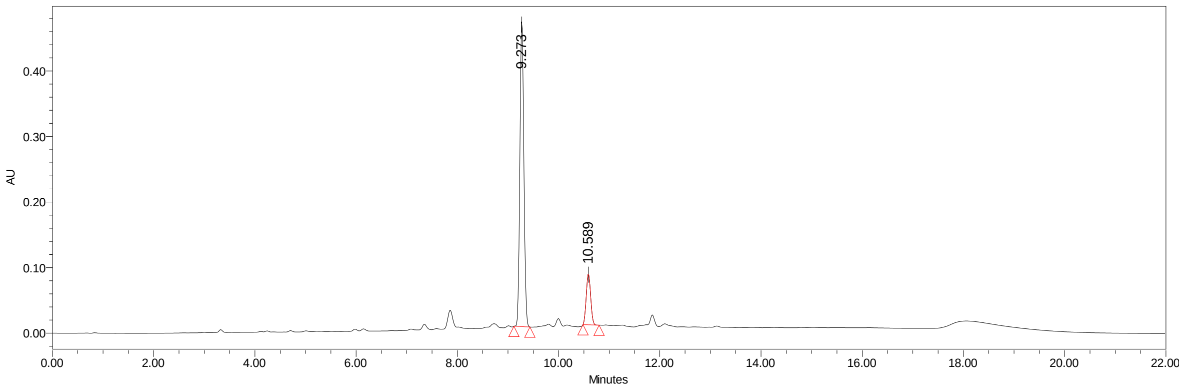

Chiral phase HPLC

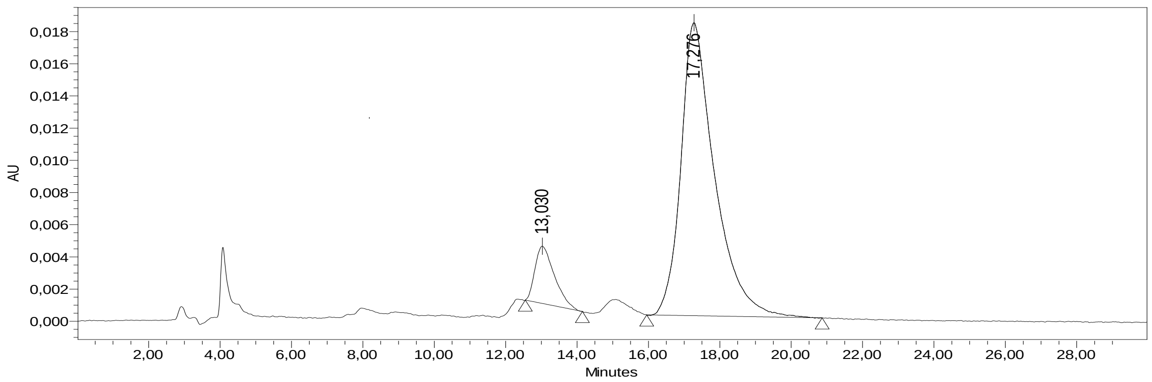

(*R*)-6 = 13.80%
(*S*)-6 = 86.20%
ee = 78 %

8) Characterization of compounds 1a, 1b, 2, 3, 4, (*R*)-5a, (*S*)-5a, (*R*)-5b, (*S*)-5b, 6, (*S*)-6, 7, (*S*)-7, (*R*)-7 (^{1}H-NMR, ^{13}C-NMR, UPLC profiles)

<u>6-gingerol (1a)</u>

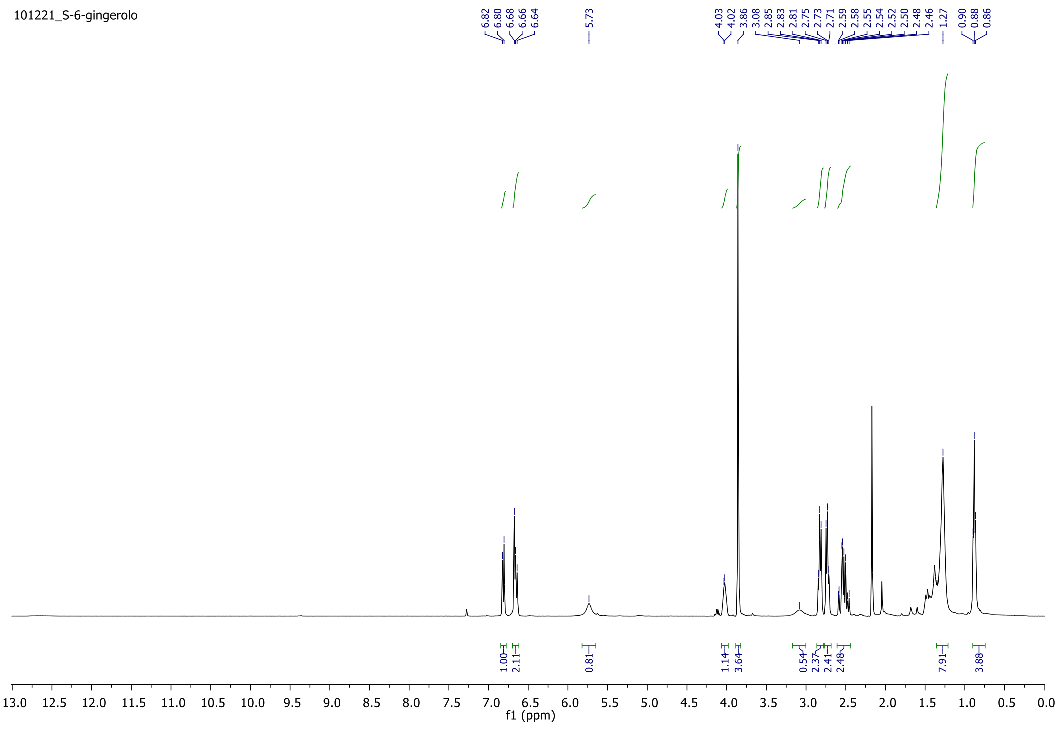

^{1}H-NMR spectra of **1a**; 400 MHz, $CDCl_3$

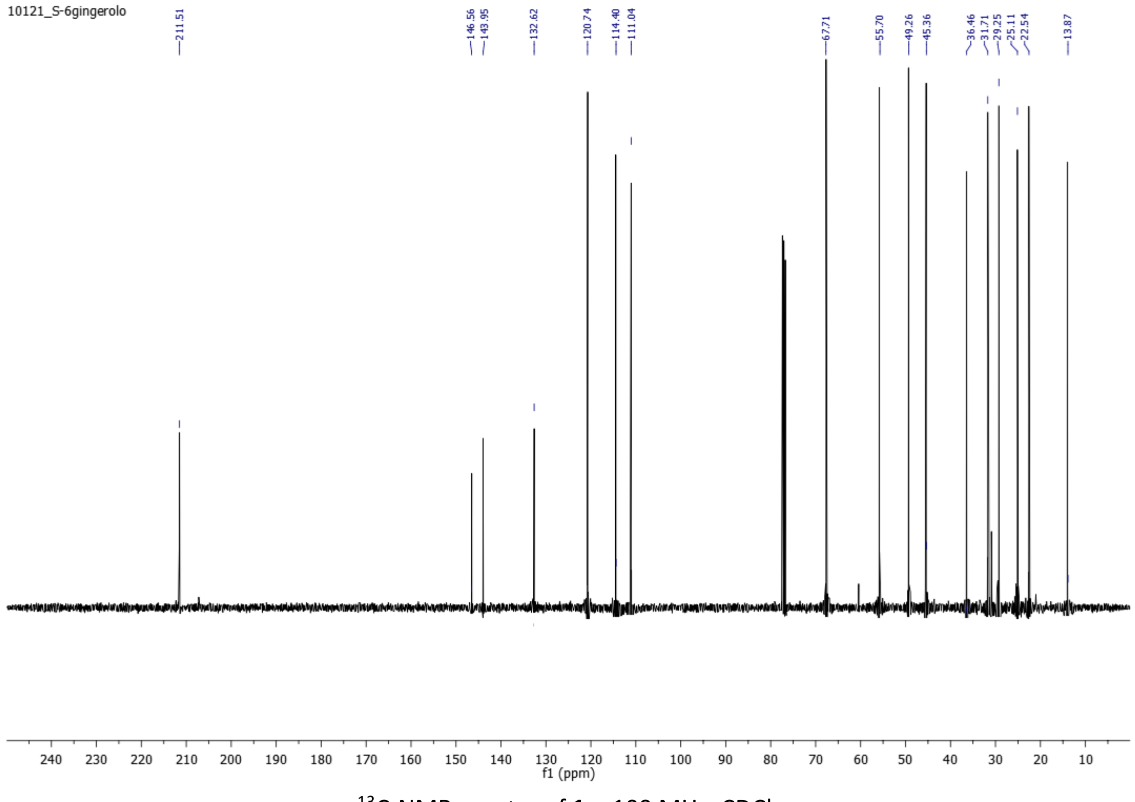

^{13}C-NMR spectra of **1a**; 100 MHz, $CDCl_3$

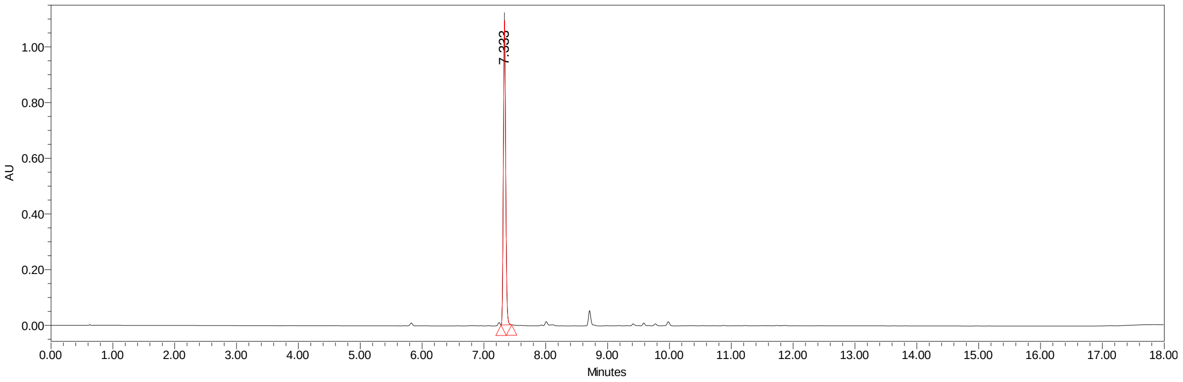

UPLC-UV profile of **1a (Method A)**

8-gingerol (1b)

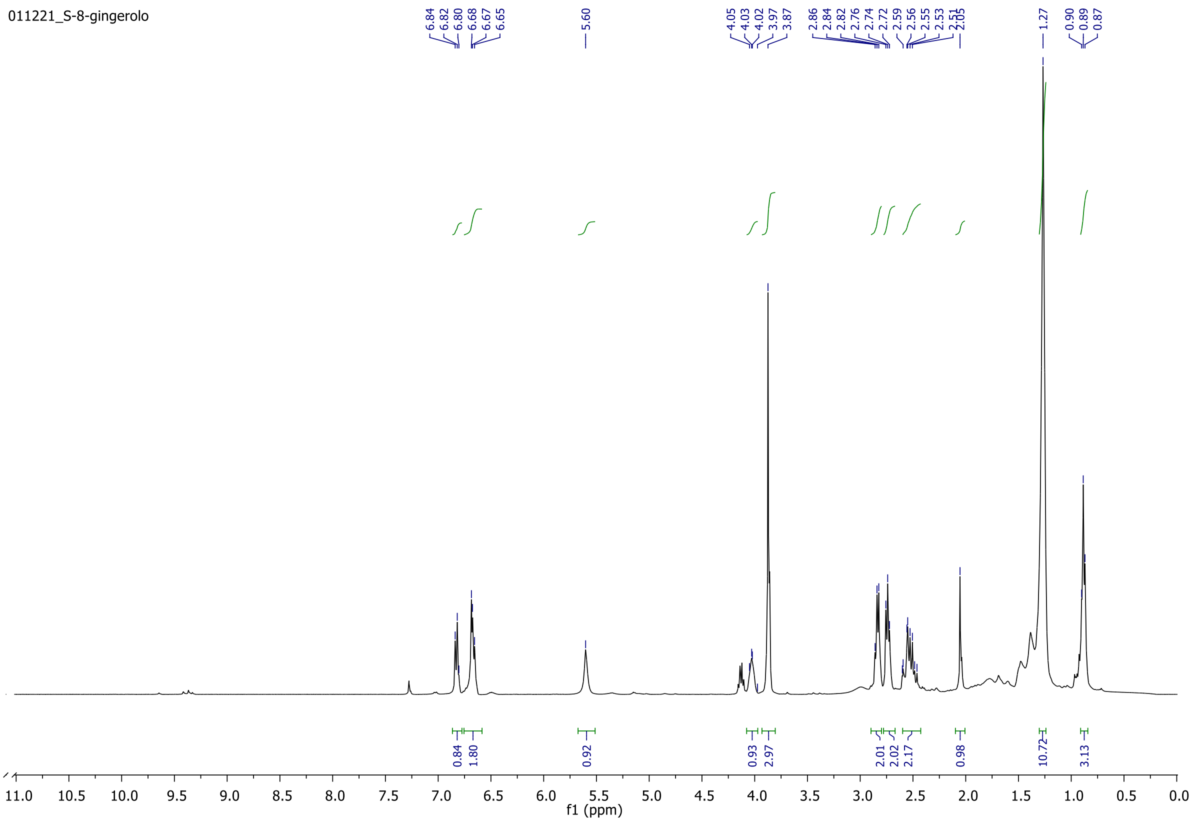

^{1}H-NMR spectra of **1b**; 400 MHz, $CDCl_3$

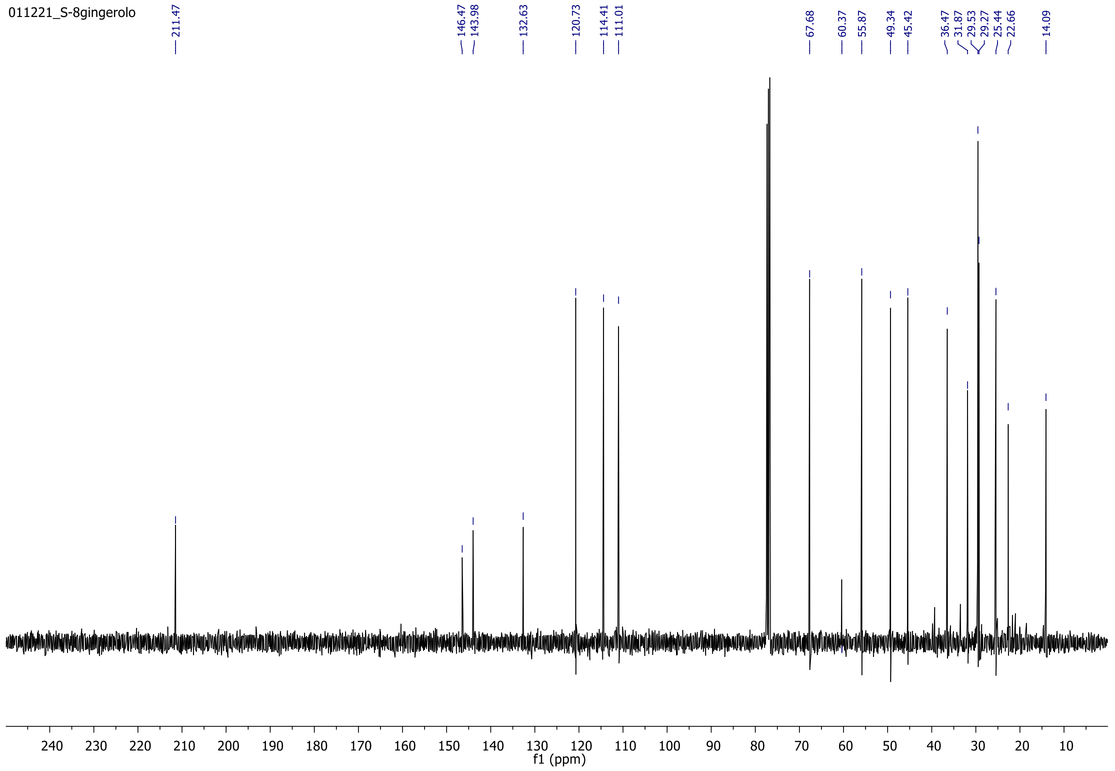

^{13}C-NMR spectra of **1b**; 100 MHz, $CDCl_3$

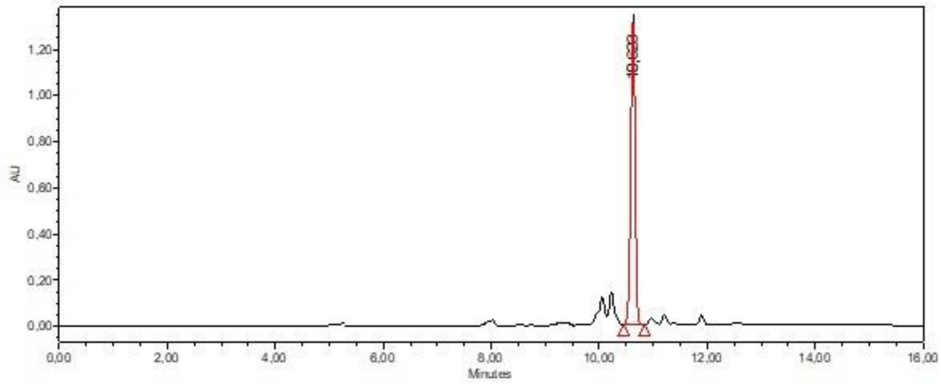

UPLC-UV profile of **1b (Method A)**

6-shogaol (2)

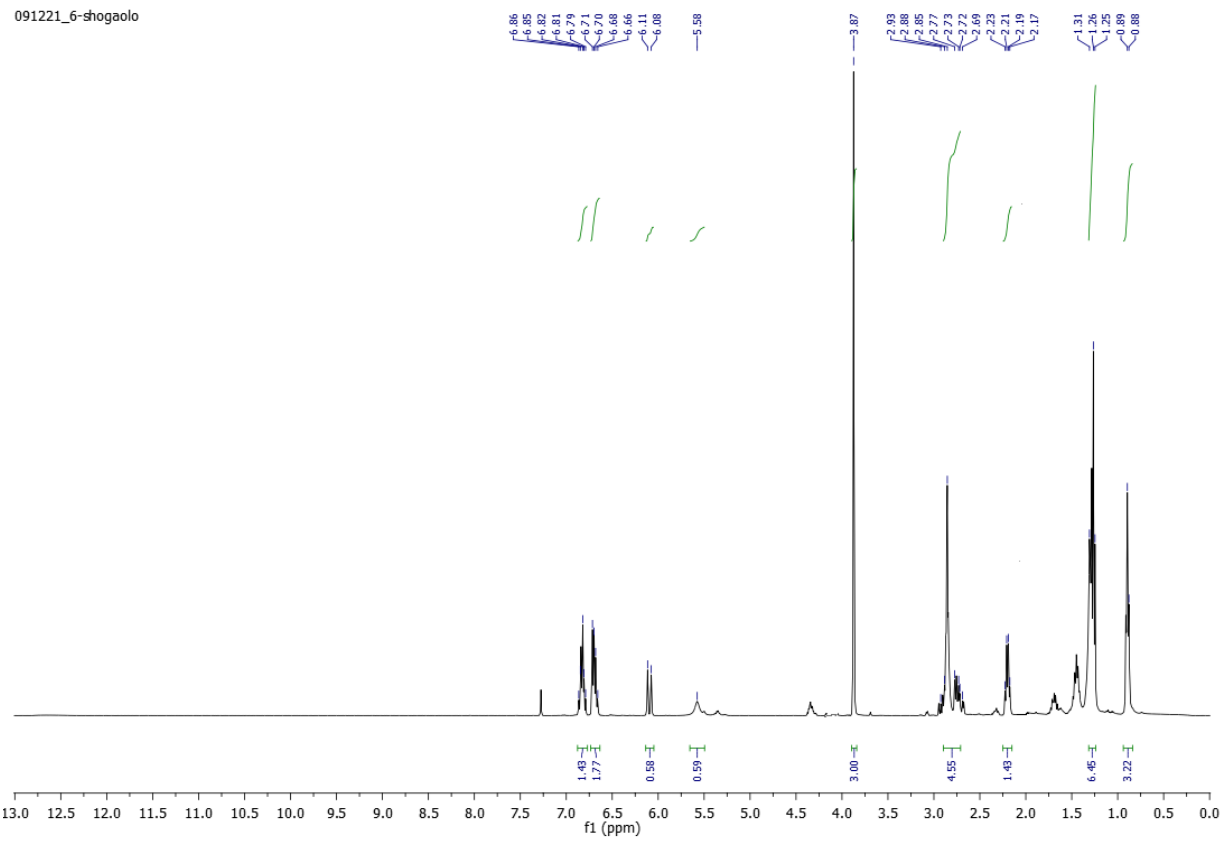

^{1}H-NMR spectra of **2**; 400 MHz, $CDCl_3$

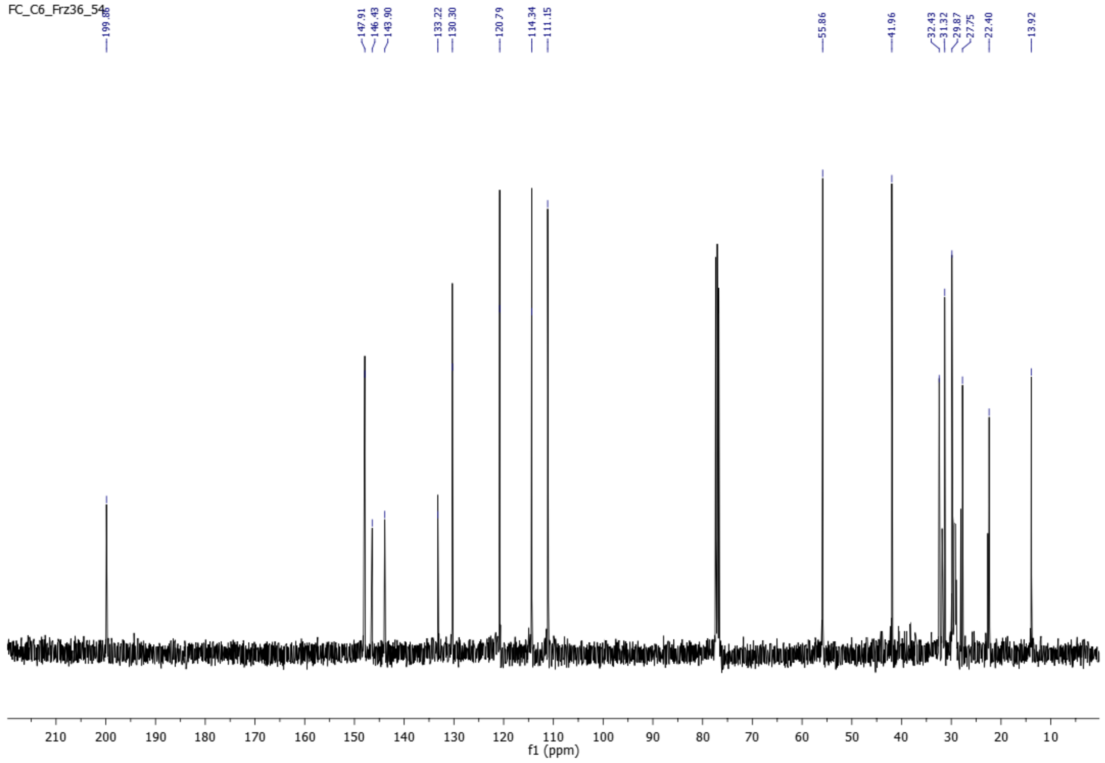

^{13}C-NMR spectra of **2**, 101 MHz, $CDCl_3$

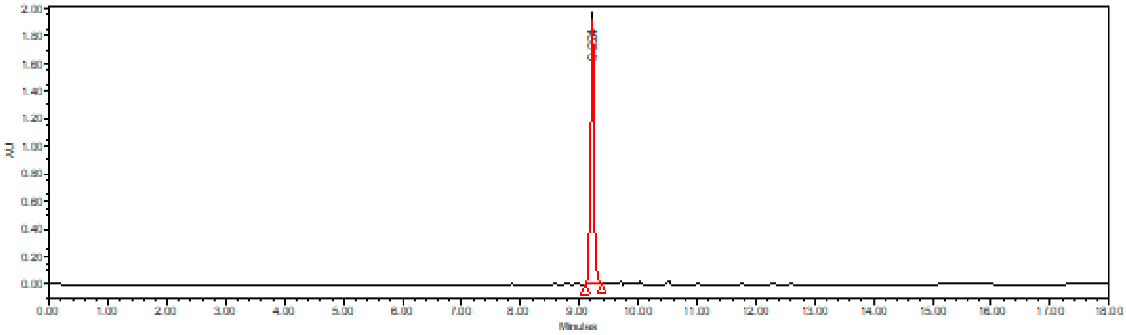

UPLC-UV profile of **2 (Method A)**

6-paradol (3)

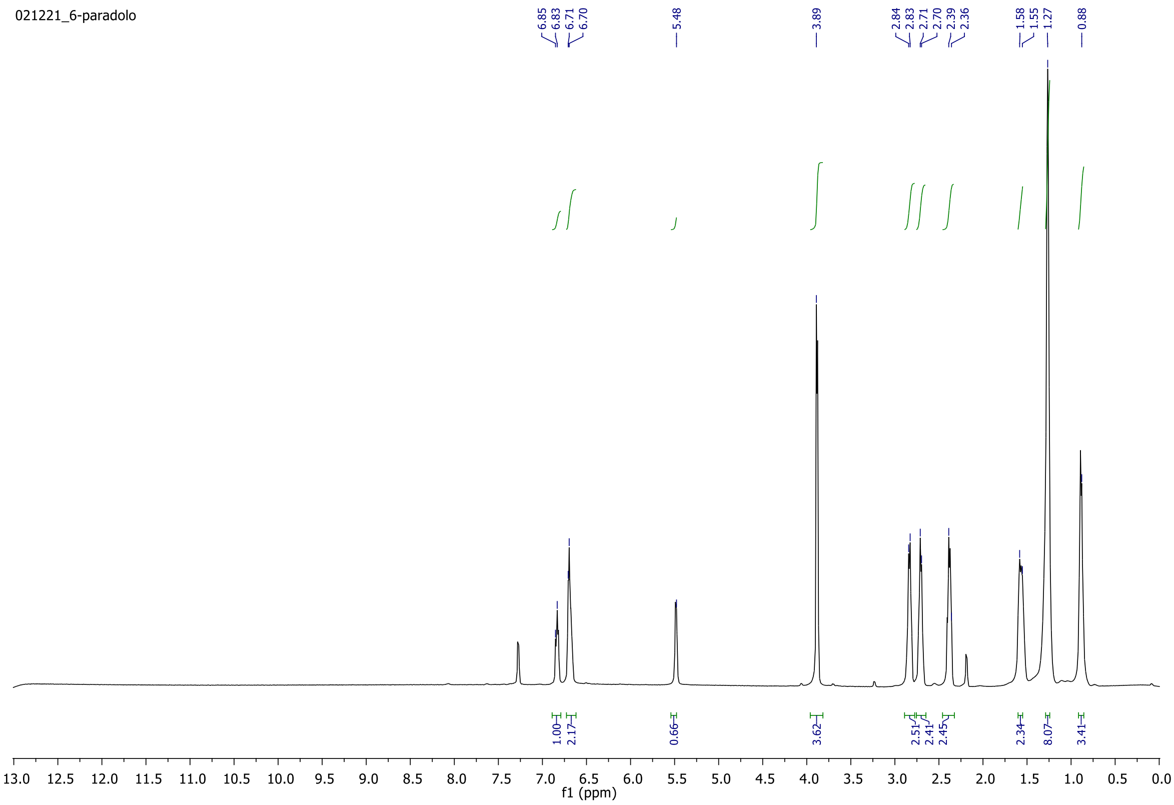

^{1}H-NMR spectra of **3**; 400 MHz, $CDCl_3$

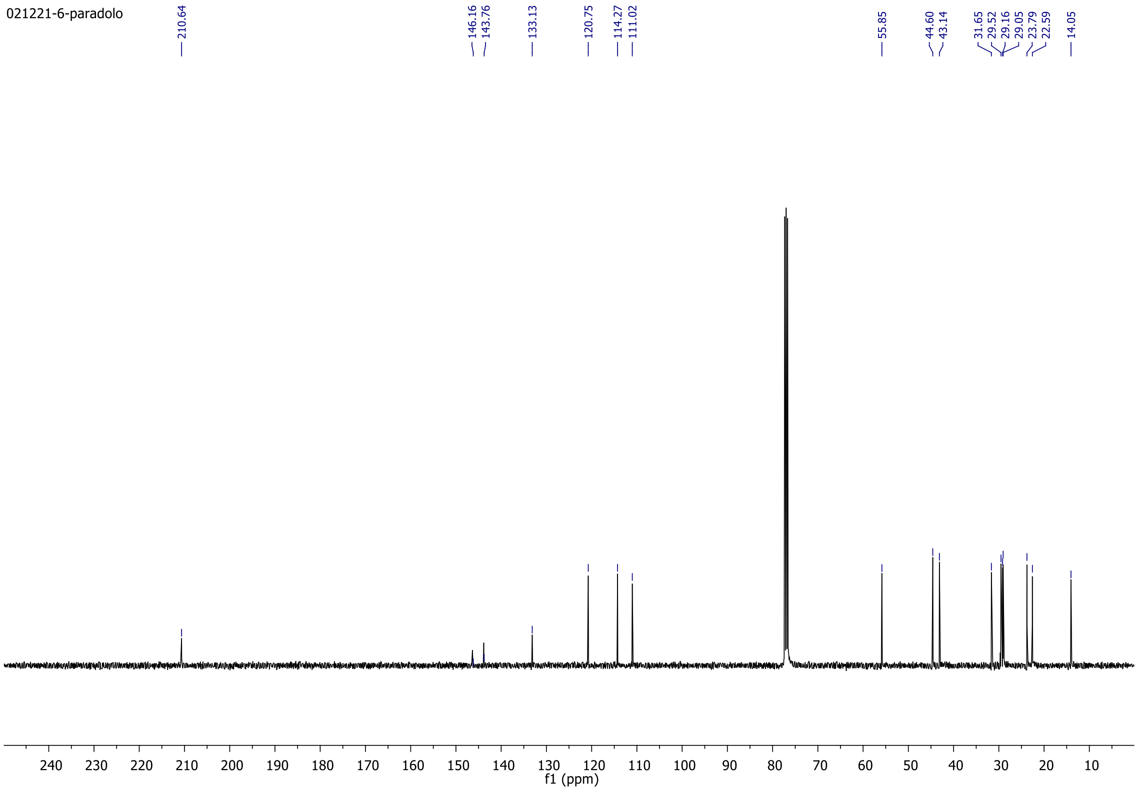

^{13}C-NMR spectra of **3** 101 MHz, $CDCl_3$

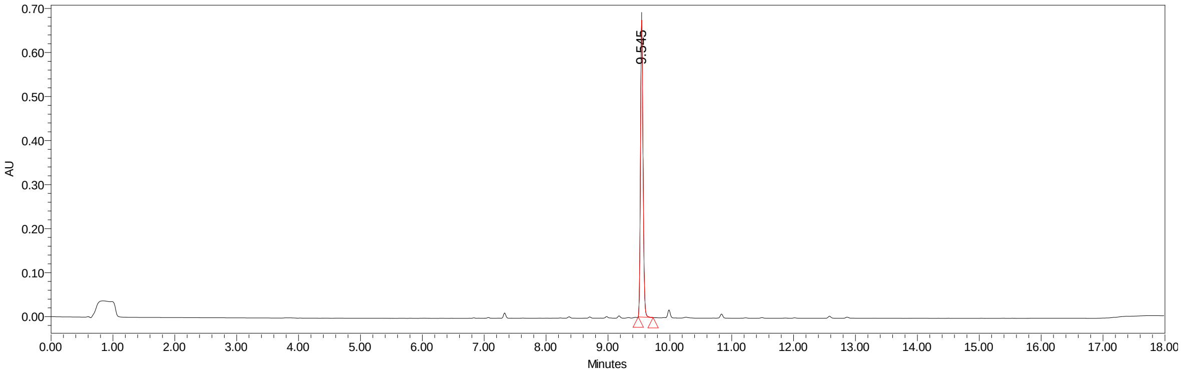

UPLC-UV profile of **3 (Method A)**

6-gingerdiols ((*R*)-5a + (*S*)-5a)

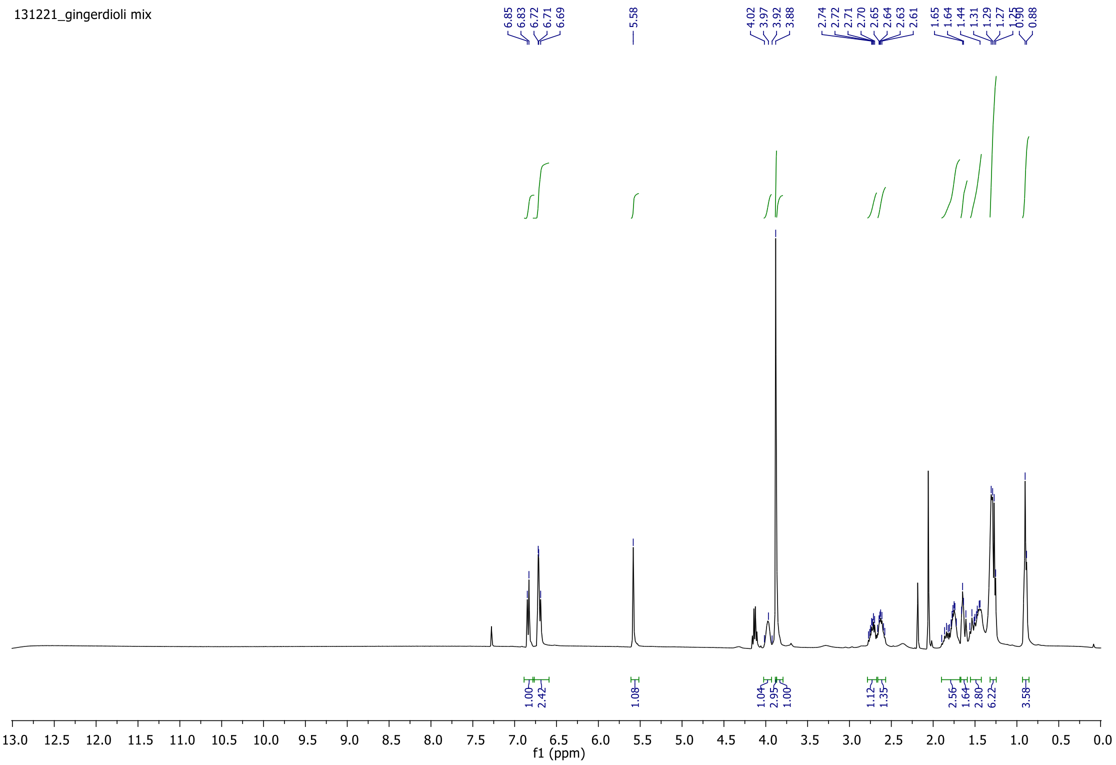

^{1}H-NMR spectra of **(*R*)-5a** + **(*S*)-5a**; 400 MHz, $CDCl_3$

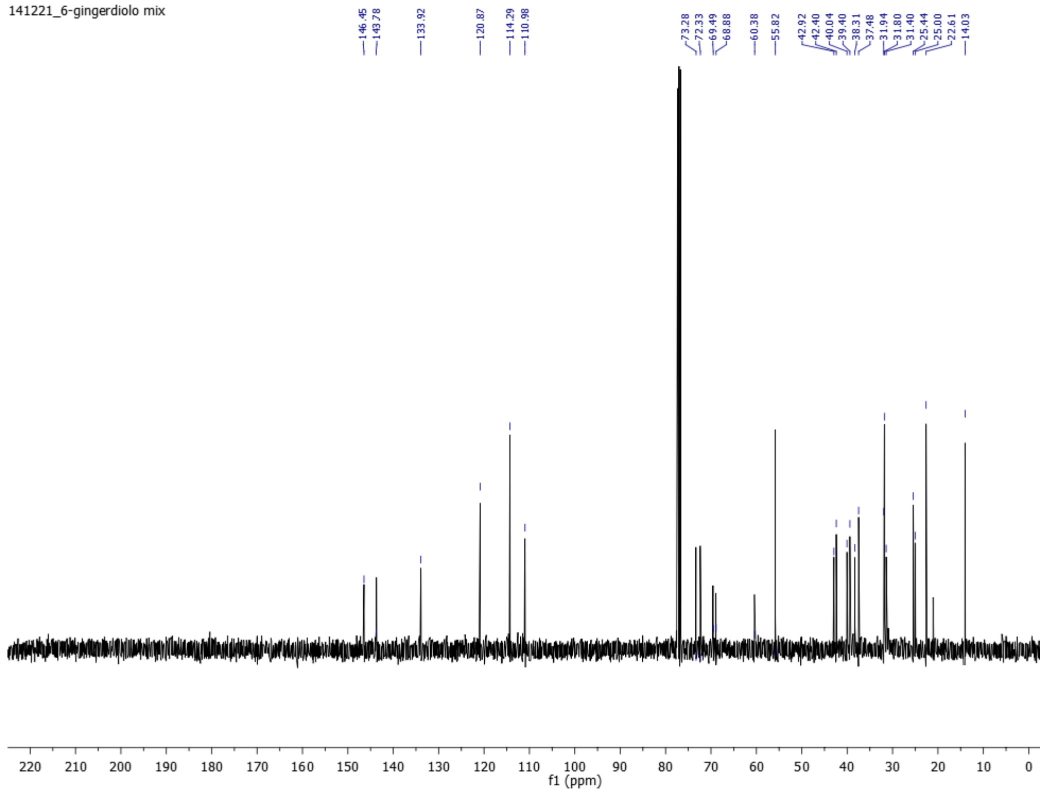

^{13}C-NMR spectra of **(*R*)-5a** + **(*S*)-5a**, 101 MHz, $CDCl_3$

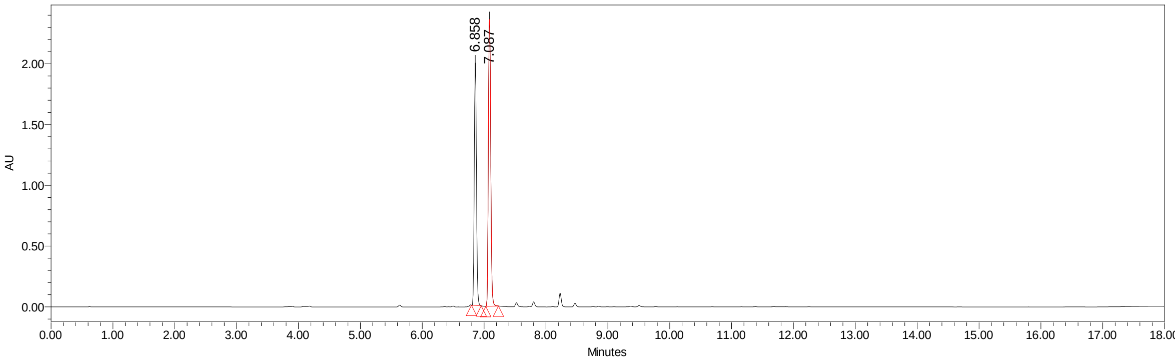

UPLC-UV profile of **(*R*)-5a** + **(*S*)-5a (Method A)**

6*S*, 8*S*-6-gingerol ((*S*)-5a)

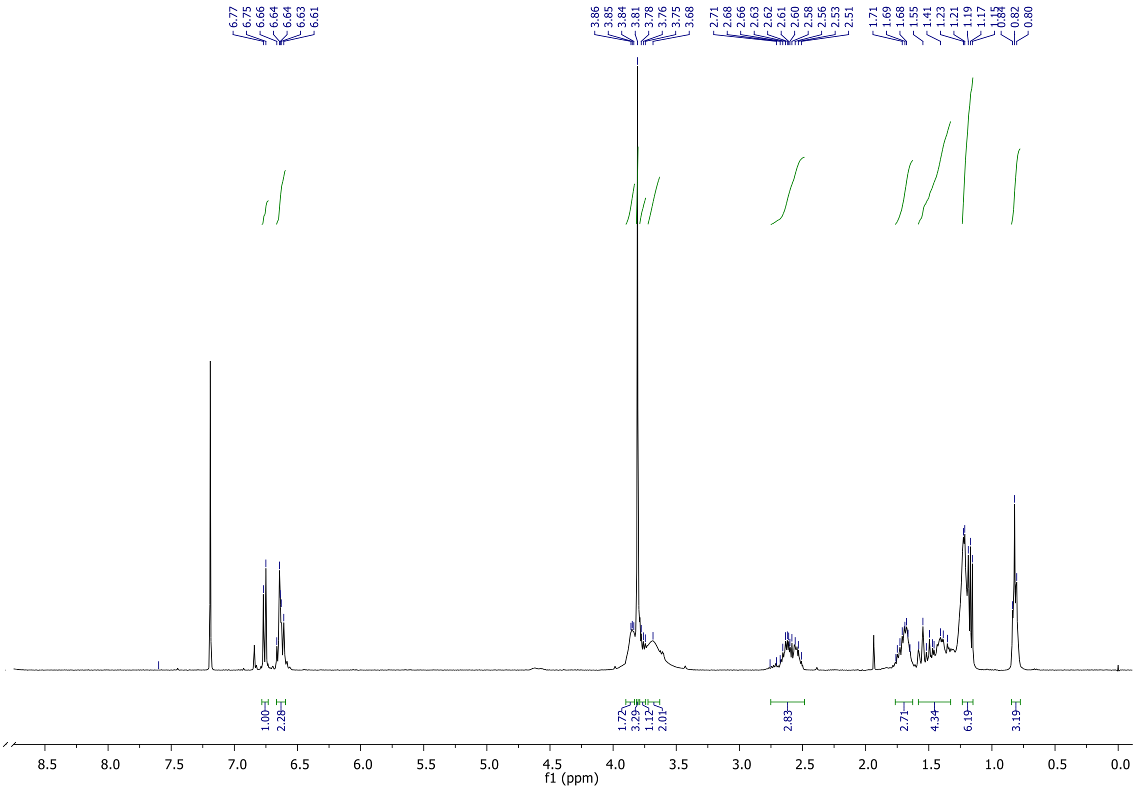

^{1}H-NMR spectra of compound **(*S*)-5a**; 400 MHz, $CDCl_3$

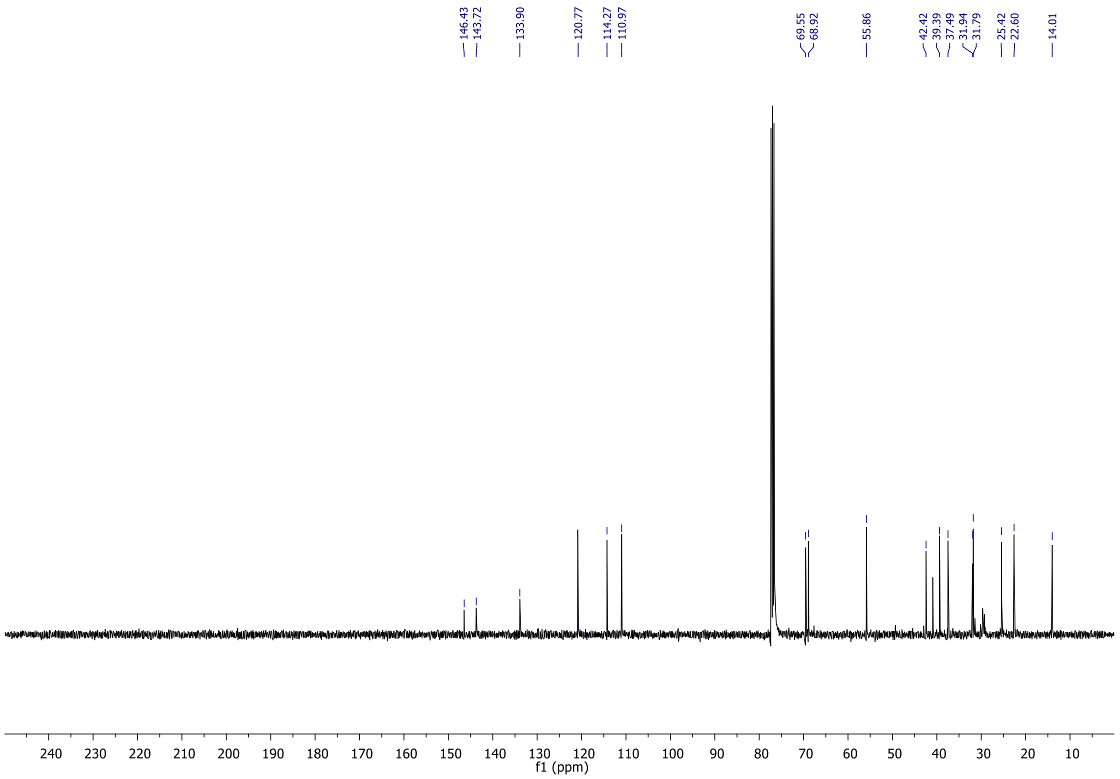

^{13}C-NMR spectra of compound **(*S*)-5a**; 101 MHz, $CDCl_3$

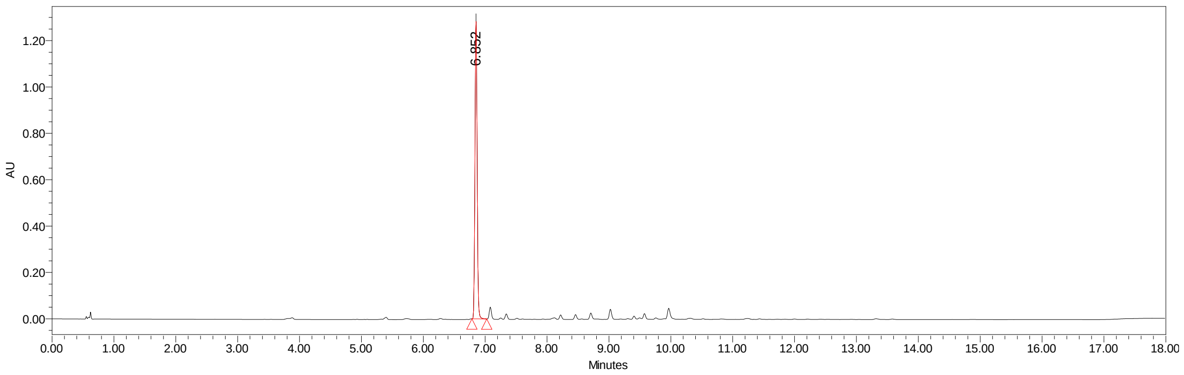

UPLC-UV profile of **(*S*)-5a (Method A)**

6*S*, 8*R*-6-gingerol ((*R*)-5a)

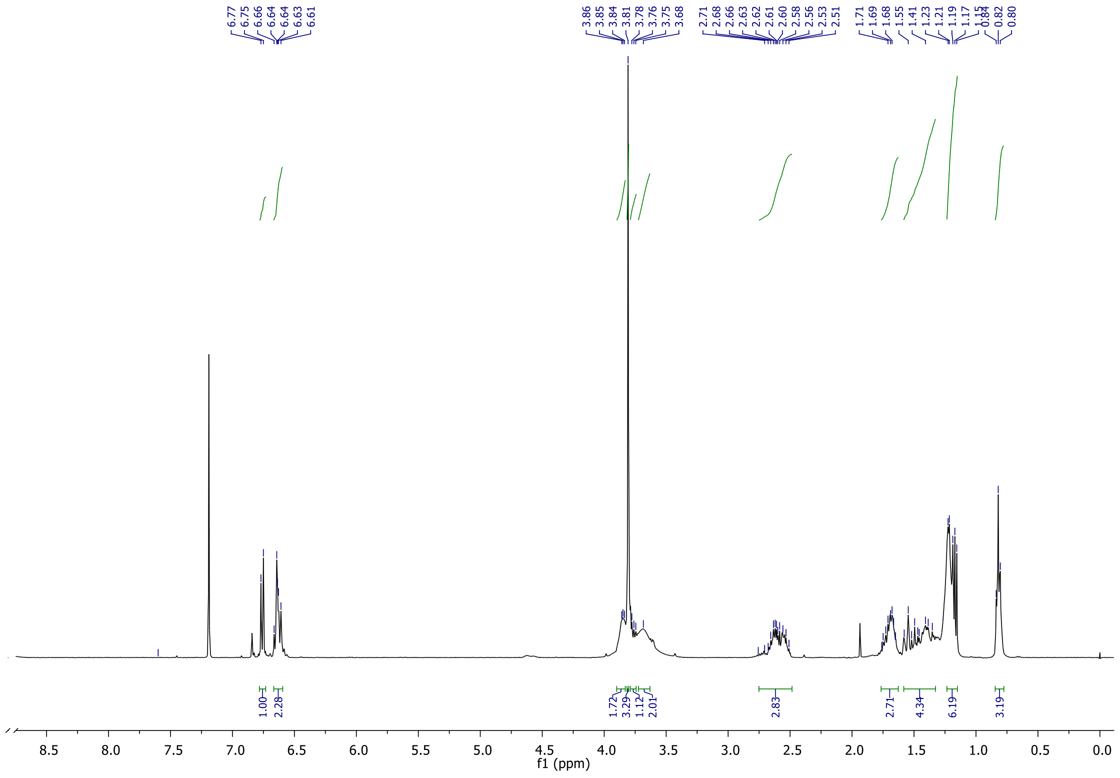

^{1}H-NMR spectra of compound **(*R*)-5a**; 400 MHz, $CDCl_3$

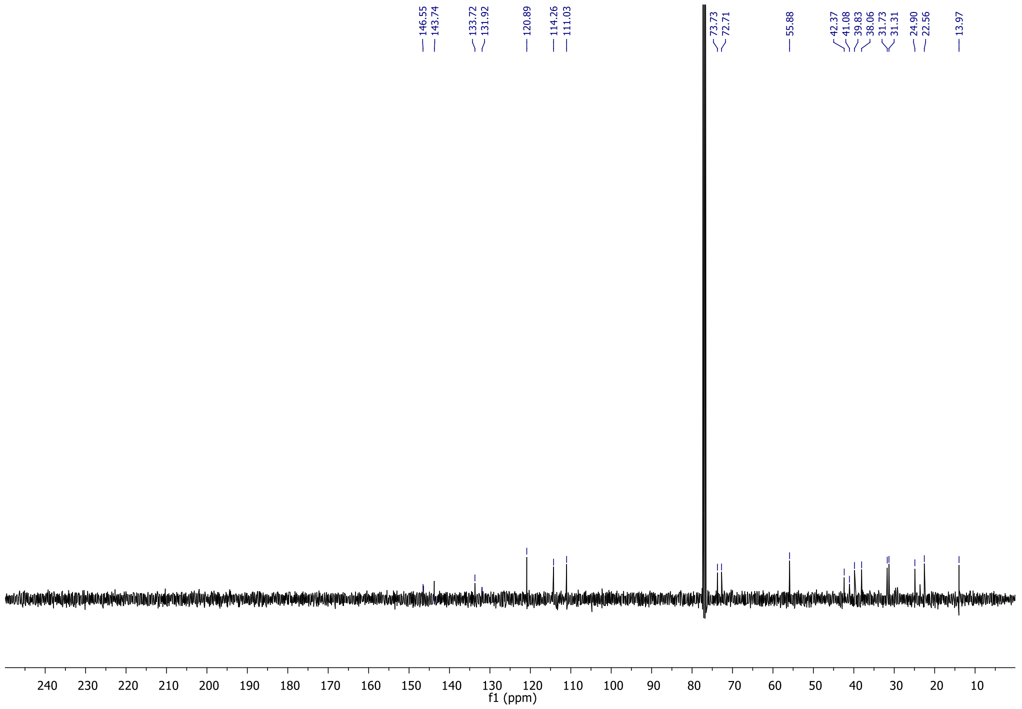

^{13}C-NMR spectra of compound **(*R*)-5a**; 101 MHz, $CDCl_3$

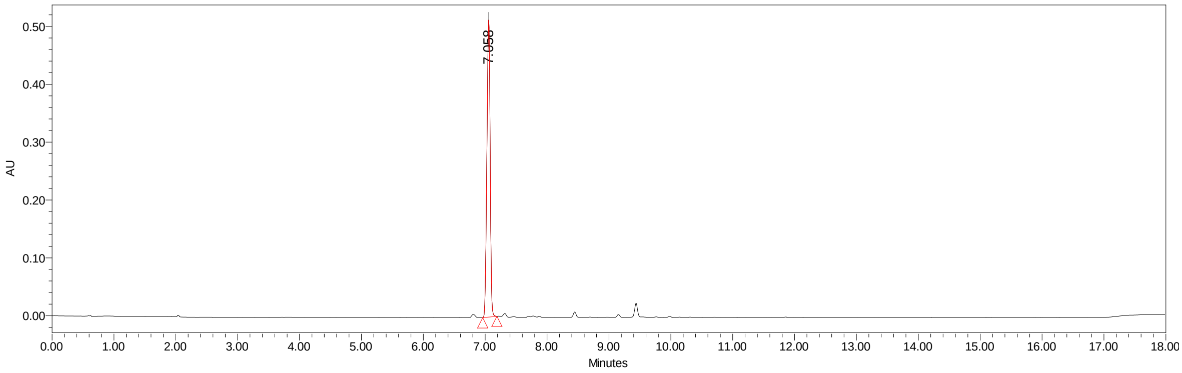

UPLC-UV profile of **(*R*)-5a (Method A)**

8-gingerdiols ((*R*)-5b + (*S*)-5b)

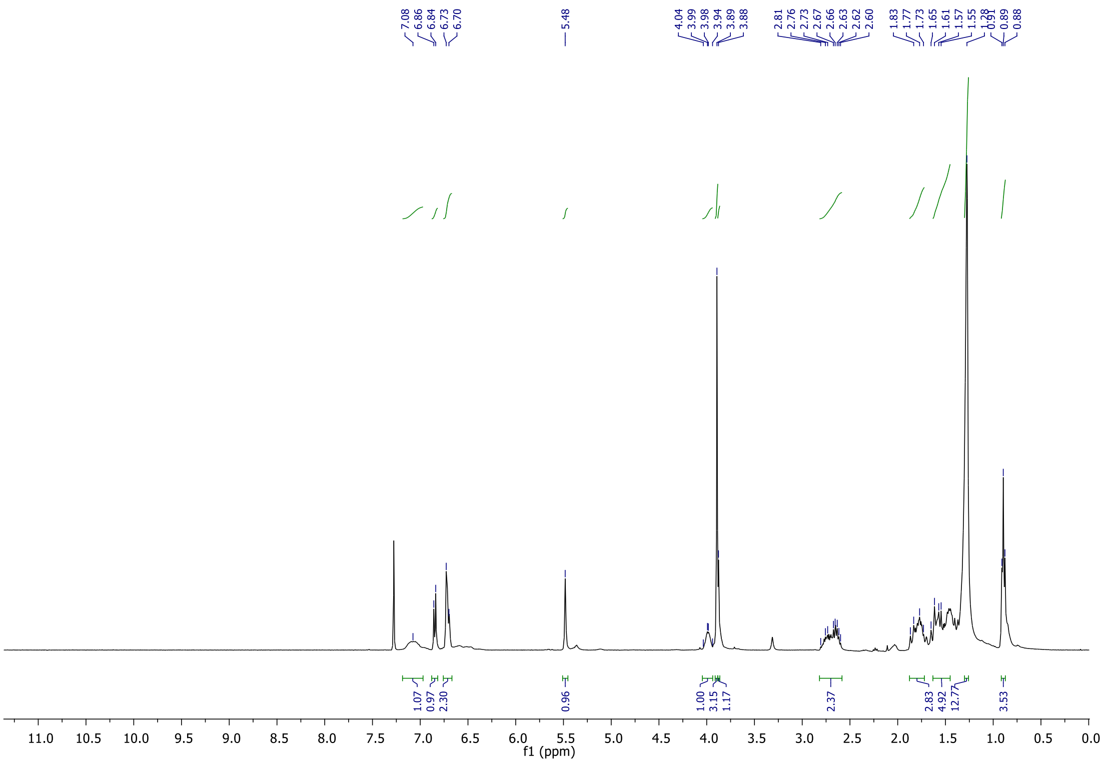

^{1}H-NMR spectra of **(*R*)-5b + (*S*)-5b**; 400 MHz, $CDCl_3$

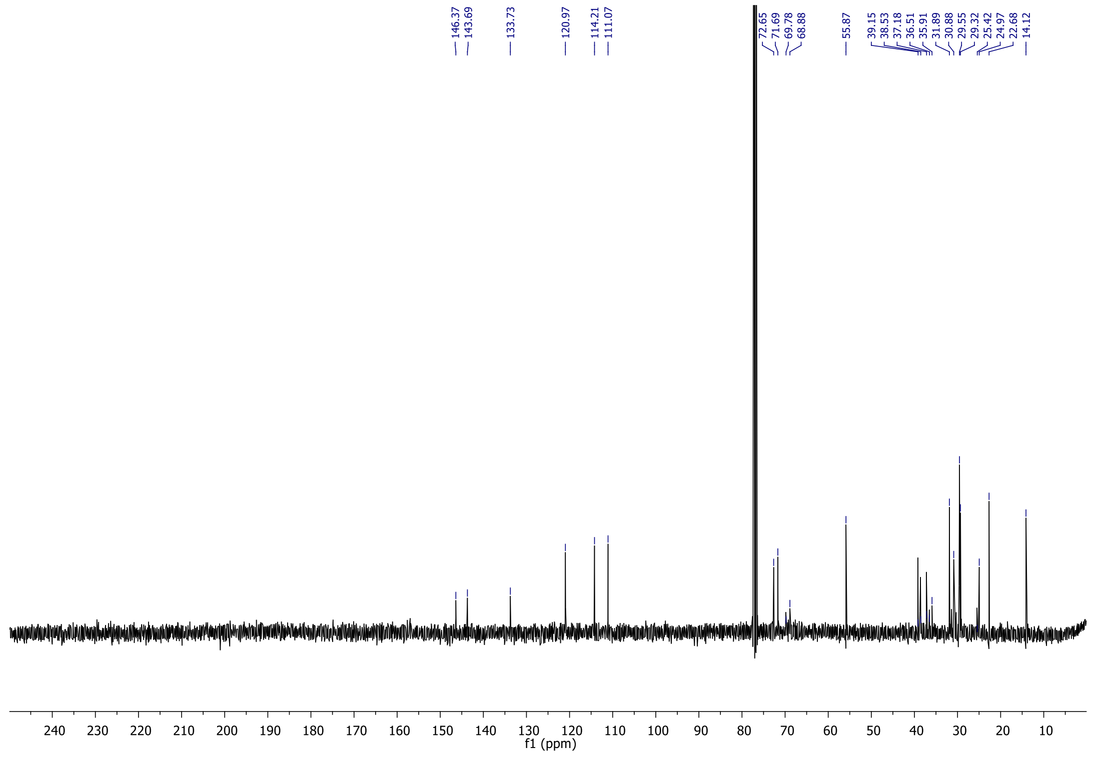

^{13}C-NMR spectra of **(*R*)-5b + (*S*)-5b**, 101 MHz, $CDCl_3$

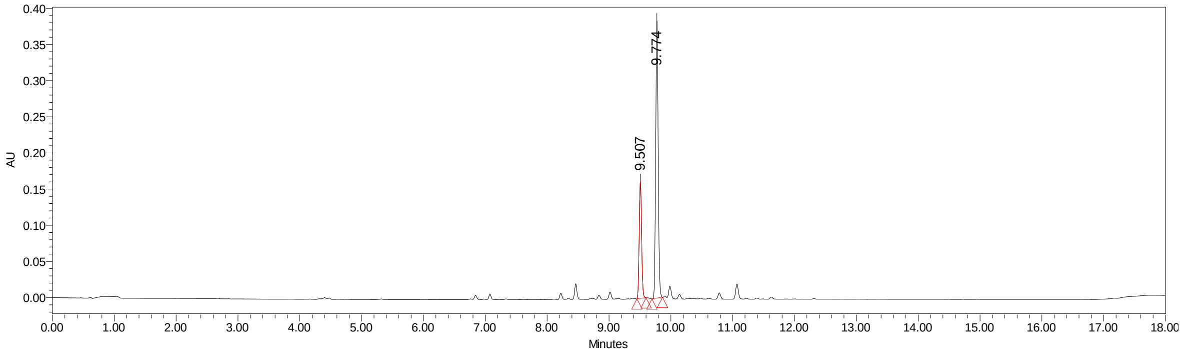

UPLC-UV profile of ***(R)*-5b + *(S)*-5b**

6-dihydroparadol (6, racemic)

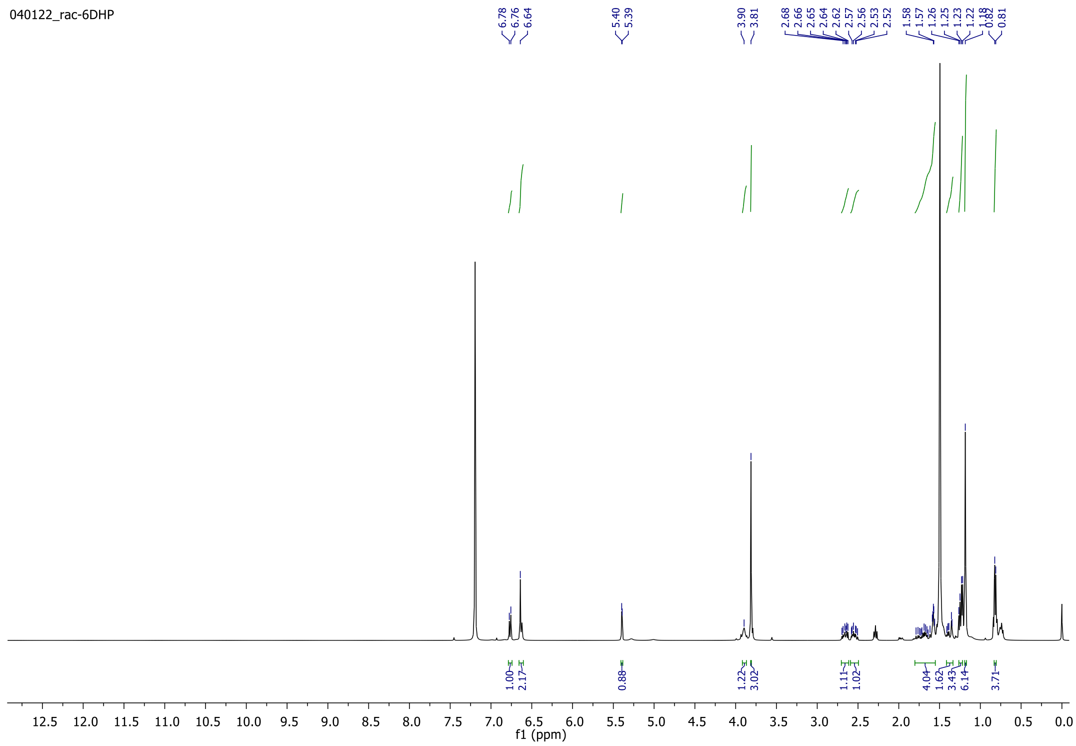

^{1}H-NMR spectra of racemic **6**; 400 MHz, $CDCl_3$

(6*S*)-dihydroparadol ((*S*)-6)

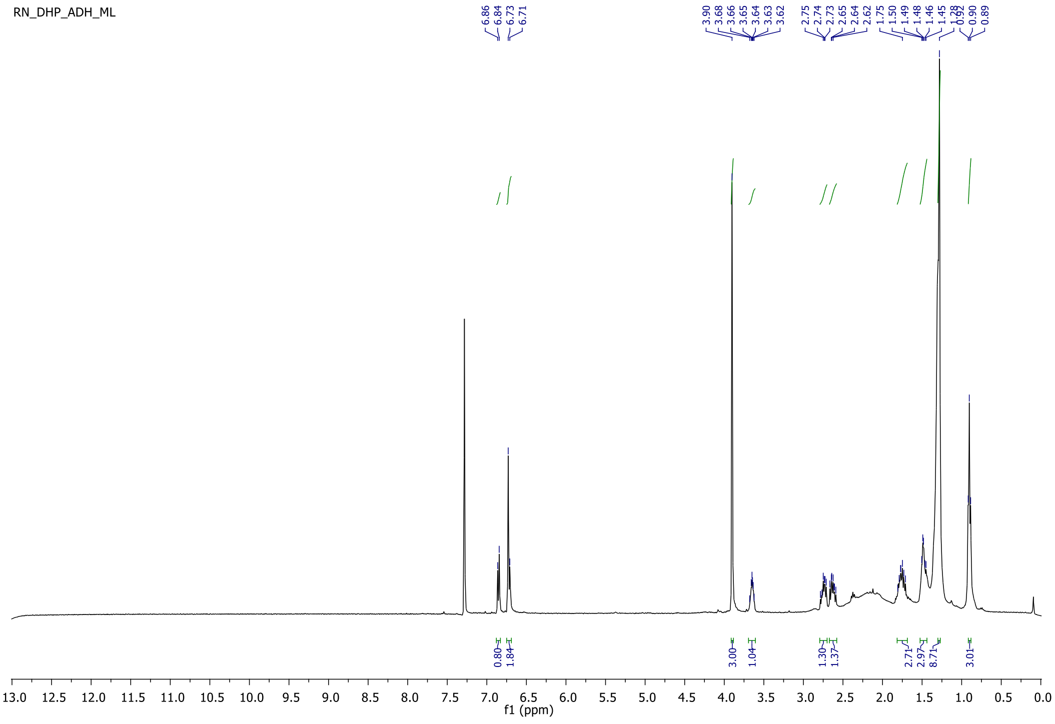

^{1}H-NMR spectra of **(*S*)-6**; 400 MHz, $CDCl_3$

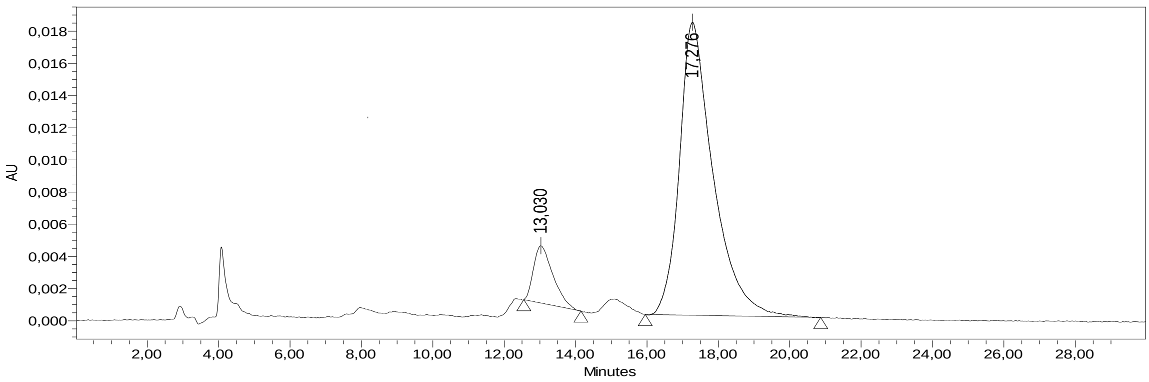

Chiral HPLC profile of **(*S*)-6** (Method B)

Zingerol (7, racemic)

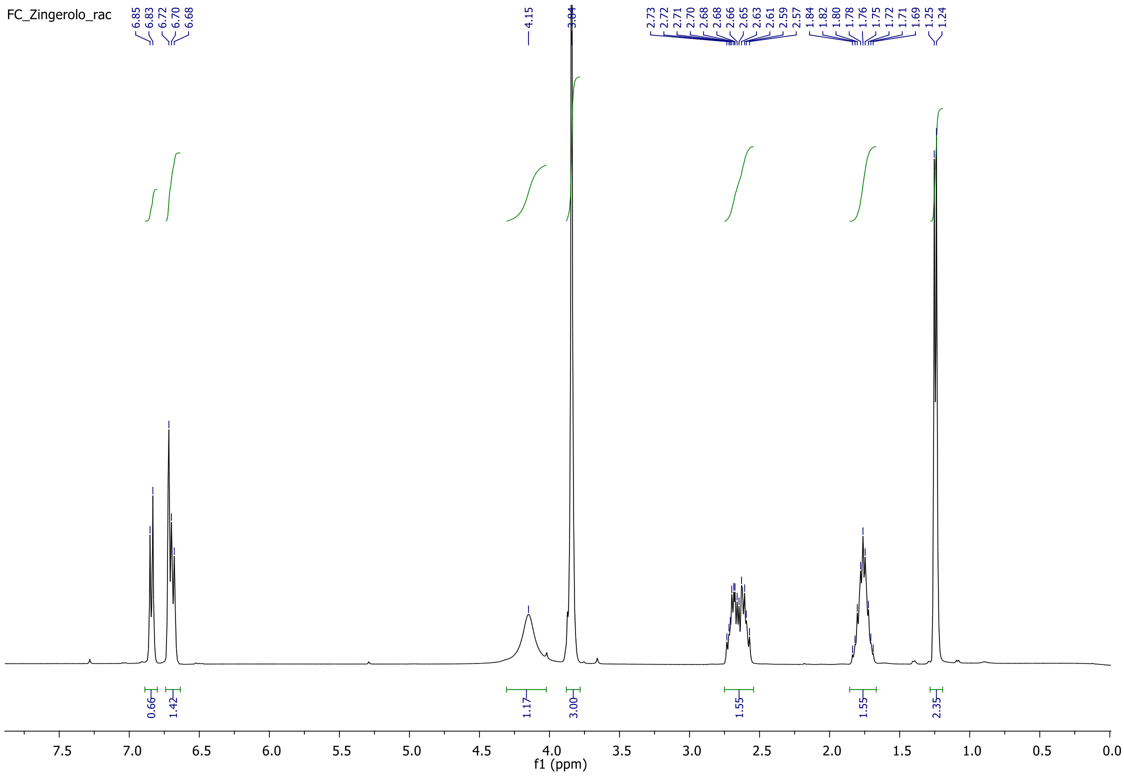

^{1}H-NMR spectra of racemic **7**; 400 MHz, $CDCl_3$

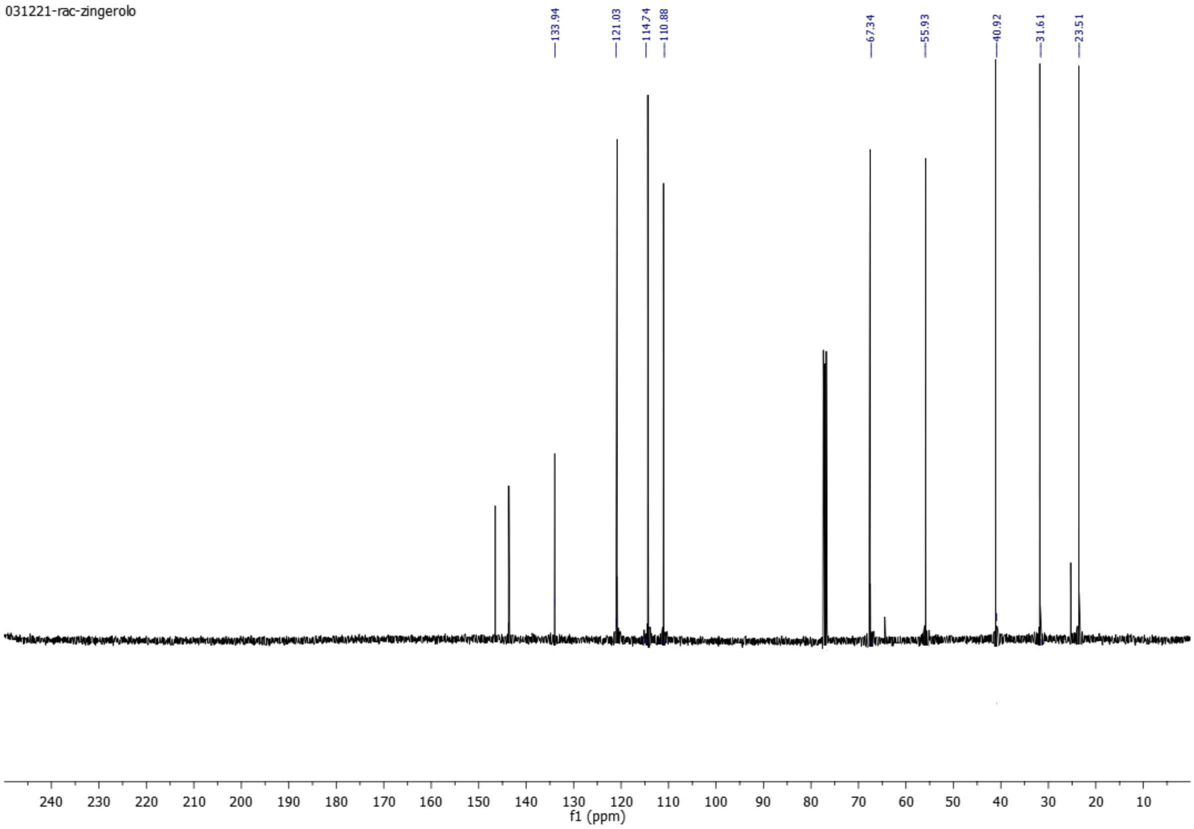

^{13}C NMR spectra of racemic **7**; 101 MHz, $CDCl_3$

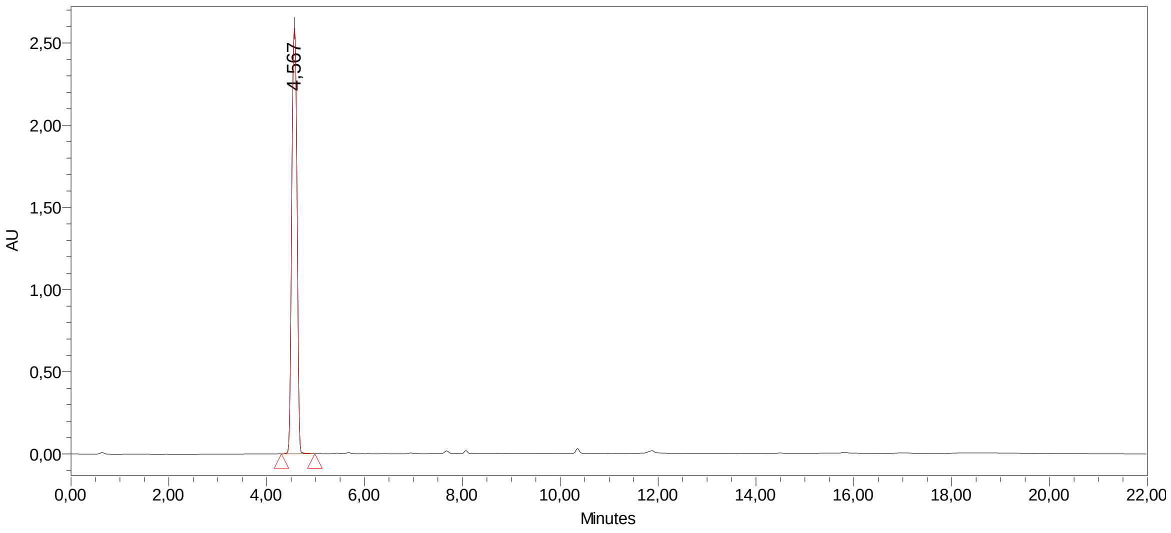

UPLC-UV profile of **7 (Method A)**

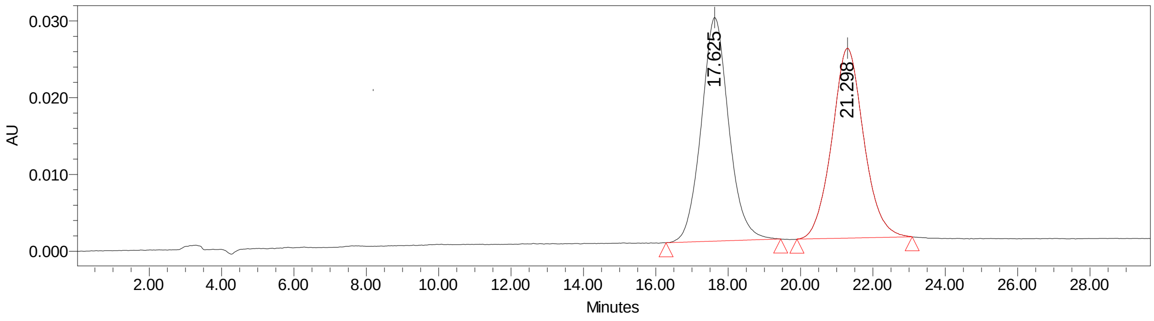

Chiral HPLC profile of **7 (Method B)**

(*R*)-zingerol ((*R*)-7)

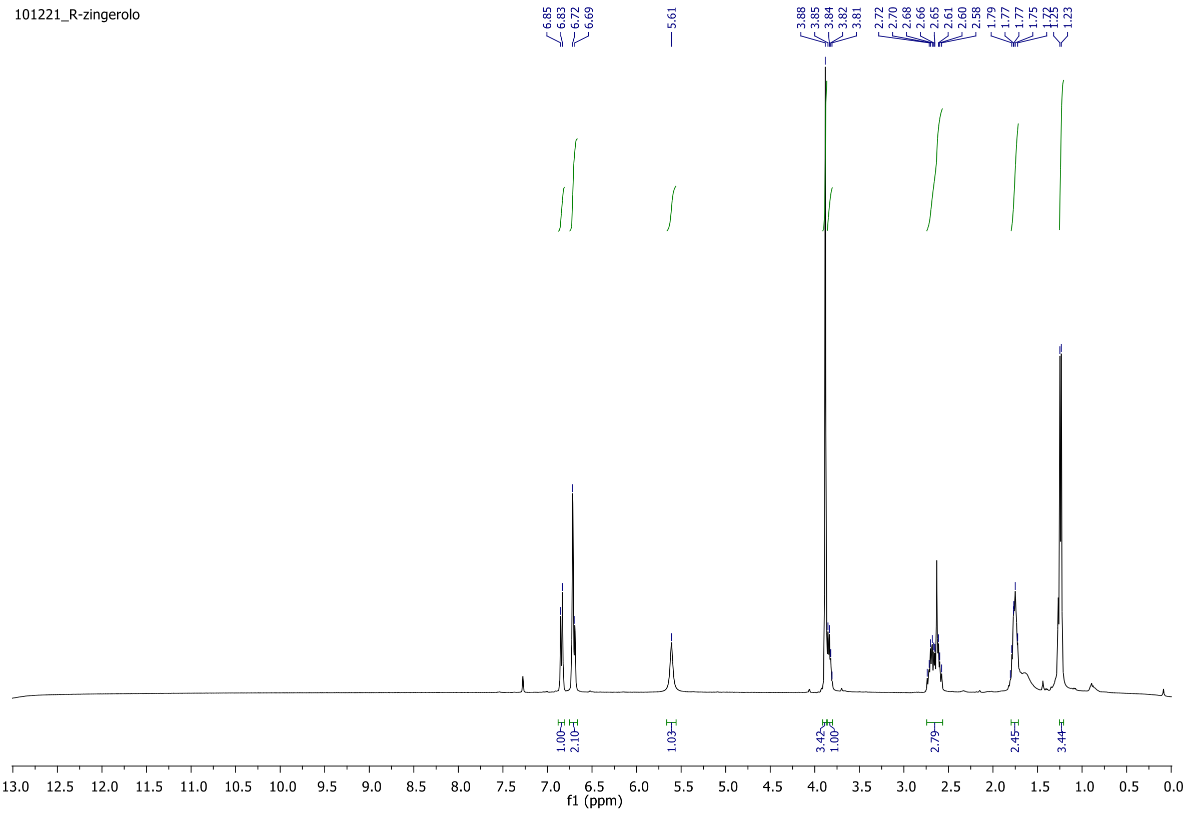

^{1}H-NMR spectra of **(*R*)-7**; 400 MHz, $CDCl_3$

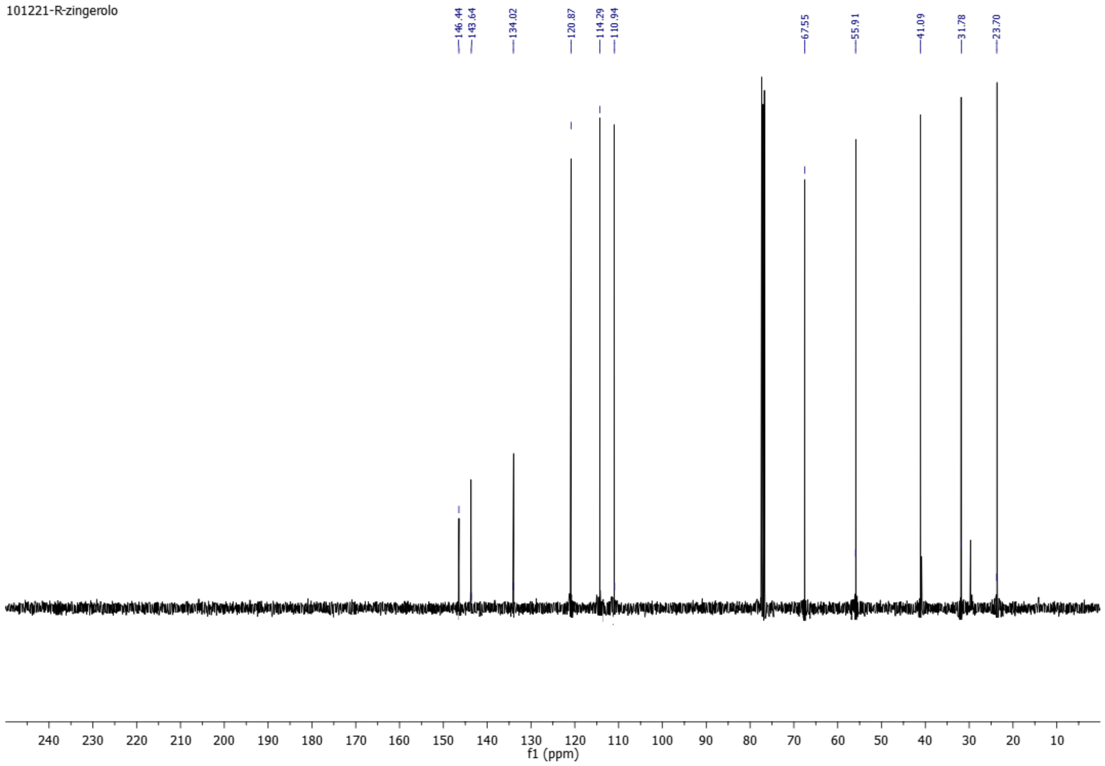

^{13}C-NMR spectra of **(*R*)-7**; 101 MHz, $CDCl_3$

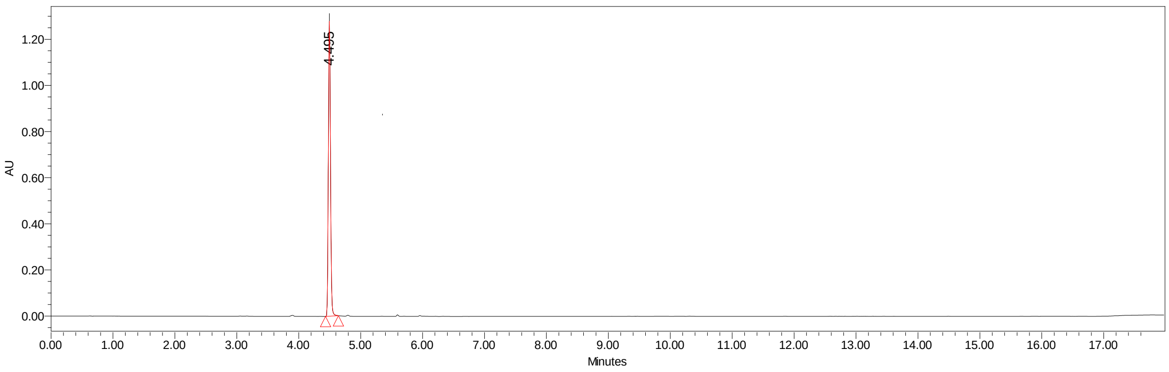

UPLVC-UV profile of **(*R*)-7** (Method A)

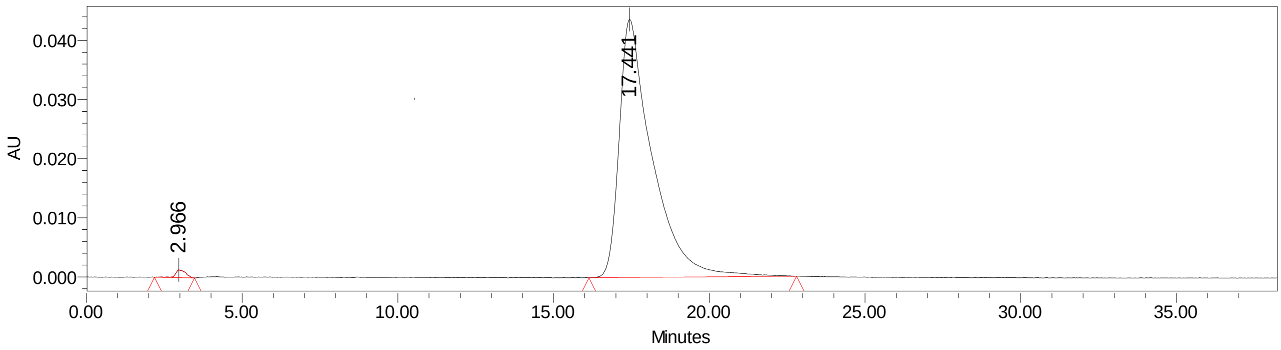

Chiral HPLC profile of **(*R*)-7** (Method B)

(*S*)-Zingerol ((*S*)-7)

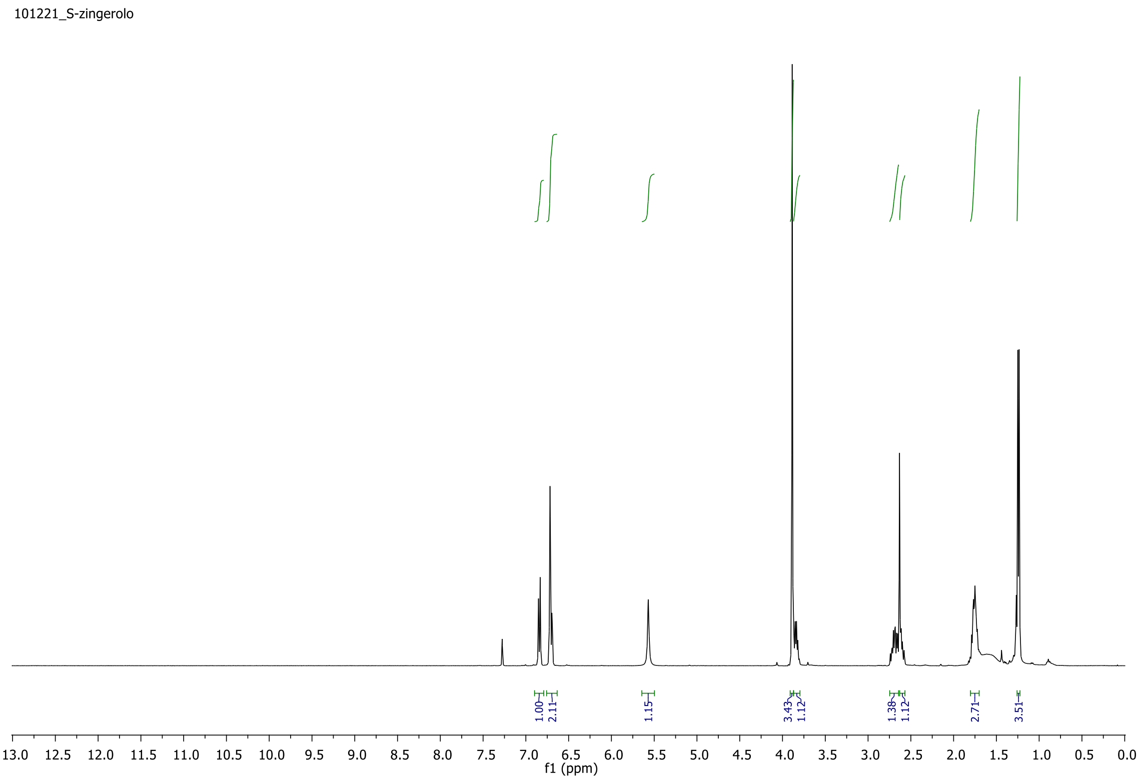

^{1}H-NMR spectra of **(*S*)-7**; 400 MHz, $CDCl_3$

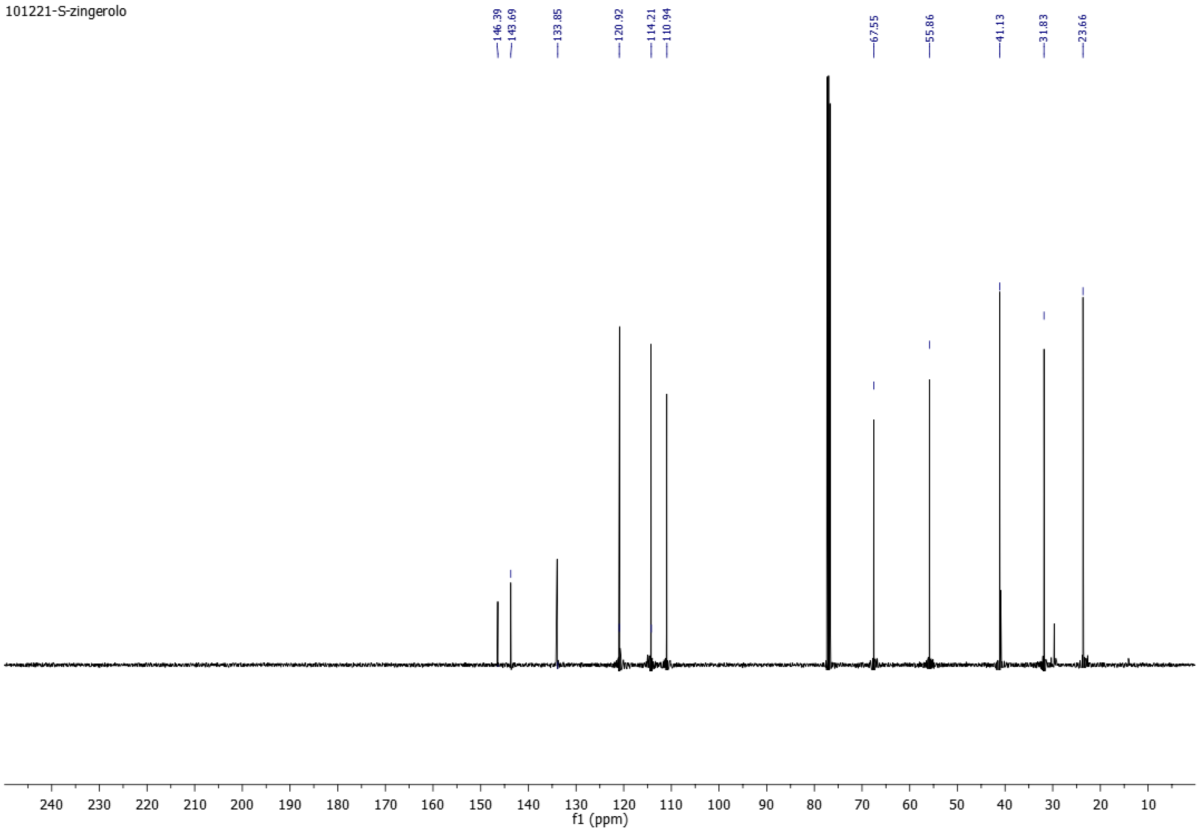

^{13}C-NMR spectra of **(*S*)-7**; 101 MHz, $CDCl_3$

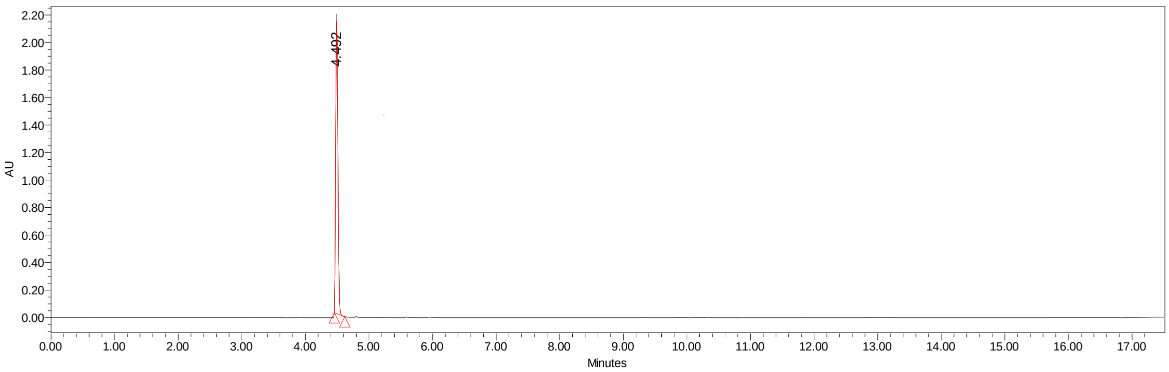

UPLVC-UV profile of **(*S*)-7** (Method A)

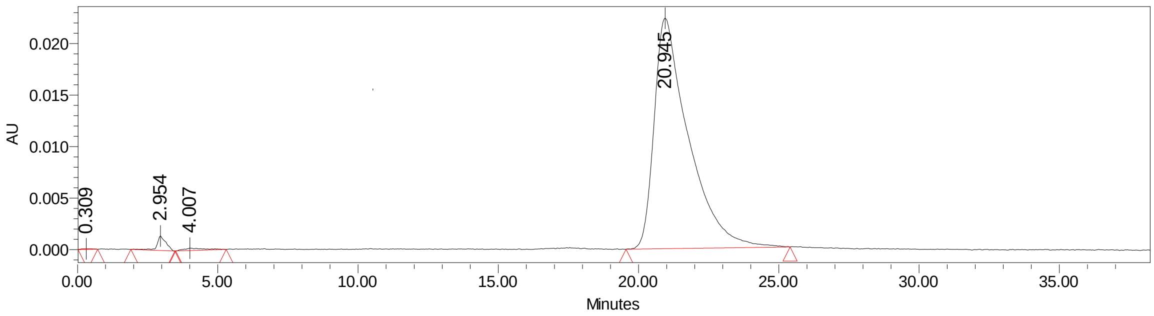

Chiral HPLC profile of **(*S*)-7** (Method B)

9) Asymmetric reduction of 4 *via* (*R*)-(+)-2-methyl-CBS-oxazaborolidine catalyst

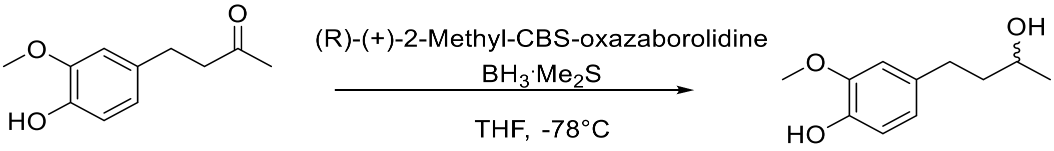

Experimental procedure

4-(4-hydroxy-3-methoxyphenyl)butan-2-one **4** (151.0 mg, 0.78 mmol, 1 eq) was dissolved in dry THF (7.5 mL) and the reaction vessel was cooled to -78°C under stirring. After 15 min, (*R*)-(+)-2-Methyl-CBS-oxazaborolidine (0.078 ml, 0.078 mmol, 0.1 eq) was added to the reaction mixture and stirred for another 30 min. Later, $BH_3·Me_2S$ (0.047 ml, 0.47 mmol, 0.6 eq) was added dropwise at -78°C and the reaction temperature was allowed to warm to room temperature. The reaction was monitored by TLC (*n*-Hex-EtOAc, 6:4, v:v) for 16 h showing the partial conversion of the starting material (Rf 0.46) into the product (Rf 0.27). Then, water (2 mL) was added followed by ethyl acetate (5 mL) and the organic phase was extracted. The crude product was purified by chromatography gravity column eluting with *n*-Hex-EtOAc (6:4, v:v) as eluent to afford racemic *4-(3-hydroxybutyl)-2-methoxyphenol* **7** as a colourless oil (10.3%). Rf 0.46 (n-Hex-EtOAc 6:4, v:v).

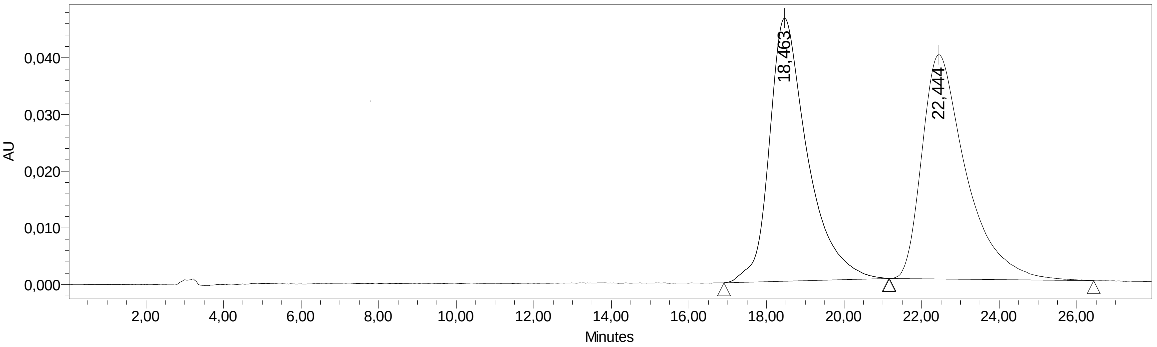

Chiral HPLC profile of zingerol (**7**) from (*R*)-CBS catalytic reduction (**Method B**).

References

[1] J.-W. Song, E.-Y. Jeon, D.-H. Song, H.-Y. Jang, U. T. Bornscheuer, D.-K. Oh, J.-B. Park, *Angew. Chemie Int. Ed.* **2013**, *52*, 2534–2537.

[2] A. Weckbecker, W. Hummel, *Biocatal. Biotransformation* **2006**, *24*, 380–389.

[3] K. Edegger, W. Stampfer, B. Seisser, K. Faber, S. F. Mayer, R. Oehrlein, A. Hafner, W. Kroutil, *European J. Org. Chem.* **2006**, *2006*, 1904–1909.

[4] K. Niefind, J. Müller, B. Riebel, W. Hummel, D. Schomburg, *J. Mol. Biol.* **2003**, *327*, 317–328.

[5] S. Bertuletti, E. E. Ferrandi, S. Marzorati, M. Vanoni, S. Riva, D. Monti, *Adv. Synth. Catal.* **2020**, *362*, 2474–2485.

[6] E. E. Ferrandi, G. M. Bertolesi, F. Polentini, A. Negri, S. Riva, D. Monti, *Appl. Microbiol. Biotechnol.* **2012**, *95*, 1221–1233.

[7] T. Yoshimoto, H. Higashi, A. Kanatani, X. S. Lin, H. Nagai, H. Oyama, K. Kurazono, D. Tsuru, *J. Bacteriol.* **1991**, *173*, 2173–2179.

[8] L. Liu, A. Aigner, R. D. Schmid, *Appl. Microbiol. Biotechnol.* **2011**, *90*, 127–35.

[9] R. D. Schmid, M. Braun, L. Liu, A. Aigner, D. Weuster-Botz, *7 α-Hydroxysteroid Dehydrogenase Knockout Mutants and Use Therefor*, **2011**, US9096825B2.

[10] F. W. Studier, *Protein Expr. Purif.* **2005**, *41*, 207–234.

[11] W. Koch, W. Kukula-Koch, Z. Marzec, E. Kasperek, L. Wyszogrodzka-Koma, W. Szwerc, Y. Asakawa, *Int. J. Mol. Sci.* **2017**, *18*, 452.

[12] Y. Li, Y. Hong, Y. Han, Y. Wang, L. Xia, *J. Chromatogr. B* **2016**, *1011*, 223–232.
